# The successor representation in high-risk drinking and alcohol-related contexts

**DOI:** 10.1371/journal.pcbi.1014873

**Published:** 2026-10-21

**Authors:** Milena P. M. Musial, Sam Hall-McMaster, Kanji Shimomura, Ayaka Kato, Erik L. Bode, Clarissa C. Grundmann, Claudia Ebrahimi, Kenji Morita, Samuel J. Gershman, Tanja Endrass, Florian Schlagenhauf

**Affiliations:** 1 Charité – Universitätsmedizin Berlin, corporate member of Freie Universität Berlin and Humboldt-Universität zu Berlin, Department of Psychiatry and Neurosciences | CCM, NeuroCure Clinical Research Center, Berlin, Germany; 2 Humboldt-Universität zu Berlin, Faculty of Life Sciences, Department of Psychology, Berlin, Germany; 3 Charité – Universitätsmedizin Berlin, corporate member of Freie Universität Berlin and Humboldt-Universität zu Berlin, Einstein Center for Neurosciences Berlin, Berlin, Germany; 4 Charité – Universitätsmedizin Berlin, corporate member of Freie Universität Berlin and Humboldt-Universität zu Berlin, Bernstein Center for Computational Neuroscience, Berlin, Germany; 5 Department of Psychology and Center for Brain Science, Harvard University, Cambridge, Massachusetts, United States of America; 6 Graduate School of Education, The University of Tokyo, Tokyo, Japan; 7 Japan Society for the Promotion of Science, Tokyo, Japan; 8 Department of Information Medicine, National Institute of Neuroscience, National Center of Neurology and Psychiatry, Tokyo, Japan,; 9 Department of Psychiatry, Icahn School of Medicine at Mount Sinai, New York, New York, United States of America; 10 Department of Psychiatry and Behavioral Sciences, Graduate School of Medical and Dental Sciences, Institute of Science Tokyo, Tokyo, Japan; 11 Well-Vitality Visionary Initiative, Institute of Science Tokyo, Tokyo, Japan,; 12 Institute of Clinical Psychology and Psychotherapy, Technische Universität Dresden, Dresden, Germany; 13 International Research Center for Neurointelligence (WPI-IRCN), The University of Tokyo, Tokyo, Japan; Pantheon-Sorbonne University: Universite Paris 1 Pantheon-Sorbonne, FRANCE

## Abstract

The successor representation (SR) is a reinforcement learning strategy which has been suggested to underlie nuanced forms of habitual behavior, rendering it a candidate mechanism for mental health conditions thought to involve habitual control, such as addiction. However, paradigms used to assess habits in humans cannot detect SR use. Together with the fact that they are usually conducted in disorder-irrelevant contexts, this may explain why the evidence for a hypothesized shift from goal-directed to habitual behavior in addiction is inconsistent. Here, we tested whether individuals with high-risk (*n* = 260) compared to low-risk drinking behavior (*n* = 260) rely more on SR or a rigid goal-based dimension-reduced SR variant (redSR), particularly in alcohol-related contexts. An online multi-stage decision-making task performed in either a virtual bar or apartment context allowed us to dissociate (red)SR from model-based and model-free learning strategies by testing how well participants adapted their choices to different types of distal changes in the environment. Findings suggest that a particular type of SR, namely a (reduced) random-policy SR-like strategy, contributes to human behavior, but that high-risk drinkers do not differ from low-risk drinkers in their use of this strategy. Instead, both groups seem to rely less on what is likely a (reduced) random-policy SR strategy in alcohol-related contexts – and instead more on model-free control. Results suggest that the use of an adaptive, resource-efficient decision-making strategy is selectively downregulated in substance-related contexts. This study highlights the importance of considering contextual modulation and nuanced decision strategies in understanding habitual and goal-directed behavior in mental health conditions such as addiction.

## Author summary

Humans can behave in more habitual or more goal-directed ways, like when routinely brushing our teeth versus planning how to circumvent a construction site. Mental health conditions such as addiction have been suggested to involve a shift from goal-directed to habitual behavior, but the evidence is inconsistent. This might be due to how habits have been assessed in the laboratory: tasks usually take place in contexts unrelated to the disorder and cannot detect the use of strategies with subtle characteristics of habitual behavior. Here, we set out to determine the role of one such strategy, the successor representation, in high-risk drinking and alcohol-related contexts. While we found no increased use of this strategy in high-risk drinking, individuals likely relied less on a successor representation-based strategy in a virtual bar environment compared to a non-alcohol-related context. Drug-associated settings thus seem to be associated with diminished reliance on a generally adaptive, efficient behavioral strategy. Our findings highlight that, to understand the role of habits in mental health conditions, we need to consider decision-making strategies along a continuum between goal-directed and habitual control and, importantly, disorder-relevant contexts.

## Introduction

When we unlock our phone’s screen, we often do so in a quick and automatic way, not even planning to do anything specific on our device. This can be considered a habit [1] as it is inflexible, triggered by preceding stimuli, and does not depend on knowledge about action-outcome associations [1,2]. While habits save cognitive resources [3], they have been suggested to underlie repetitive behavior in mental health conditions, in particular substance use disorders [4–8]. The habit theory of addiction suggests that drug-related behavior is initially goal-directed but becomes more independent from its consequences and thus more habitual when transitioning to addiction [4,6,7].

Despite its intuitive appeal, evidence for this theory in humans is mixed [2,9–11], which might be due to how habits are usually measured. Whereas outcome devaluation and contingency degradation paradigms quantify habits as continued responding for an outcome which has been devalued or has become less contingent on the action [1], habitual and goal-directed behavior in the two-stage sequential decision-making task are often approximated by model-free (MF) and model-based (MB) reinforcement learning (RL), respectively [12–14] (but see [15]). Using these paradigms in addiction research comes with two limitations. First, they are conducted in disorder-irrelevant contexts, assuming that individuals with substance use disorders display increased habitual behavior beyond drug-related situations. Given that the central supposedly habitual behavior in addiction is drug-associated and that habits are context-dependent [16], this assumption is questionable. Second, previous paradigms conceptualize behavior as either goal-directed, habitual, or a mixture of both, neglecting other behavioral strategies [17].

One candidate strategy leading to more subtle habit-like behavior than that captured by MF RL or devaluation- or contingency-insensitive responding is the successor representation (SR) [18]. SR algorithms lie at an intermediate position between MB and MF RL in terms of behavioral flexibility and computational efficiency [19]. Unlike MF algorithms, which update the state value function under a given policy π directly, SR algorithms use a multi-step predictive matrix $M^{\pi }$ representing each state by the discounted future occupancies of its successor states [19–21]. To illustrate, imagine a person who usually drives their car home after work, but sometimes takes the subway instead to go for drinks. If they use SR RL, they represent their workplace by how often, starting from there, they anticipate visiting each successor state (car, home, subway, bar) – no matter via which other states they got there. At decision time, they can then derive a value estimate for state s by a simple linear combination of the elements in row s of $M^{\pi }$ and the rewards received per successor state s' contained in a reward vector R (which can be approximated by weight vector w [22,23]; see Methods). MB learners, by contrast, represent the environment via a one-step transition matrix T and need to iteratively combine knowledge about one-step transition probabilities (e.g., office-subway, subway-bar) with the respective elements in R to derive state value estimates.

SR’s resource-efficient value estimation allows for flexible behavior under many circumstances. Like MB and unlike MF algorithms, SR RL can adapt to distal changes in reward (*reward revaluation*), also when these changes occur in a state which previously contained no reward (*goal-state revaluation*): even when changes were not experienced starting from the current state (i.e., changes were distal), they can be reflected in w and can be linearly combined with the current state’s row of $M^{\pi }$, resulting in updated value estimates.

However, SR use leads to habit-like behavior under certain other conditions: like MF and unlike MB algorithms, SR learners are unsuccessful in *policy revaluation*, meaning they cannot adapt to distal changes in rewards in states which have rarely been visited starting from the current state, e.g., because they contained no reward and the agent followed a near-optimal policy [21,22]. The new reward can be represented in w but will be multiplied by a discounted future occupancy near zero, leading to negligible effects on the current state’s value estimate. Moreover, like MF and unlike standard MB algorithms, SR learners also cannot adapt to *transition revaluation*, i.e., distal changes in the transition structure [21, 22], as transition experiences only affect the rows of $M^{\pi }$ corresponding to their predecessor states. If altered transitions have not been experienced starting from the current state, they will not be reflected in the corresponding row of $M^{\pi }$.

These inflexibilities are why SR RL has been described as a computational basis for a “subtler, more cognitive notion of habit” [22 p. 680] than that produced by MF algorithms. However, since outcome devaluation and contingency degradation paradigms induce proximal changes in the reward structure and since the two-step paradigm induces a type of reward revaluation, SR use leads to seemingly goal-directed behavior in these tasks [21]. Inconsistent evidence for increased habitual responding in addiction does thus not preclude that individuals with high-risk drug taking behavior or addiction rely more on SR strategies, a potential formalization of more subtle habitual behavior, than low-risk or non-addicted individuals [22,24,25].

A variant of the SR which is theoretically appealing in the addiction context is rigid goal-based reduced SR (redSR) [23]. Dimension reduction of the SR has been suggested to conserve computational resources in large state spaces [20,23]. While there is no definite answer to how relevant dimensions are selected [26], one option is a predictive map $M_{red}^{\pi }$ which represents each state only by the expected future occupancies of salient goal-states [23]. When $M_{red}^{\pi }$ is additionally insensitive to updates, this variant of the SR is called redSR [23]. As suggested by Shimomura et al. [23], a rigid $M_{red}^{\pi }$ could be established after prolonged drug seeking without occasional resistance, with goal-states defined as states previously associated with drug reward. To illustrate, somebody using this strategy would represent their workplace only by how often they expect to visit the bar, but not any other successor state (e.g., car, home, subway).

RedSR is of interest in an addiction context for two reasons. First, compared to SR, it has enhanced habit-like characteristics: while redSR maintains sensitivity to rewards in goal-states, it cannot reliably adapt to goal-state revaluation as $M_{red}^{\pi }$ lacks columns representing non-goal-states’ discounted future occupancies, and the dimension-reduced weight vector $w_{red}$ lacks elements for non-goal-states [23]. Second, in simulations, Shimomura et al. [23] demonstrated that redSR agents exhibit several characteristics supposed to underlie addicted behavior once they “tried” to resist reaching drug-associated goal-states. For instance, redSR agents showed sustained positive reward prediction errors (RPEs) upon finally reaching a goal-state, resembling drug-induced RPE signals hypothesized to reinforce drug taking [27]. Such sustained RPEs emerge due to incorrect value approximation based on a rigid, dimension-reduced predictive map $M_{red}^{\pi }$ [23,28] (Methods).

Despite the habit-like characteristics of SR and despite redSR’s phenotypic similarities to addiction [23], no study to date has investigated the extent to which individuals with high-risk drinking use SR or redSR and, importantly, whether the use of these strategies is more pronounced in drug-related contexts. One way to dissociate SR, redSR, MF, and MB strategies is by exploiting each algorithm’s unique ability to adapt to reward, goal-state, policy, or transition revaluation [21–23]. When individuals experience all types of revaluation, we can test for signatures of SR use (higher success in reward and goal-state compared to transition and policy revaluation) and of redSR use (higher success in reward than in goal-state revaluation) as opposed to standard MB and MF use (comparably high or low success across all revaluation types, respectively) [22]. To do so in a context-specific manner, we developed an active multi-stage decision-making paradigm based on a task introduced by Momennejad and colleagues [22] in a bar setting and an apartment setting, respectively. In a preregistered (https://doi.org/10.17605/OSF.IO/9TUZE) cross-sectional online study, *N =* 520 alcohol users with high-risk (*n* = 260) and low-risk (*n* = 260) drinking behavior according to the Alcohol Use Disorders Identification Test (AUDIT) [29,30] performed our task in either the alcohol-related or non-alcohol-related context.

First, we aimed to replicate previous findings of SR use in humans [22] in a more lifelike task environment. Specifically, we hypothesized that 1) low-risk drinkers use an SR strategy to some extent when learning in a non-alcohol context, and that 2) their behavior cannot be explained by an alternative MB strategy with different learning rates for rewards and transitions [22]. Based on the habit theory of addiction [4,6,7] and SR’s inflexible properties [22], we further expected that 3) high-risk drinkers use SR to a larger degree than low-risk drinkers across contexts, but 4) particularly in an alcohol compared to a non-alcohol context. Lastly, given the resemblance of redSR-produced phenomena and addiction symptoms [23], we hypothesized that 5) in an alcohol context, high-risk drinkers rely more strongly on redSR compared to low-risk drinkers, and that 6) this group difference is more pronounced than in a non-alcohol context.

We found that human behavior cannot be explained by use of standard MB, MF, or hybrid standard MB-MF strategies alone [12,22]. Instead, while an alternative MB strategy with a higher learning rate for rewards than transitions offered a potential explanation, humans likely use a form of SR, specifically a representation similar to a (goal-based dimension-reduced) random-policy SR [31,32]. Different than expected, what is likely a (reduced) random-policy SR was not used more by high-risk drinkers but was instead used less in alcohol-related contexts, where low-risk as well as high-risk drinkers instead seemed to rely more strongly on an inflexible MF strategy. The (reduced) random-policy SR-like strategy thus appears to offer an adaptive, resource-efficient representation of the environment which is used less in contexts that have previously been associated with drug use. While the factors that mediate this effect remain to be further elucidated, these findings underscore the necessity to consider contextual effects when assessing habitual behavior and cognitive representations in humans.

## Results

### SR, redSR, standard MB, and MF agents behave differently in a multi-stage decision-making task

#### Task design.

Participants performed the active multi-stage sequential decision-making paradigm (Fig 1A, Fig A in S1 Text) in either an alcohol context, i.e., a virtual bar with the goal to collect as many glasses of an individually preferred alcoholic drink as possible, or a non-alcohol context, i.e., a virtual apartment with the goal to collect as much hidden cash as possible (Fig 1B). The task consisted of five conditions. In each condition, in an initial learning phase, participants had the chance to learn which out of four paths through a deterministic 10-state environment was optimal, i.e., led to the highest reward. They were excluded if they failed to favor the optimal path by the end of this phase in any condition (Methods). While all learning phase trials started from one initial state, trials in a subsequent re-learning phase started in later states. Here, participants experienced changes to the environment’s reward structure (in reward, goal-state, policy revaluation conditions, and the control condition) or transition structure (in the transition revaluation condition) which, in revaluation conditions, implied a change in optimal path departing from the environment’s initial state. To assess how well participants adapted their path preference to the distal changes experienced during re-learning, we let them start from the initial state again in a subsequent test phase, with the instruction to maximize reward. The probability of changing path preference on such a test phase trial compared to the optimal path in the learning phase served as the primary measure of revaluation performance. A final rating phase, where participants were asked to indicate expected reward per initial-state-action, delivered a secondary, more deliberate measure of revaluation performance.

**Fig 1:**
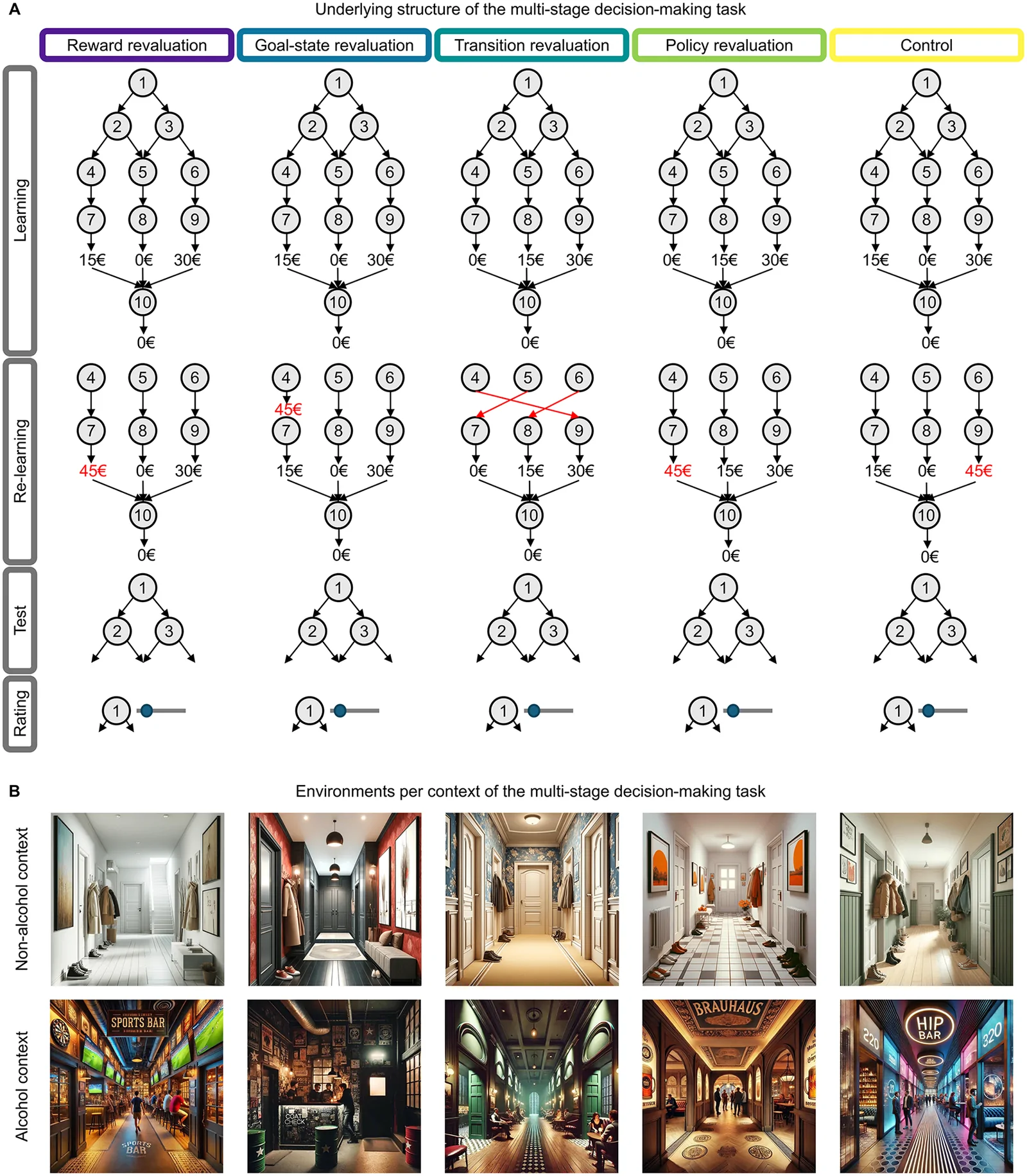
Multi-stage decision-making task. **A** Participants performed five task conditions (columns) encompassing four phases (rows) each. In each condition, they navigated through an environment consisting of 10 states (numbered circles) arranged in five stages and were instructed to maximize reward while staying vigilant to changes. In each state, participants had to perform an action (arrows) which deterministically led to a reward (€ amounts) and a subsequent state (except for state 10). A path through the environment was determined by the combination of state-1- and state-2- or state-3-actions. During each phase, participants visited only the states shown. The initial learning phase encompassed 24 trials starting from state 1, out of which the first four were forced-choice, guaranteeing that each path was experienced once. Changes to the environment only occurred in the subsequent re-learning phase, where all nine trials started in third-stage states (three trials from state 4, 5, and 6 each). In the reward revaluation condition, a new high reward was introduced following an action in a state which previously led to a small reward and should thus have been visited with moderate frequency starting from state 1 during learning under a near-optimal policy. This condition implied a change in optimal state-1-action. In the goal-state revaluation condition, a new high reward was introduced following an action in a state which previously contained no reward but was positioned on the path to a small-reward-containing state and should thus also have been visited with moderate frequency starting from state 1 during learning under a near-optimal policy. Like reward revaluation, this condition implied a change in optimal state-1-action. In the transition revaluation condition, participants experienced a change in transitions between all third- and fourth-stage states. This condition implied a change in optimal state-1-action due to a change in the optimal second-stage action. In the policy revaluation condition, a new high reward was introduced in a state which previously contained no reward, was not positioned on a path to a reward-containing state, and should thus have rarely been visited starting from state 1 during learning under a near-optimal policy. Like transition revaluation, this condition also implied a change in optimal state-1-action due to a change in the optimal second-stage action, but due to changes in the reward instead of the transition structure. The new reward introduced in the control condition did not imply a change in optimal path. Revaluation performance was assessed based on the subsequent five-trial test phase, where participants were instructed to maximize reward starting from state 1. Test trials ended after the second-stage decision, meaning participants received no feedback on whether they had chosen the optimal path. In a final rating phase delivering a secondary measure of revaluation performance, participants were sequentially presented once with both state-1-actions and asked to indicate how much reward each action would lead to. Tasks in the alcohol and non-alcohol context followed the same depicted structure with the exception that, in the alcohol context, monetary rewards shown here were replaced by glasses of an individually preferred alcoholic drink (beer, wine, long drinks/cocktails) as follows: 0€ = 0 glasses, 15€ = 1 glass, 30€ = 2 glasses, and 45€ = 3 glasses. **B** Participants performed the multi-stage decision-making task in either an alcohol or a non-alcohol context. In each context, each condition took place in a different environment. Stimulus material, also the state-1-stimuli depicted in panel **B** of this figure, was generated using *DALL-E 3* by *OpenAI*. For full stimulus material and task code, see github.com/agschlagenhauf/SR_in_AUD_behav.git. For exemplary sequences from a trial, see Fig A in S1 Text.

#### Simulations.

To test whether SR, redSR, standard MB, and MF RL produce the hypothesized behavioral signatures in the multi-stage decision-making task (Fig 2A), we simulated each algorithm’s trial-by-trial behavior across all task phases per condition for all combinations of learning rate α=[0.1, 0.3, 0.5, 0.7, 0.9] and time discount factor γ=[0.1, 0.3, 0.5, 0.7, 0.9] (Fig B in S1 Text). Simulations showed that SR and redSR agents often did not prefer the optimal path by the end of the learning phase in our task design, particularly in transition and policy revaluation conditions (for an explanation, see Fig B in S1 Text). After selecting only those simulation runs in which the optimal path preference had been acquired by the end of the learning phase, we could confirm our predictions regarding revaluation performance under certain parameter settings (Fig 2B) but observed nuances when using others (Fig B in S1 Text). Importantly, as expected, neither MF nor standard MB agents produced the hypothesized signatures of SR RL, i.e., higher reward and goal-state compared to transition and policy revaluation performance, respectively, at any parameter setting. However, in our task setup, SR itself produced these signatures only under strong time discounting, i.e., at low γ-values, and showed revaluation failure across all task conditions under weak time discounting (for an explanation, see Fig B in S1 Text). Moreover, a redSR agent treating the two state-action-pairs which led to reward during learning as salient showed the signatures hypothesized to be SR-indicative at low γ-values (for an explanation, see Methods, Fig B in S1 Text). While this redSR agent still exhibited its hypothesized signature, i.e., higher performance in reward compared to goal-state revaluation, at α=0.9 and γ=[0.3, 0.5, 0.7], it could thus achieve flexibility comparable to SR RL despite its dimension-reduced form under other parameter settings in our task. Note that the latter was also the case when assuming that redSR considers additional goal-state-action-pairs (Fig C in S1 Text).

**Fig 2:**
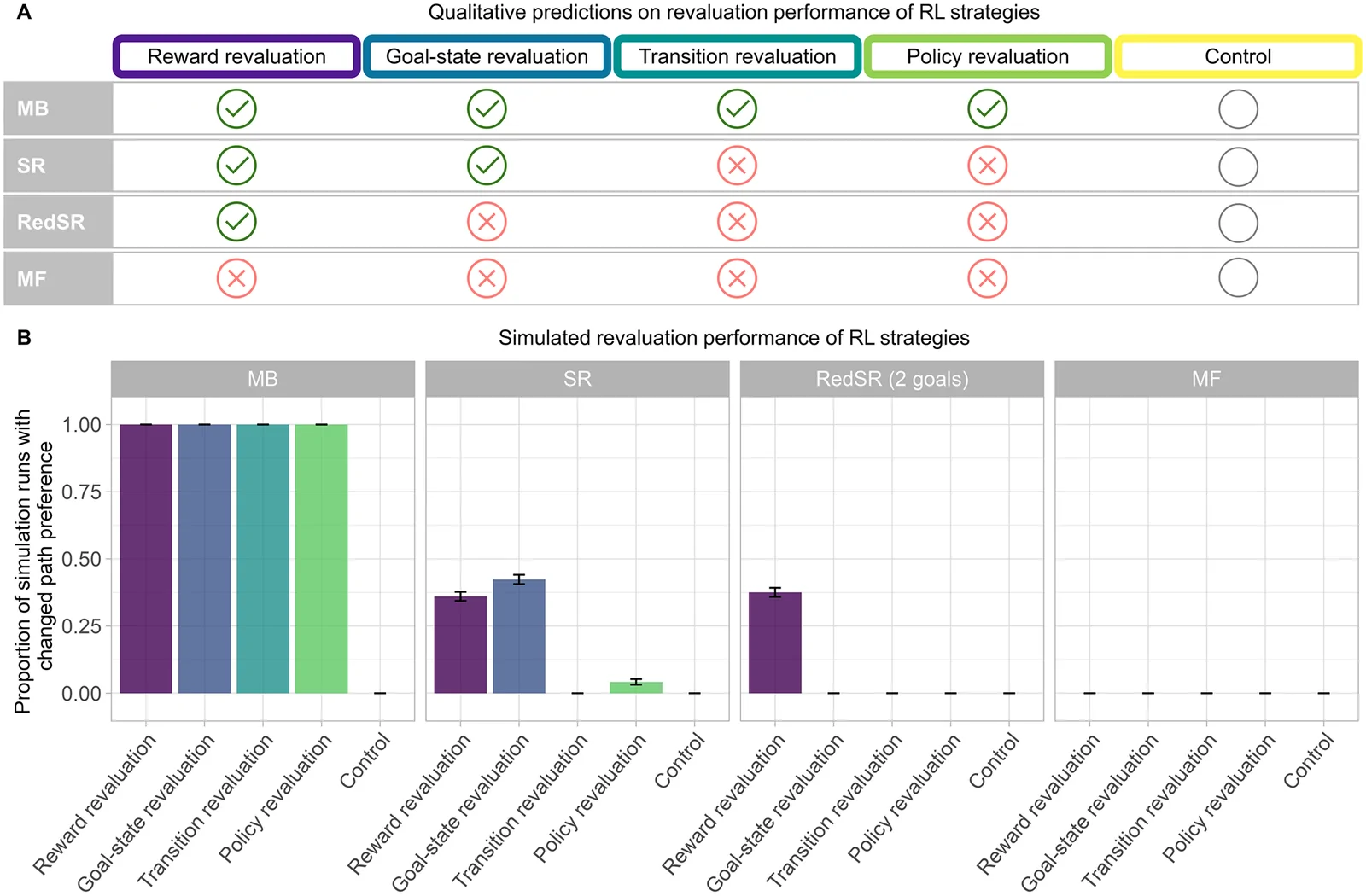
Predicted revaluation performance of *a priori* defined RL strategies. **A** Qualitative predictions on whether model-based (MB), successor representation (SR), rigid goal-based reduced successor representation (redSR), and model-free (MF) reinforcement learning (RL) algorithms (rows) can adapt to the different types of changes experienced in the conditions (columns) of the multi-stage decision-making task. Green ticks indicate that an algorithm is predicted to change its path preference in the test phase compared to the optimal path during the learning phase, whereas red crosses indicate it is not. Grey circles indicate that a change in optimal path from learning to test phase is not implied in the control condition. All RL strategies are expected to come with specific behavioral signatures: standard MB or MF RL show comparably high or low performance across revaluation conditions, respectively. One expected signature of SR agents is a lower revaluation performance in transition compared to reward and goal-state revaluation. A lower performance in policy compared to reward and goal-state revaluation is an additional predicted marker of SR RL and has been suggested to exclude that a higher revaluation performance in reward or goal-state compared to transition revaluation can be explained by an alternative MB learner with a higher learning rate for the reward compared to the transition structure [22]. Lastly, the expected signature of redSR RL which differentiates it from SR RL is a lower revaluation performance in goal-state compared to reward revaluation. **B** Simulated revaluation performance of *a priori* defined RL algorithms (MB, SR, redSR with two goal-state-action-pairs [those which led to reward during the learning phase], and MF) across task conditions, with 1000 runs per model and condition at α=0.9 and γ=0.5. Agents were considered to have an optimal path preference after learning if they had acquired a higher value for the optimal than the suboptimal state-1- and second-stage choice, respectively, and if they had chosen the optimal path in at least three out of the last five learning trials. Runs which fulfilled these criteria were taken as the base population to evaluate revaluation performance, i.e., the proportion of runs in which an agent changed its path preference on a test trial after re-learning compared to the optimal path during the learning phase. The test trial was deterministic, favoring the state-1- and second-stage action with the highest value, respectively. While, at these parameter settings, we found the expected model signatures, simulations across a range of parameter values revealed more nuanced results (Fig B in S1 Text). Bars represent means, error bars represent standard errors of the mean.

Taken together, in the multi-stage decision-making task, the hypothesized signatures of SR use are indicative of SR or redSR while the hypothesized marker of redSR use is specific to redSR with two goal-state-action-pairs. While the absence of these respective signatures does not rule out SR or redSR use in our task, their presence cannot be explained by MF, standard MB, or hybrid standard MB-MF RL [12,22].

### Low-risk drinkers likely use (reduced) random-policy SR in a non-alcohol context

#### Testing SR, redSR, standard MB, and MF contributions.

After simulating the behavior of the *a priori* defined RL strategies, we investigated their signatures in *n* = 130 low-risk drinkers performing the task in a non-alcohol context (age[*M*(*SD*)]=29.82(7.23) years; *n* male/female/other: 60/70/0; AUDIT score = 3.54(1.65); Table 1). Revaluation performance on a given test trial was predicted from task condition using logistic mixed-effects regression (for descriptive statistics, see Fig 3A, Table A in S1 Text).

**Table 1:** Sample characteristics.

	Low-risk drinkers – non-alcohol context (*n* = 130)	Low-risk drinkers - alcohol context (*n* = 130)	High-risk drinkers – non-alcohol context (*n* = 130)	High-risk drinkers – alcohol context (*n* = 130)	Group*context differences		Group differences		Context differences	
					Test statistic	*p*	Test statistic	*p*	Test statistic	*p*
**Age** (*M* [*SD*])	29.82 (7.23)	31.01 (7.41)	30.94 (7.48)	31.72 (7.23)	*t*(516)=-0.32	.747	*t*(516)=1.23	.218	*t*(516)=1.31	.191
**Sex** (*n* male/ female/ other)	60/70/0	67/63/0	86/42/2	84/46/0	χ^2^ = 22.33	<.001	χ^2^ = 17.39	<.001	χ^2^ = 2.12	.465
**Country of residence** (*n* GER/ other EU country/ UK)	53/10/67	63/0/67	14/21/95	39/0/91	χ^2^(6)=79.42	<.001	χ^2^(2)=35.84	<.001	χ^2^(2)=38.30	<.001
**Native language** (n German/ English/ other)	44/64/22	52/59/19	19/88/20	29/87/11	χ^2^(6)=31.38	<.001	χ^2^(2)=26.40	<.001	χ^2^(2)=4.37	.112
**Ethnicity** (*n* asian/ black/ white/ mixed/ other)	12/6/98/9/5	11/8/103/7/1	4/3/111/6/3	5/5/109/6/0	χ^2^ = 16.85	.170	χ^2^ = 10.07	.030	χ^2^ = 6.33	.184
**Student status** (*n* student/ no student)	47/67	39/76	37/79	34/80	χ^2^(3)=3.75	.290	χ^2^(1)=1.99	.158	χ^2^(1)=0.90	.342
**Employment status** (*n* employed/ job-seeking/ not in paid work/ other)	73/15/6/11	75/15/9/12	82/13/8/7	89/10/6/5	χ^2^(9)=7.71	.564	χ^2^(3)=6.04	.110	χ^2^(3)=0.40	.939
**AUDIT score** (M [SD])	3.54 (1.65)	4.28 (1.79)	15.34 (5.27)	13.73 (5.08)	*t*(516)=-3.47	.001	*t*(516)=24.67	<.001	*t*(516)=1.54	.123
**AUD criteria** (M [SD])	0.62 (1.15)	0.92 (1.26)	4.85 (2.47)	4.15 (2.42)	*t*(516)=-2.98	.003	*t*(516)=17.69	<.001	*t*(516)=1.25	.210
**AUD diagnosis** (*n* fulfilled/ not fulfilled)	16/114	32/98	117/13	111/19	χ^2^(3)=254.69	<.001	χ^2^(1)=247.41	<.001	χ^2^(1)=0.63	.429
**Drinking days past 3 months** (M [SD])	8.14 (7.03)^a^	12.30 (13.20)^c^	34.70 (22.79)^d^	27.69 (21.22)^f^	*t*(435)=-3.43	.001	*t*(435)=12.31	<.001	*t*(435)=1.95	.052
**Drinks per drinking day in past 3 months** (M [SD])	4.65 (5.79)^a^	4.95 (4.59)^c^	12.23 (10.35)^d^	12.67 (10.00)^f^	*t*(435)=0.09	.928	*t*(435)=7.51	<.001	*t*(435)=0.30	.764
**Binge days past 3 months** (M [SD])	1.02 (2.76)^b^	2.21 (5.89)	16.86 (16.35)^e^	15.12 (16.18)^e^	*t*(509)=-1.39	.164	*t*(509)=10.64	<.001	*t*(509)=0.80	.423
**Smoking past 3 months** (*n* yes/ no)	21/109	18/112	58/72	61/69	χ^2^(3)=58.51	<.001	χ^2^(1)=56.74	<.001	χ^2^(1)=0.00	1.00
**Cannabis use past 3 months** (*n* yes/ no)	10/120	16/114	38/92	29/101	χ^2^(3)=25.08	<.001	χ^2^(1)=20.95	<.001	χ^2^(1)=0.05	.819
**Other drug use past 3 months** (*n* yes/ no)	2/128	2/128	15/114	16/114	χ^2^(3)=22.49	<.001	χ^2^(1)=20.82	<.001	χ^2^(1)=5.8e-30	1.00
**CAS-A nonvolitional** (M [SD])	0.41 (0.95)	0.41 (0.90)	3.11 (2.40)	2.86 (2.27)	*t*(516)=-0.79	.430	*t*(516)=12.24	<.001	*t*(516)=1.05e-14	1.00
**CAS-A unaware** (M [SD])	0.33 (0.82)	0.34 (0.83)	3.19 (3.09)	2.79 (2.72)	*t*(516)=-1.09	.278	*t*(516)=10.79	<.001	*t*(516)=0.03	.977
**UPPS-P negative urgency** (M [SD])	2.05 (0.57)	2.00 (0.63)	2.56 (0.58)	2.48 (0.56)	*t*(516)=-0.37	.709	*t*(516)=7.05	<.001	*t*(516)=-0.66	.509
**UPPS-P premeditation** (M [SD])	1.78 (0.47)	1.77 (0.44)	2.07 (0.48)	1.96 (0.44)	*t*(516)=-1.18	.239	*t*(516)=5.14	<.001	*t*(516)=-0.30	.767
**UPPS-P perseverance** (M [SD])	2.03 (0.59)	1.97 (0.55)	2.22 (0.54)	2.09 (0.54)	*t*(516)=-0.74	.459	*t*(516)=2.77	.006	*t*(516)=-0.80	.422
**UPPS-P sensation seeking** (M [SD])	2.34 (0.56)	2.37 (0.61)	2.58 (0.66)	2.51 (0.54)	*t*(516)=-0.93	.353	*t*(516)=3.30	.001	*t*(516)=0.41	.685
**UPPS-P positive urgency** (M [SD])	1.62 (0.53)	1.57 (0.52)	2.14 (0.71)	2.04 (0.62)	*t*(516)=-0.57	.570	*t*(516)=6.98	<.001	*t*(516)=-0.59	.556
**OCI-R** (M [SD])	16.25 (12.18)	15.64 (12.40)	18.46 (12.36)	19.63 (13.75)	*t*(516)=0.80	.423	*t*(516)=1.40	.161	*t*(516)=-0.39	.696

*Notes*. Alcohol Use Disorder (AUD) criteria and diagnosis according to the Diagnostic and Statistical Manual of Mental Disorders (DSM)–5 [33] were assessed via a self-report questionnaire [34,35]. Drinking days, drinks per drinking day, and binge days were assessed via a Quantity Frequency Questionnaire according to the Munich Composite International Diagnostic Interview [36,37]. We removed implausible values from ‘drinking days past 3 months’ and ‘drinks per drinking day in past 3 months’ (> 7 drinking days/ week; > 91.245 drinking days/ 3 months; > 0 drinking days in past 3 months & 0 drinks per drinking day; drinks per weekday and weekend day > drinks per drinking day in past 3 months; > 50 drinks per drinking day in past 3 months) and from ‘binge days in past 3 months’ (> 7 binge days/ week; > 91.245 binge days/ 3 months). Smoking, cannabis, and other drug use were assessed using custom screening questions. If count values don’t add up to 130 per column, the difference is made up by missing data. When no degrees of freedom for χ^2^ tests are given, this indicates that *p*-values were simulated. AUDIT, Alcohol Use Disorders Identification Test [29,30]; CAS-A, Craving Automated Scale for Alcohol [38]; OCI-R, Obsessive Com*p*ulsive Inventory-Revised [39,40]; UPPS-P, Urgency-Premeditation-Perseverance-Sensation Seeking-Positive Urgency scale [41,42].

^a^Based on *n* = 122 with valid data.

^b^Based on *n* = 129 with valid data.

^c^Based on *n*=124 with valid data.

^d^Based on *n*=118 with valid data.

^e^Based on *n*=127 with valid data.

^f^Based on *n*=75 with valid data.

**Fig 3:**
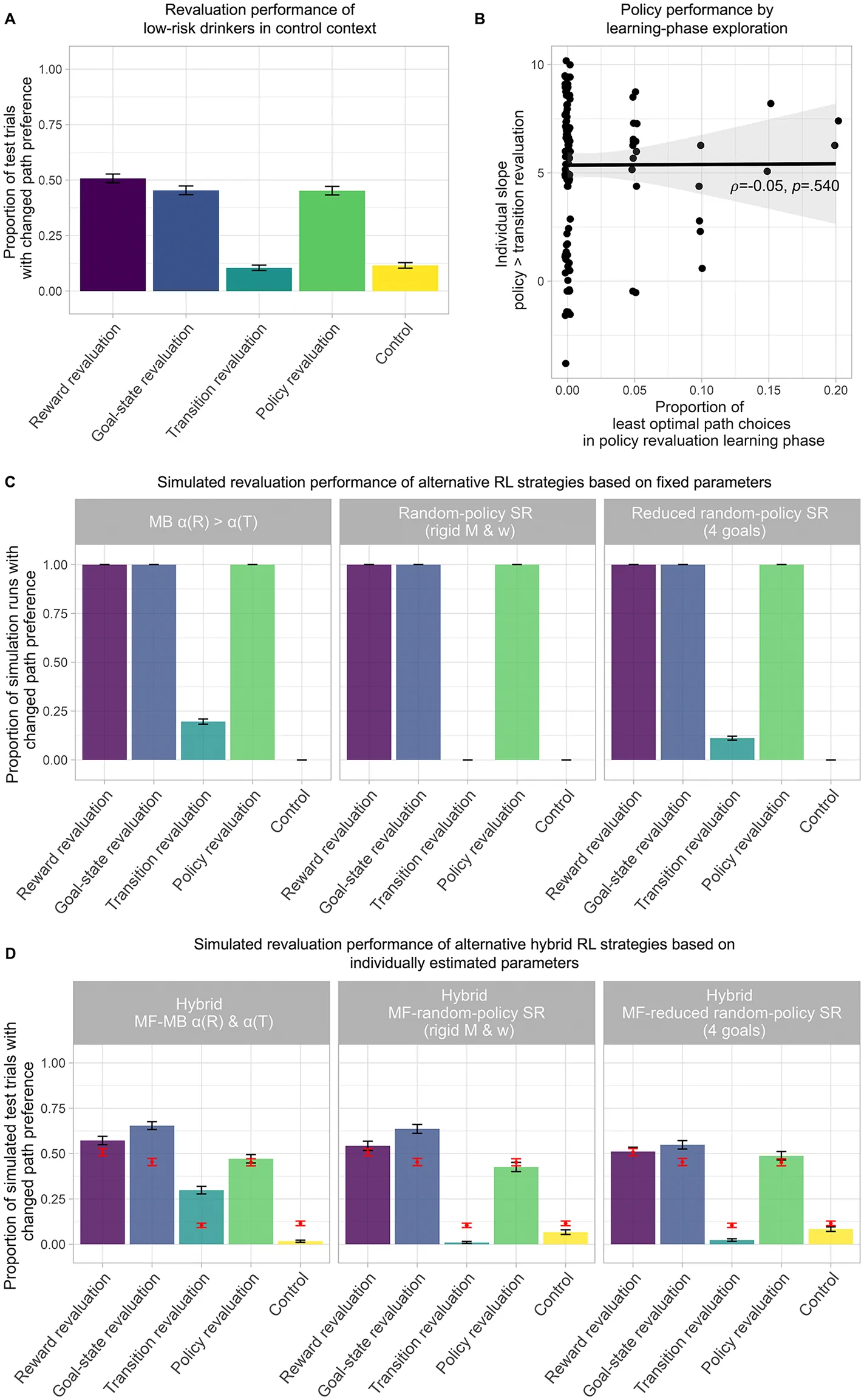
Revaluation performance in low-risk drinkers performing the multi-stage decision-making task in a non-alcohol context (*n* = 130). **A** Observed proportion of test trials per condition in which participants changed their path preference, compared to the optimal path during learning. **B** Spearman’s rank correlation between the proportion of learning trials in the policy revaluation condition on which participants chose the least optimal path (which would become optimal according to re-learning experiences) and their individual slopes for the contrast “policy> transition revaluation”. Slopes were extracted from a logistic mixed-effects regression predicting the probability of changing path preference on a test trial, compared to the optimal path during learning, from condition. **C** Simulated revaluation performance of alternative reinforcement learning (RL) algorithms (model-based [MB] algorithm with a higher learning rate for the reward structure ($\alpha_{R}$=0.9) compared to the transition structure ($\alpha_{T}$=0.3) at γ=0.5; random-policy successor representation [SR] with rigid M and w at α=0.9, γ=0.5; rigid goal-based reduced random-policy SR with four goal-state-action-pairs at α=0.9, γ=0.5 [see Methods for an explanation of goal choice]) across task conditions, with 1000 simulation runs per model and condition. Agents were considered to have an optimal path preference by the end of learning if they had acquired a higher value for the optimal than the suboptimal state-1- and second-stage choice, respectively, and if they had chosen the optimal path in at least three out of the last five learning trials. Runs which fulfilled these criteria were taken as the base population to evaluate revaluation performance, i.e., the proportion of runs in which an agent changed its path preference on a test trial after re-learning compared to the optimal path during the learning phase. The test trial was deterministic, favoring the state-1- and second-stage action with the highest value, respectively. Alternative models all produced policy revaluation performance comparable to reward and goal-state revaluation performance but higher than transition revaluation performance, combined with a high proportion of simulation runs per condition in which the optimal path preference had been acquired by the end of learning, as observed in low-risk drinkers (Figs D, E, F in S1 Text). **D** Parametric bootstrapping simulations of revaluation performance of hybrid model-free (MF) versions of alternative RL algorithms (bars and black error bars), overlaid by observed revaluation performance in *n* = 130 low-risk drinkers performing the task in a non-alcohol context (red dots and error bars). Simulations are based on individually estimated parameters (one simulation run per participant). Only simulated participants who had an optimal path preference by the end of learning (same criteria as in **C**) in all conditions were taken as the base population to evaluate revaluation performance, i.e., the proportion of test trials in which an agent changed its path preference after re-learning compared to the optimal path during the learning phase. We simulated five probabilistic test trials per participant and condition. Hybrid MF-(reduced) random-policy SR models better reproduced the contrast between reward, goal-state, and policy revaluation compared to transition revaluation, respectively, observed in humans than the hybrid MF-MB learner with $\alpha_{R}\;\&\;\alpha_{T}$. In **A**, **C**, and **D**, bars or red dots represent means, error bars represent standard errors of the mean. In **B**, the line represents a linear fit and shaded grey areas represent the 95% confidence interval.

Low-risk drinkers were significantly more likely to be successful in reward (Hypothesis 1: *OR*=1191.93, *z* = 6.24, *p* < .001) and goal-state revaluation (Hypothesis 1: *OR*=713.49, *z* = 5.75, *p* < .001) compared to transition revaluation, respectively (Table B in S1 Text). Successful revaluation could not be explained by forgetting or exploratory behavior as performance in both reward (*OR*=1548.27, *z* = 7.19, *p* < .001) and goal-state revaluation (*OR*=926.79, *z* = 6.69, *p* < .001) was significantly higher than the probability of erroneous path preference changes in the control condition (Table D in S1 Text). By contrast, this was not true for transition revaluation performance (*OR*=1.30, *z* = 0.18, *p* = .855; Table D in S1 Text).

This pattern indicates that low-risk drinkers’ behavior in a non-alcohol context cannot solely be explained by standard MB, MF, or hybrid standard MB-MF RL use [12,22]. While we did not observe the marker of redSR with two goal-state-action-pairs, i.e., lower performance in goal-state compared to reward revaluation (*OR*=0.60, *z* = -1.19, *p* = .236; Table C in S1 Text), higher success in reward and goal-state compared to transition revaluation is a signature of SR and possibly also redSR RL use in our task.

One aspect of the observed revaluation performance pattern, however, was not in line with SR or redSR RL use: performance in policy revaluation was significantly higher than in transition revaluation (*OR*=765.00, *z* = 6.00, *p* < .001; Table B in S1 Text) and higher than the probability of erroneous path preference changes in the control condition (*OR*=993.70, *z* = 6.92, *p* < .001; Table D in S1 Text), but did not differ from performance in reward (Hypothesis 2: *OR*=1.56, *z* = 1.31, *p* = .190; Table E in S1 Text) or goal-state revaluation (Hypothesis 2: *OR*=0.93, *z* = -0.21, *p* = .833; Table E in S1 Text), respectively (Fig 3A). The observed pattern cannot be explained by any of the *a priori* defined models simulated under any parameter setting when additionally considering that, per task condition, only between 2.61% and 5.88% of an initial *n* = 153 participants had a suboptimal path preference by the end of the learning phase (Methods; Figs B, C in S1 Text).

An SR strategy might produce high policy revaluation performance if the included *n* = 130 low-risk drinkers frequently chose the least optimal path before acquiring the optimal path preference by the end of the learning phase (Methods). We tested if this was the case but found no supporting evidence: the proportion of least optimal paths taken during learning was low (*M*(*SD*)=0.01(0.003)) and not correlated with individual slopes for the contrast between policy and transition revaluation performance (Fig 3B).

#### Exploring alternative learning strategies.

Given that none of the *a priori* defined models could explain the observed behavior, we explored alternative RL strategies. As suggested by Momennejad and colleagues [22], one such strategy is a MB learner with a higher learning rate for the reward ($\alpha_{R}$) compared to the transition structure ($\alpha_{T}$). When simulating this model’s behavior on the multi-stage decision-making task, we could indeed replicate the key characteristics of the revaluation performance pattern observed in low-risk drinkers based on a high proportion of simulation runs with optimal path preference by the end of the learning phase (Figs 3C, D in S1 Text).

However, SR use also allows for success in policy revaluation when making a simple modification: instead of learning $M^{\pi }$ from experience, individuals could establish $M^{random}$ under a uniform random policy [31,32]. This representation might be established during the initial forced-choice learning trials, which is supported by participants starting off with a high performance in the first free-choice trials (Fig E in S1 Text). We simulated a random-policy SR agent which learned $M^{random}$ and w in a standard MB way during the initial forced-choice learning trials and did not update them until the announcement of the re-learning phase enabled updates to both structures again (‘rigid M & w'). This model, like low-risk drinkers, showed a high proportion of simulation runs with optimal path preference by the end of the learning phase and performed comparably well in reward, goal-state, and policy revaluation while having no success in transition revaluation across a range of parameter settings (Figs 3C, F in S1 Text; for simulations of an alternative ‘rigid M’ random-policy SR model, also see Fig F in S1 Text).

We further explored whether goal-based reduced versions [23] of random-policy SR could also provide an explanation of low-risk drinkers’ behavior. Indeed, a reduced random-policy SR algorithm which established $M^{random}$ by the end of the forced-choice trials, kept it constant throughout the learning phase (whereas w was updated), and reduced it to a goal-based version $M_{red}^{random}$ assuming four goal-state-action-pairs (S7A1-S10A1, ‘4 goals’) at the end of the learning phase also produced the key characteristics of the human revaluation performance pattern, while showing a high proportion of simulation runs with optimal path preference by the end of the learning phase across a range of parameter settings (Fig 3C, G in S1 Text).

The (reduced) random-policy SR models are non-preregistered explanations which require assumptions, e.g., about the rigidity of $M^{random}$ during learning, the number of goal-states, and about how an $M^{random}$-like representation might have been acquired. While we do not claim that these exact strategies produced our data, some form of cognitive map which supports flexible adaptation to changes in policy but not in the transition structure is necessary to explain low-risk drinkers’ behavior in a non-alcohol context. To explore whether (reduced) random-policy SR models offered a better explanation of low-risk drinkers’ behavior in the non-alcohol context than a MB learner with $\alpha_{R}>\alpha_{T}$, we fit them to free-choice learning and test trial data. Results provided some quantitative evidence in favor of SR-based models: the reduced random-policy SR model (4 goals; Bayesian Information Criterion [BIC] *M ± SE* = 141.30 ± 5.75; parameter recoverability α: ρ=.87, γ: ρ=.82; Table F in S1 Text) descriptively had a higher model fit than the MB learner with separate learning rates $\alpha_{R}\;\&\;\alpha_{T}$ (BIC *M ± SE* = 148.25 ± 5.88; parameter recoverability${\;\alpha }_{R}$: ρ=.90,${\;\alpha }_{T}$: ρ=.85, γ: ρ=.89; Table F, Fig H in S1 Text). Moreover, in parametric bootstrapping simulations based on individually estimated parameters, (reduced) random-policy SR agents, compared to the MB learner with $\alpha_{R}\;\&\;\alpha_{T}$, better mimicked low-risk drinkers’ contrast between reward, goal-state, or policy and transition revaluation performance, respectively, when additionally considering that their transition revaluation performance was not significantly higher than their control performance (Fig I in S1 Text). Lastly, low-risk drinkers responded faster on transition (*M ± SE* = 528.89 ± 19.68ms) compared to reward (*M ± SE* = 576.07 ± 18.01ms; β=42.71, robust 95%CI [29.34–70.16]) and policy revaluation test trials (*M ± SE* = 596.35 ± 19.68ms; β=47.95, robust 95%CI: [26.18–66.64]; Fig J, Tables G, H in S1 Text). This is not in line with a MB agent that needs to perform computationally costly rollouts even at low $\alpha_{T}$. An SR-based learner, by contrast, might be faster in transition revaluation as this is the only condition where not only the rows of $M^{random}$ corresponding to states 1–3 but also the reward vector remains unchanged, resulting in larger value differences and less ambiguity at decision time [43].

Lastly, we explored whether MF contributions could explain why low-risk drinkers’ reward, goal-state, and policy revaluation performance was less optimal than to be expected based on random behavior in the control condition. Indeed, hybrid MF versions of alternative RL models (BIC *M ± SE* = 134.84 ± 2.51) had on average a descriptively better model fit than non-hybrid agents (BIC *M ± SE* = 162.11 ± 1.62), and the hybrid MF-reduced random-policy SR model (4 goals; BIC *M ± SE* = 129.26 ± 5.05; parameter recoverability α: ρ=.80, γ: ρ=.92, ω: ρ=.90; Table F in S1 Text) descriptively had the best fit (Fig H in S1 Text). Hybrid MF-(reduced) random-policy SR models – particularly the hybrid MF-reduced random-policy SR agent (4 goals) – were also better than the hybrid MF-MB agent with $\alpha_{R}\&\;\alpha_{T}$ (parameter recoverability ${\;\alpha }_{R}$: ρ=.57,${\;\alpha }_{T}$: ρ=.64, γ: ρ=.89, ω: ρ=.92; Table F in S1 Text) at approximating low-risk drinkers’ contrast between reward, goal-state, or policy and transition revaluation performance, respectively, in parametric bootstrapping simulations based on individual parameter estimates, when additionally considering that low-risk drinkers’ transition revaluation performance was not significantly higher than their control performance (Fig 3D, I in S1 Text). Taken together, while the data do not allow us to unequivocally distinguish between a MB agent with $\alpha_{R}>\alpha_{T}$ and (reduced) random-policy SR models, we deem hybrid MF-(reduced) random-policy SR strategies the most likely amongst the considered explanations for low-risk drinkers’ behavior in a non-alcohol context.

### High- and low-risk drinkers likely use more MF and less (reduced) random-policy SR in an alcohol context

#### Testing effects of high-risk drinking and substance-related context on SR, redSR, standard MB, and MF contributions.

We tested effects of high-risk drinking and disorder-relevant context on strategy use based on additional data from low-risk drinkers performing the multi-stage decision-making task in an alcohol context (*n* = 130; age[*M*(*SD*)]=31.01(7.41) years; *n* male/female/other: 67/63/0; AUDIT score = 4.28(1.79)) and from high-risk drinkers performing the task in either an alcohol (*n* = 130; age=31.72(7.23) years; *n* male/female/other: 84/46/0; AUDIT score = 13.73(5.08)) or non-alcohol context (*n* = 130; age=30.94(7.48) years; *n* male/female/other: 86/42/2; AUDIT score = 15.34(5.27)) (Table 1). Again, revaluation performance on a given test trial was predicted using logistic mixed-effects regression, this time from task condition, group, context, and their interactions (for descriptive statistics, see Fig 4A, Table A in S1 Text).

**Fig 4:**
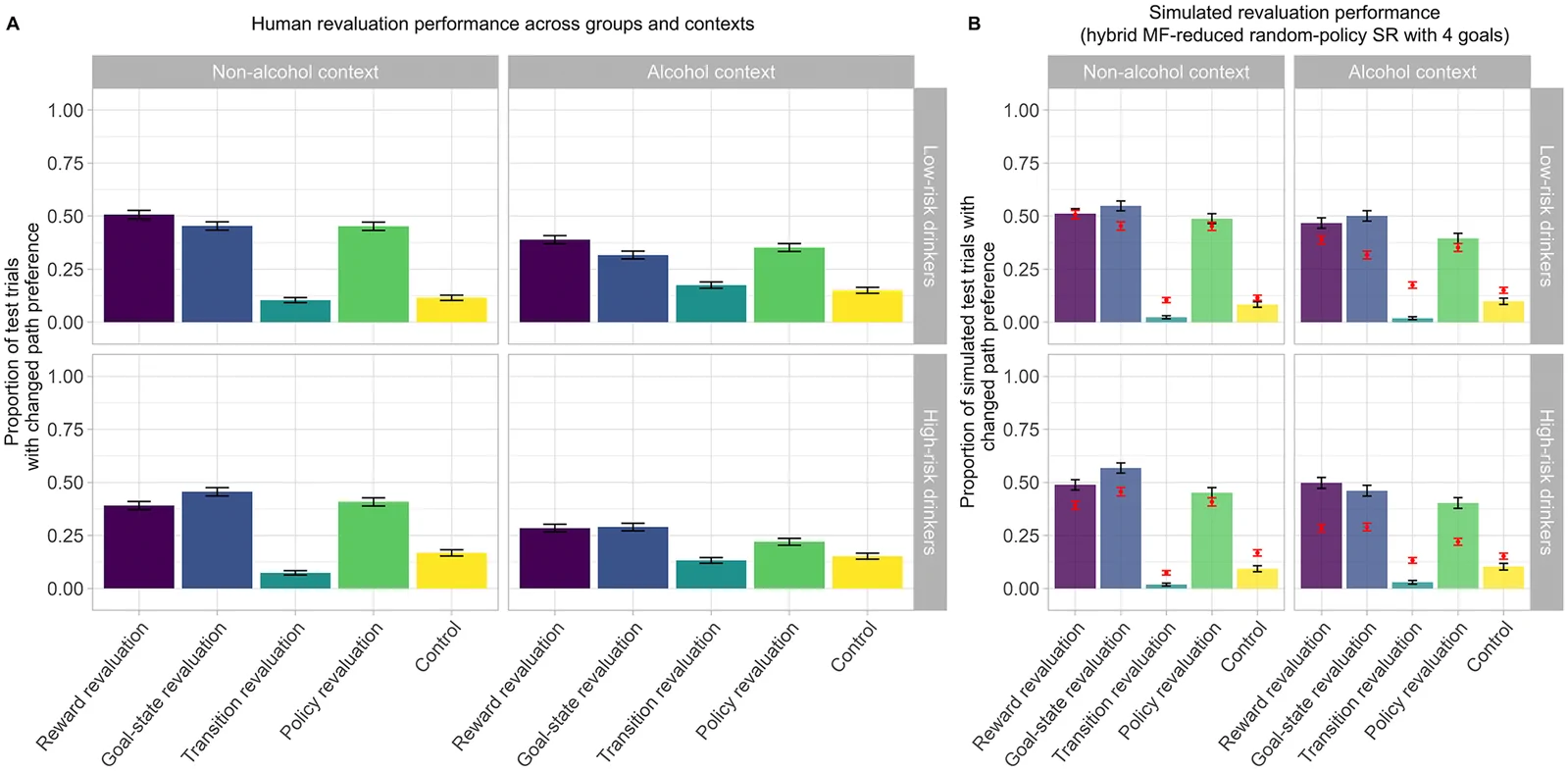
Revaluation performance in high-risk and low-risk drinkers performing the multi-stage decision-making task in an alcohol or non-alcohol context (*N* = 520, *n* = 130 per cell). **A** Observed proportion of test trials per condition, group, and context in which participants changed their path preference, compared to the optimal path during learning. **B** Parametric bootstrapping simulations of revaluation performance of the hybrid model-free (MF)-reduced random-policy successor representation (SR) model (bars and black error bars), overlaid by observed revaluation performance in *N* = 520 humans, separated by group and context (red dots and error bars). Simulations are based on individually estimated parameters (one simulation run per participant). A simulated participant was considered to have an optimal path preference after learning in a given condition if they had acquired a higher value for the optimal than the suboptimal state-1- and second-stage choice, respectively, and if they had chosen the optimal path in at least three out of the last five learning trials. Only simulated participants who fulfilled these criteria in all conditions were taken as the base population to evaluate revaluation performance, i.e., the proportion of test trials in which an agent changed its path preference after re-learning compared to the optimal path during the learning phase. We simulated five probabilistic test trials per participant and condition. Bars and red dots represent means, error bars represent standard errors of the mean.

When testing for markers of SR or redSR RL use across groups and contexts, we found that participants exhibited a higher revaluation performance in reward (*OR*=24.99, *z* = 8.37, *p* < .001) and goal-state revaluation (*OR*=23.96, *z* = 8.22, *p* < .001) compared to transition revaluation, respectively (Table I in S1 Text). Success in neither reward (*OR*=408.29, *z* = 11.28, *p* < .001) nor goal-state revaluation (*OR*=391.40, *z* = 11.34, *p* < .001) could be explained by random behavior or forgetting as it was more likely than erroneous path preference changes in the control condition averaged across groups and contexts (Table J in S1 Text). While this was also true for performance in transition revaluation across contexts and groups (*OR*=16.34, *z* = 4.54, *p* < .001; Table J in S1 Text), the effect was comparatively small.

Contrary to our predictions, when testing for effects of group and context, we found that the contrast between reward and transition revaluation was not larger in high-risk drinkers compared to low-risk drinkers averaged over contexts (Hypothesis 3: *OR*=0.50, *z* = -1.56, *p* = .118). The same was true for the contrast between goal-state and transition revaluation (*OR*=1.43, *z* = 0.80, *p* = .426; Table I in S1 Text).

Instead, both contrasts were smaller, but still significant, in the alcohol context (‘reward>transition’: *OR*=8.55, *z* = 4.95, *p* < .001; ‘goal-state>transition’: *OR*=7.02, *z* = 4.45, *p* < .001) compared to the non-alcohol context (‘reward>transition’: *OR*=73.05, *z* = 9.45, *p* < .001; ‘goal-state>transition’: *OR*=81.78, *z* = 9.59, *p* < .001) when averaged across high-risk and low-risk drinkers (‘[reward>transition]*context’: *OR*=0.12, *z* = -4.82, *p* < .001; ‘[goal-state>transition]*context’: *OR*=0.09, *z* = -5.38, *p* < .001; Table I in S1 Text). Context effects did not differ between groups (Hypothesis 4: ‘[reward>transition]*context*group’: *OR*=1.18, *z* = 0.19, *p* = .848; ‘[goal-state>transition]*context*group’: *OR*=0.85, *z* = -0.18, *p* = .857; Table I in S1 Text). Moreover, they were not accompanied by differences between contexts in the probability of choosing the optimal path per trial in the last five learning trials (Fig E, Table K in S1 Text) or in random behavior (Table J in S1 Text), rendering lower motivation or attention in bar environments unlikely alternative explanations (see S1 Text). While both high-risk and low-risk drinkers thus seemingly made use of an SR or redSR strategy, they seemed to do so less in an alcohol-related context.

Next, we tested for the signature of redSR with two goal-state-action-pairs, i.e., lower performance in goal-state compared to reward revaluation. In the alcohol context, we did not find this marker averaged across groups (*OR*=0.94, *z* = -0.21, *p* = .834), and this did not differ between groups (Hypothesis 5: *OR*=2.58, *z* = 1.90, *p* = .057; Table M in S1 Text). Across both contexts and groups, we did not find a difference between goal-state and reward revaluation performance either (*OR*=0.96, *z* = -0.22, *p* = .822; Table N in S1 Text). While an interaction with group (*OR*=2.87, *z* = 2.87, *p* = .004) revealed that, across contexts, low-risk drinkers (*OR*=0.57, *z* = -2.23, *p* = .052) performed worse in goal-state compared to reward revaluation and thus seemingly made more use of redSR with two goal-state-action-pairs than high-risk drinkers (*OR*=1.62, *z* = 1.80, *p* = .145), the contrast was not significant in any group (Table N in S1 Text). We found no effect of context (*OR*=0.73, *z* = -0.83, *p* = .406) or of the interaction between group and context (Hypothesis 6: *OR*=0.72, *z* = -0.46, *p* = .647) on the contrast between goal-state and reward revaluation performance (Table N in S1 Text). In conclusion, the data do not carry unique signatures of redSR RL use in any group or context.

While the observed higher performance in reward and goal-state compared to transition revaluation, respectively, might indicate SR or redSR RL use in our task, none of the *a priori* defined strategies could explain the full pattern across contexts and groups when additionally considering that, per task condition, only between 3.76% and 5.72% of an initial *n* = 612 participants had a suboptimal path preference by the end of the learning phase (Methods; Figs B, C in S1 Text): like in low-risk drinkers performing the task in a non-alcohol context, performance in policy revaluation was significantly higher compared to transition revaluation (*OR*=19.64, *z* = 7.88, *p* < .001; Table I in S1 Text) in the full sample. This was less pronounced in the alcohol (*OR*=5.72, *z* = 4.12, *p* < .001) compared to the non-alcohol context (*OR*=67.47, *z* = 9.55, *p* < .001; ‘[policy>transition]*context’: *OR*=0.08, *z* = -5.86, *p* < .001; Table I in S1 Text). We found no interactions between the contrast between policy and transition revaluation performance and group (*OR*=0.64, *z* = -1.08, *p* = .282) or group and context (*OR*=0.54, *z* = -0.74, *p* = .459; Table I in S1 Text), respectively. At the same time, the probability of success in policy revaluation was not significantly different from that in reward revaluation across groups and contexts (*OR*=0.79, *z* = -1.28, *p* = .202), and this did not differ significantly depending on group (*OR*=1.28, *z* = 0.67, *p* = .503), context (*OR*=0.72, *z* = -0.87, *p* = .386), or their interaction (*OR*=0.46, *z* = -1.09, *p* = .277; Table N in S1 Text).

Again, the revaluation performance pattern could not be explained by a standard SR strategy with high exploration before the included *N* = 520 participants acquired an optimal path preference by the end of the learning phase (Methods), as we found no association between the proportion of policy revaluation learning trials where the least optimal path was chosen and individual slopes for the contrast between policy and transition revaluation in the full sample (ρ=.004, *p* = .922).

#### Exploring effects of high-risk drinking and substance-related context on alternative learning strategies.

Like for low-risk drinkers performing the task in a non-alcohol context, the revaluation performance pattern observed across groups and contexts could be descriptively reproduced by (reduced) random-policy SR models or a MB learner with $\alpha_{R}>\alpha_{T}$ simulated across a range of parameter values on our task (Figs 3C, D, F, G in S1 Text). For all alternative RL models, this was the case at parameter settings that concomitantly resulted in a high proportion of simulation runs with an optimal path preference by the end of learning (Figs D, F, G in S1 Text).

When fitting the alternative RL models and their hybrid MF versions to all participants’ free-choice learning and test trial data, we again found some quantitative evidence in favor of SR-based models: the reduced random-policy SR model (4 goals; BIC *M ± SE =* 138.06 ± 2.74; parameter recoverability α: ρ=.85; γ: ρ=.84; Table F in S1 Text) had a descriptively better fit than each of the other non-hybrid agents (Fig H in S1 Text). Moreover, hybrid MF models (BIC *M ± SE =* 131.17 ± 1.18) on average descriptively fit the data better than non-hybrid RL models (BIC *M ± SE =* 155.85 ± 0.81), and the hybrid MF-reduced random-policy SR model (4 goals; BIC *M ± SE =* 125.79 ± 2.35; parameter recoverability α: ρ=.77, γ: ρ=.91, ω: ρ=.87; Table F in S1 Text) had a descriptively higher fit than the hybrid MF-MB agent with $\alpha_{R}\;\&{\;\alpha }_{T}$ (BIC *M ± SE =* 130.94 ± 2.32; parameter recoverability${\;\alpha }_{R}$: ρ=.57,${\;\alpha }_{T}$: ρ=.66, γ: ρ=.90, ω: ρ=.94; Table F, Fig H in S1 Text). In parametric bootstrapping simulations, (reduced) random-policy SR RL models and their hybrid MF versions were better at approximating the full sample’s contrast between reward, goal-state, or policy and transition revaluation performance, respectively, in combination with a transition revaluation performance not meaningfully higher than their control performance, compared to the (hybrid MF-)MB learner with $\alpha_{R}\;\&\;\alpha_{T}$, respectively (Figs 4B, K in S1 Text). Like in low-risk drinkers performing the non-alcohol task version, response times were lower in transition (*M ± SE* = 551.97 ± 8.95ms) compared to reward (*M ± SE* = 599.10 ± 8.92ms; β=47.13, *t*(520.00)=2.99, *p* = .003), goal-state (*M ± SE* = 612.91 ± 9.46ms; β=60.94, *t*(520.00)=3.46, *p* = .001) and policy revaluation (*M ± SE* = 615.92 ± 9.60ms; β=63.95, *t*(520.64)=3.95, *p* < .001), respectively (Fig J, Tables G, O in S1 Text), providing further evidence for the use of an SR-based over an alternative MB strategy [43]. While contributions of a MB learner with $\alpha_{R}\;\&\;\alpha_{T}$ cannot be excluded, results thus suggest that a hybrid MF-(reduced) random-policy SR-like representation shapes behavior not only in low-risk drinkers performing the task in a non-alcohol context but also in high-risk drinkers and alcohol-related contexts.

The lower (reduced) random-policy SR contributions in the alcohol context across groups suggested by weaker contrasts between reward, goal-state, or policy revaluation and transition revaluation performance, respectively, were reflected in the ω-parameter of hybrid MF-(reduced) random-policy SR agents (Table P in S1 Text): ω was lower in the alcohol (hybrid MF-reduced random-policy model [4 goals]: *M ± SE* = 0.63 ± 0.02) compared to the non-alcohol context (*M ± SE* = 0.69 ± 0.01; β=-0.06, *t*(516)=-2.86, *p* = .013; Table Q in S1 Text), indicating a stronger influence of MF value estimates on choice behavior. While a lower learning rate might also explain lower reward, goal-state, and policy revaluation performance in the alcohol con*t*ext, we found no supporting evidence (Table R in S1 Text).

In conclusion, high-risk drinking was not associated with more pronounced markers of (reduced) random-policy SR use which could potentially also reflect a MB strategy with separate learning rates for the reward and transition structure in our task. This is in line with exploratory results showing no positive associations between use of these strategies and self-report measures of, e.g., drinking frequency or habitual alcohol intake (Fig L in S1 Text). Instead, participants made less use of what is likely a (reduced) random-policy SR strategy and seemed to rely more strongly on MF value estimates in substance-related contexts. Importantly, results were similar when measuring revaluation performance via a more deliberate judgement of value as we could replicate key effects from test phase analyses in rating phase data (Fig M, Tables S-AA in S1 Text). For further discussion of potential alternative explanations and confounding factors, see S1 Text.

## Discussion

The habit theory of addiction predicts that the transition from recreational drinking to substance use disorders is associated with enhanced habitual and reduced goal-directed behavior [4,6,7], but evidence in humans is inconsistent [2,9–11]. We suspected two reasons for this: first, high-risk drinkers might make increased use of SR [18,19] and redSR [23] RL, two strategies with subtle habit-like characteristics [19,22] which are not detectable in common paradigms used to assess habitual behavior in humans [1,12], and second, those habit paradigms are usually conducted in disorder-irrelevant contexts. Here, we thus set out to investigate whether SR and redSR use is increased in high-risk drinkers making disorder-relevant choices.

In a first step, we aimed to replicate previous findings suggesting that humans use SR RL [22,24,44,45]. With an extended version of the multi-stage decision-making task by Momennejad et al. [22] in a more naturalistic non-alcohol-related context, we found that the behavior of 130 low-risk drinkers could not be explained by a standard MB, MF, or a hybrid standard MB-MF strategy alone: participants exhibited higher reward than transition revaluation performance – a key signature of SR use [21,22] which, according to simulations, could also indicate redSR use in our task [23]. Unlike in [22], our sample was no more likely to adapt to transition revaluation than to erroneously change path preference. This is not in line with a hybrid SR-MB strategy like SR-Dyna [21,22], where MB contributions should allow some success in transition revaluation, nor with a recently suggested probabilistic SR [46] or a default representation updated via matrix inversion identities [31]. In light of previous findings indicating that humans rely more on a MB strategy when SR leads to incorrect value estimation [25] and MB is thus most needed [22], the low MB engagement in transition revaluation seems surprising. However, participants did not experience that SR-derived value estimates for state-1 actions were inaccurate in our task (unlike in [25]), and the larger state space compared to [22] enhanced the difference in computational costs between MB and SR RL and possibly cognitive load [47]. One can thus speculate that a cost-benefit arbitration would have favored SR over MB use in the multi-stage decision-making task.

While the signature of SR or redSR use was more pronounced in our data compared to [22], neither of these policy-dependent strategies can explain why low-risk drinkers did not perform worse in policy than in reward and goal-state revaluation in a non-alcohol-related context. Adaptation to distal new rewards introduced in a state which has rarely been visited in the past suggests some form of policy-independent representation. As we concomitantly saw low performance in transition revaluation, this could be the one-step transition matrix T used in MB RL only if T is learned at a lower rate than the environment’s reward structure [22]. Based on model-fitting and response time findings, however, we deem a random-policy SR-like map $M^{random}$ which represents all paths through the environment with equal expected occupancies [31,32] – and is then potentially dimension-reduced to future occupancies of goal-state-action-pairs only ($M_{red}^{random}$) [23] – a more plausible explanation. The introduction of forced-choice trials, unlike in [21], enforced initial equal experience of all four paths starting from state 1 and might have favored the formation of $M^{random}$. While a single experience per path would not be enough for an SR strategy to learn an $M^{random}$-like representation using temporal difference (TD)(0) updates, participants might have used a computationally expensive standard MB strategy at the beginning and then transformed T to $M^{random}$ to benefit from cheaper computations throughout the learning phase. Such a strategy could reproduce key characteristics of the observed revaluation pattern when simulated, particularly when combined with MF contributions explaining why reward, goal-state, and policy revaluation performance were lower than expected based on random behavior or forgetting as measured by erroneous path preference changes in the control condition alone. Such a strategy would also align with evidence that humans and rats use a more MB strategy in earlier task trials before shifting towards SR use [48]. Another way to learn an $M^{random}$-like representation assuming no transformation between MB and SR representations could be an SR algorithm using an eligibility trace, i.e., a TD(λ) update rule [49,50], allowing for backpropagation of state occupancies to all predecessor states along a path after just a single experience. On a neural level, acquisition of an $M^{random}$-like representation might be based on Behavioral Timescale Synaptic Plasticity (BTSP) [51–53] and Spike-Timing Dependent Plasticity (STDP) [54]. BTSP enables a representation of each state in terms of sequences in which it was encountered [51,55], and the dot product of two states’ representations, similar to one element in $M^{random}$, quantifies how many sequences both states are part of [51]. As sequences of up to six seconds can be learned in one shot using BTSP [52], but one trial in our task took longer (Fig A in S1 Text), additional STDP during online replay [54] could serve to form a representation like $M^{random}$ based on just four trials covering the full environment [51–53]. Neither MB nor TD learning would thus be necessary to rapidly establish an SR-like cognitive map [54] which, if established during forced-choice trials, would look $M^{random}$-like in our task. If and how $M^{random}$ is then dimension-reduced needs to be further investigated, specifically by inducing a type of goal-state revaluation where new rewards appear after previous goal-states (Methods) and by assessing, e.g., neurophysiological and memory retrieval measures to determine which states are considered goal-states. Furthermore, confirmatory tests of the contributions of non-preregistered RL strategies are needed, with a focus on dissociating (reduced) random-policy SR from a MB learner with different learning rates for the reward and transition structure. Specifically, this could be achieved by introducing probabilistic transitions and rewards to further facilitate model identifiability or by increasing the number of re-learning trials: a MB learner would improve in transition revaluation in that case, whereas SR-based learners would not.

Supposing a hybrid MF-(reduced) random-policy SR-like strategy explains the observed revaluation performance pattern in low-risk drinkers performing the task in non-alcohol contexts, it contributed to behavior also in high-risk drinkers and alcohol-related contexts. Different than expected, the markers of SR (or, on our task, redSR) use, which we consider as likely indicative of (reduced) random-policy SR given the full revaluation pattern, were not more pronounced in high-risk compared to low-risk drinkers across contexts, nor was a unique marker of redSR use more pronounced in high-risk drinkers when exclusively considering the alcohol context. This suggests that the inconsistent evidence for increased habitual responding in human addiction [2,9,10] is not due to a shift from MB to SR-based strategies which cannot be detected in classic habit paradigms [1,12]. Instead, while (reduced) random-policy SR use leads to habit-like inflexibility when distal transitions in the environment change, it is generally an adaptive and flexible strategy. (Reduced) random-policy SR-like models – and also MB agents with a higher learning rate for the reward than the transition structure – thus do not seem to be a suitable formalization of dysfunctional, inflexible behavior. From the perspective of the habit theory of addiction [4,6,7], it is unsurprising that individuals with more risky and automated drinking behavior (Table 1) do not make more use of such strategies. Goal-based dimension reduction, when applied to a random-policy SR, allows for flexible, policy-independent adaptation to changes in the environment’s reward structure and describes the observed choice behavior even better than use of non-reduced random-policy SR, particularly when combined with MF contributions. To further investigate the role of dimension reduction in high-risk drinking, future work should explore the effect of resistance to goal-reaching, either by experimentally inducing it or by focusing on individuals wanting to limit alcohol consumption [23].

Different than expected, individuals relied less on what is likely a (reduced) random-policy SR-like strategy and instead seemed to rely more on MF learning in alcohol-related contexts. Alternative explanations for these context effects, such as lower motivation or lower attentional engagement in the alcohol context, can largely be ruled out as we ensured comparable learning in both contexts, as participants did not behave more randomly in the alcohol-related context, and as each context included five different environments, minimizing effects of specific stimuli (see S1 Text). Our finding thus suggests that, in individuals with prior alcohol experience, (reduced) random-policy SR-like strategies – or potentially MB strategies with a higher learning rate for the reward than the transition structure – are selectively downregulated in alcohol-related contexts. It has previously been suggested that habitual and goal-directed contributions to behavior depend on each strategy’s certainty in a context-dependent manner [56]. As ethanol has detrimental effects on performance in tasks sensitive to hippocampal impairment [57], such as episodic memory formation [58] and spatial learning [59,60], and alters the function of place cells in the hippocampus [61,62], a structure which seems to encode cognitive maps with strong similarities to the SR [63–66], non-alcohol-naïve participants might have learned that cognitive-map-based value estimates are often inaccurate in alcohol-related contexts and thus default to computationally cheaper MF behavior. Indeed, contextual cues associated with alcohol intoxication impaired goal-directed behavior in rodents [67] (but see [68]). Future research in individuals with AUD versus no prior drinking experience should further elucidate context-dependent strategy contributions and the factors that mediate them, such as context-induced affective states or craving [69], e.g. by using different control contexts to isolate the relevant characteristics of disorder-related contexts. Moreover, it is worth further investigating ethanol but also alcohol context effects on the accuracy of neural cognitive map representations.

In conclusion, we replicated a key signature of SR-based strategy use [22] in a naturalistic task. However, low-risk drinkers’ behavior in non-alcohol-related contexts did not align with canonical (red)SR’s policy dependence. While their behavior may be in line with contributions of a MB strategy with a lower learning rate for the environment’s transition than reward structure, a hybrid MF-(reduced) random-policy SR strategy seems to offer a more plausible explanation. To our knowledge, this is the first study to test associations between use of SR-based RL and psychopathology. We expected a shift away from a MB towards a (red)SR strategy in high-risk drinkers, particularly in alcohol-related contexts, but instead found a shift away from what is likely a (reduced) random-policy SR-like strategy towards what is likely more habitual MF RL in alcohol-related contexts, independent of high-risk versus low-risk drinking status. Our results suggest that the inconsistent evidence for increased habitual behavior in human addiction cannot be explained by enhanced use of SR-based strategies as more nuanced substrates of habit [22]. Instead, random-policy SR-based RL, even its goal-based dimension-reduced variant, is a resource-rational strategy enabling flexible behavior under a range of circumstances, rather than a computational proxy for pathologically inflexible behavior in addiction. That substance-related contexts seemed to shift strategy use away from this RL strategy underscores the importance of considering context when testing hypotheses about altered cognitive map representations in mental health conditions.

## Materials and methods

### Ethics statement

All participants gave written informed consent and were compensated with £12 for 1.5h of time, plus an optional bonus payment (see multi-stage decision-making paradigm). The study was approved by the ethics committee at Dresden University of Technology, Germany (no. SR-EK-578122022).

### Participants and procedures

Participants between 18 and 45 years old who did not generally abstain from or wish to abstain from alcohol at the time of study participation and had normal or corrected-to-normal vision were recruited via Prolific.

Participants first answered basic demographic questions and completed the AUDIT questionnaire [29,30] which was used to determine group allocation. High-risk drinkers had an AUDIT score >7, whereas low-risk drinkers had an AUDIT score <8 and indicated that they had never been in individual therapy for alcohol use. Participants then completed the multi-stage sequential decision-making task before filling in the AUDIT questionnaire a second time. We additionally assessed AUD criteria according to the Diagnostic and Statistical Manual of Mental Disorders (DSM)-5 [33] using a self-assessment questionnaire [34,35], drinking days, drinks per drinking day, and binge drinking days in the past 3 months, respectively, via a Quantity Frequency Questionnaire according to the Munich Composite International Diagnostic Interview [36,37], automated drinking behavior via the Craving Automated Scale for Alcohol (CAS-A) [38], tobacco, cannabis, and other drug use in the past three months, respectively, via custom screening questions, impulsivity via the Urgency-Premeditation-Perseverance-Sensation Seeking-Positive Urgency (UPPS-P) scale [41,42], and obsessive compulsive symptoms via the Obsessive Compulsive Inventory-Revised (OCI-R) [39,40].

To achieve the preregistered sample size of *N* = 420 after an estimated exclusion rate of 25%, we initially recruited *N* = 560 individuals with complete task and questionnaire data. After application of exclusion criteria (see below), we continued to recruit until we reached a full balancing of task condition order and an equal sample size in each of the four subsamples, resulting in recruitment of a total of *N* = 666 participants with complete data (*n* = 167 low-risk drinkers in non-alcohol context; *n* = 158 low-risk drinkers in alcohol context; *n* = 175 high-risk drinkers in non-alcohol context; *n* = 166 high-risk drinkers in alcohol context). Out of these, *N* = 54 (8.11%; *n* = 14 low-risk drinkers in non-alcohol context; *n* = 6 low-risk drinkers in alcohol context; *n* = 21 high-risk drinkers in non-alcohol context; *n* = 13 high-risk drinkers in alcohol context) were excluded as their answers in the pre- and post-task AUDIT questionnaires indicated a different group allocation. To ensure that all participants had learned which path was optimal by the end of the learning phase, we further excluded *N* = 92 individuals (15.03%) who had chosen the optimal path in less than three out of the last five learning trials in any task condition (*n* = 23 low-risk drinkers in the non-alcohol context; *n* = 22 low-risk drinkers in the alcohol context; *n* = 24 high-risk drinkers in the non-alcohol context; *n* = 23 high-risk drinkers in the alcohol context). The percentage of participants who fulfilled this exclusion criterion per task condition was as follows: 5.72% in the reward revaluation condition, 3.76% in the goal-state revaluation condition, 5.07% in the transition revaluation condition, 4.25% in the policy revaluation condition, and 4.58% in the control condition. After applying this exclusion criterion, our final sample consisted of *N* = 520 individuals (*n* = 130 per subsample; see Table 1 for sample characteristics).

### Multi-stage decision-making paradigm

The multi-stage decision-making task adapted from Momennejad et al. [22] consisted of five task conditions (reward revaluation, goal-state revaluation, transition revaluation, policy revaluation, control condition) encompassing four phases (learning, re-learning, test, rating) each (Fig 1A). The order of conditions was balanced according to a Latin square design across participants. Participants performed the task in either an alcohol or a non-alcohol context, with each condition taking place in a separate environment. Which condition took place in which environment was approximately balanced across participants (Fig P in S1 Text). Images representing states in the different environments were generated using OpenAI’s DALL-E (Figs 1B, Fig A in S1 Text; for stimulus material, see publicly available code at github.com/agschlagenhauf/SR_in_AUD_behav.git). Which state was represented by which image in each environment was randomized within each stage of the decision tree.

In the non-alcohol context, participants navigated through five apartment environments consisting of 10 rooms (states) each to collect as much hidden cash as possible (Fig 1). The cover story was that every Friday, they would go out for dinner with friends to a restaurant which accepted no card payments. They regularly forgot to withdraw cash during the week but luckily made it a habit to deposit some cash for emergencies in certain spots in their apartment. How much cash they deposited where, however, they could not remember. Shortly before going to the restaurant on Fridays, they would thus have to search the usual spots for cash. Changes in apartment environments between conditions were explained by staying in a vacation home or moving from time to time.

In the alcohol context, participants navigated through five bar environments consisting of 10 rooms (states) each to collect as many glasses of their preferred alcoholic drink as possible (Fig 1). Participants could choose their preferred drink (beer, wine, long drinks/cocktails) before starting the task. Here, the cover story was that every Saturday after a stressful week at work, they would go for drinks in their favorite bar. As the bar was popular, some rooms would usually be booked for private events, and bar counters in some rooms would serve their favorite drink only in small quantities or be out of it entirely. While they would never know in what room they would get what number of glasses, they would try to collect as many as possible. Changes in bar environments between conditions were explained by having a new favorite bar from time to time.

In both contexts, participants were instructed that how much reward they received in a room would not depend on how they got there, that what had been learned in one environment would not be valid in others, that they could take a self-paced break between environments, and that they had to choose an action as quickly as possible in each room. If they exceeded the time limit (2s in learning and re-learning trial states with one available action, 3s in learning trial states with two available actions, 15s in test trial states; Fig A in S1 Text), they would start the current trial from the beginning. Left actions were selected using the ‘F’ key, right actions using the ‘J’ key, and single actions using the ‘space’ key. As an incentive, participants were informed that the person who collected the most cash or glasses across the entire task would get a bonus payment (non-alcohol context) or a beverage store voucher (alcohol context) worth £/€50, and that participants in the second to fourth place would receive bonus payments or vouchers worth £/€15 each. At the end of the study, participants in the alcohol context task were informed that, for ethical reasons, the most successful individuals would not receive beverage store vouchers, but instead bonus payments. We determined the four winners for each of the four subsamples (16 winners in total) and paid bonus payments via Prolific after completion of data collection.

Before starting the task, all participants read instructions, saw a floor plan of the environment, performed a 10-trial training version of the task, and completed a quiz. The floor plan did not show the underlying task structure depicted in Fig 1, but instead showed states on the same stage, e.g., states 4–6, as adjacent numbered squares without doors to not imply a specific transition structure. The training consisted of four forced-choice and six free-choice trials starting from state 1 and was performed in an environment not used on the actual task to avoid carry-over effects. The quiz encompassed eight true/false questions about the instructions with no time limit. If any of the questions was answered incorrectly, participants had to restart instructions from the beginning.

Only once all quiz questions had been answered correctly, participants started the actual task. In the initial 24-trial learning phase per condition, all trials started from state 1 of the 10-state environment. States 1–3 contained two actions (doors) each and states 4–10 contained a single action (searching a spot in an apartment room or ordering at a bar counter), respectively. A path through the environment was thus determined by first- and second-stage actions. Rewards were delivered in fourth-stage states and implied that one path starting from state 1 was optimal, i.e., led to the highest reward. Which state-1-action initiated the optimal path was randomized between conditions (i.e., rewards in states 7 and 9 were swapped), with three conditions having an optimal path starting with the left state-1-action and two conditions having an optimal path starting with the right state-1-action. The first four learning trials were forced-choice, i.e., only one door in states 1–3 was available and highlighted and the other one was greyed out. In the following 20 free-choice trials, both doors in states 1–3 were available. Before starting the task, participants had been instructed to memorize during forced-choice trials where they would receive the most reward and then to collect as much reward as possible afterwards.

In the following nine-trial re-learning phase, participants experienced changes to the reward structure (in reward, goal-state, policy revaluation and the control condition) or transition structure (in transition revaluation) of the environment (for a detailed description, see Fig 1). Changes in all revaluation conditions implied a change in optimal path compared to what had been experienced during the learning phase, whereas changes in the control condition did not. All re-learning trials started from third-stage states (3 trials from state 4, 5, and 6, each) and were presented in randomized order. Before beginning the task, participants had been instructed that they would sometimes start in later rooms than the first one, that sometimes things would change in an environment, and that they would have to notice these changes and act accordingly to collect as much reward as possible. Immediately before each condition’s re-learning phase, participants were only informed that a few weeks had passed since they went out for dinner (non-alcohol context) or had been to their favorite pub (alcohol context) and that now, they would save some time and start from later rooms.

In the five-trial test phase per condition, participants then started from state 1 again. Immediately before this phase, they were instructed that they would not see where their actions would take them and how much reward they would receive, but that they should nevertheless choose the path that they believed would lead them to the highest amount of reward and that their decisions would be considered for the bonus payments or vouchers. The test phase served to derive the primary measure of revaluation performance, i.e., to assess whether, on a given test trial, a participant chose a different path from the path which had been optimal during the learning phase (for an exact definition of this primary outcome measure, see “Mixed-effects regression analyses of revaluation performance”).

In the final rating phase per condition, participants were presented two times with state 1, with a different action being highlighted on each presentation. On each of the two screens, they were asked how much cash or how many glasses of alcohol the highlighted action would lead to and could indicate their answer on a sliding scale ranging from 0 = ‘no cash/ glasses of alcohol’ to 100 = ‘lots of cash/ glasses of alcohol’ using the left and right arrow keys. Numbers were not shown on screen, and answers were confirmed using the ‘Enter’ key. This phase allowed to derive a secondary measure of revaluation performance, i.e., the difference in rating scores between state-1 actions (for an exact definition of this secondary outcome measure, see “Mixed-effects regression analyses of revaluation performance”).

### Computational models

We compared the task behavior of four *a priori* defined RL algorithms – an SR, redSR, standard MB, and MF learner – and of several alternative RL models – a MB algorithm with different learning rates for R and T, random-policy SR models, reduced random-policy SR algorithms, and respective hybrid MF versions – to that of human low-risk and high-risk drinkers.

#### Environment and policy.

The task environment consisted of 10 states and 13 state-action pairs in total (two actions from states 1–3, respectively; Fig 1). Q-values derived from all non-hybrid models were transformed into action probabilities using a standard softmax policy π:


$$\begin{array}{c} \pi \left(a|s\right)=\frac{e^{{(Q}_{sa}-\text{max}(Q_{sa^{\prime }}))}}{\sum_{a^{\prime }}e^{{(Q}_{sa^{\prime }}\;-\text{max}(Q_{sa^{\prime }}))}}, \end{array}$$ (1)

where a' denotes all actions available in state s. Here, the temperature parameter is implicitly set to 1, constraining the parameter space and standardizing choice stochasticity across RL models. This ensures that differences in simulated revaluation performance or model fit reflect differences in value representation rather than models choosing more or less deterministically.

#### SR algorithm.

The SR algorithm [18,19,21] represents the environment via a state-action-state-action successor matrix $M^{\pi }$, where each state-action-pair sa (in row sa) is represented by the expected cumulative future discounted number of visits to each successor state-action-pair $s^{\prime }a^{\prime }$ (in column $s^{\prime }a^{\prime }$), when starting from sa and following the softmax policy π:


$$\begin{array}{c} M_{sa,s^{\prime }a^{\prime }}^{\pi }=\;E\left[\sum_{t=\text{0}}^{\infty }\gamma^{t}I\left({sa}_{t}=s^{\prime }a^{\prime })\right|{sa}_{\text{0}}=sa\right] \end{array}$$ (2)

Here, I(·) equals 1 if its argument is true, i.e., if the agent transitioned to s'a' starting from sa in the current time step t, and 0 otherwise, and γ is a time discount factor between 0 and 1, with higher values indicating less discounting.

The successor matrix $M^{\pi }$ is learned from experience using (TD)(0) updates. Specifically, after transitioning from a state-action-pair sa to a successor state-action-pair s'a', the row of $M^{\pi }$ corresponding to sa is updated as follows:


$$\begin{array}{c} M_{sa,:}^{\pi }\leftarrow M_{sa,:}^{\pi }+\alpha_{SR}\left[{\text{I}}_{sa}+\gamma M_{s^{\prime }a^{\prime },:}^{\pi }-M_{sa,:}^{\pi }\right] \end{array}$$ (3)

Here, $\alpha_{SR}$ is an SR learning rate between 0 and 1, and ${\text{I}}_{sa}$ is a vector containing zeros at all indices except for a 1 at the index corresponding to the state-action-pair sa which the agent transitioned from.

To derive a value estimate for action a from state s, the SR algorithm uses the row of $M^{\pi }$ corresponding to sa as a feature vector and linearly weights it by the elements in a vector w:


$$\begin{array}{c} Q_{sa}^{\pi }=\;\sum_{s^{\prime }a^{\prime }}M_{sa,s^{\prime }a^{\prime }}^{\pi }w_{s^{\prime }a^{\prime }} \end{array}$$ (4)

The weight vector w is itself learned from experience via TD-learning optimized for linear function approximation instead of delta rule learning of immediate punctate rewards [19,21,23] and gradually approaches the one-step reward received in each state-action pair sa. Specifically, after a transition sa→s'a', all elements i of w are updated as follows:


$$\begin{array}{c} w_{i}\leftarrow w_{i}+\alpha_{TD}\delta_{TD}M_{sa,i}^{\pi }, \end{array}$$ (5)

where $\alpha_{TD}$ is a learning rate parameter, the feature vector $M_{sa,:}^{\pi }$ is scaled by $M_{sa,:}^{\pi }{\times M_{sa,:}^{\pi }}^{T}$ to allow for interpretation of $\alpha_{TD}$ as a proportional step-size [21], and ${\;\delta }_{TD}$ is the TD RPE:


$$\begin{array}{c} \delta_{TD}=r_{sa}+\gamma Q_{s^{\prime }a^{\prime }}-Q_{sa} \end{array}$$ (6)

Here, $r_{sa}$ is the immediate reward observed after performing action a from state s.

In our implementation of the SR algorithm, $M^{\pi }$ was initialized as a 13 × 13 identity matrix and w and Q as all-zero vectors, respectively. Upon entering a state s, the agent decided for an action a using Equation (1) (in simulations) or observed an action a (in model fitting) and, upon receiving the corresponding reward, first updated the weight vector w using Equation (5) and then updated Q-values using Equation (4) based on the updated w and the next predicted (in simulations) or observed (in model fitting) action s'a'. After transitioning to the next state s', the agent then updated the successor matrix $M^{\pi }$ using Equation (3).

#### RedSR algorithm.

The redSR algorithm [23] operates like the SR learner, with several exceptions: before entering the re-learning phase, $M^{\pi }$ is reduced to $M_{red}^{\pi }$ which contains columns only for goal-state-action-pairs. In the simplest case, goal-state-action-pairs are those which led to reward during learning (‘2 goals’ in Figs 2B and 2C in S1 Text). The weight vector w is also reduced to $w_{red}$ containing elements only for goal-state-action-pairs. During re-learning, $M_{red}^{\pi }$ is rigid, i.e., not updated, whereas $w_{red}$ continues to be updated according to Equation (5).

The fact that all elements of $w_{red}$ are updated after each transition, and that the update is based on the dimension-reduced, inaccurate $M_{red}^{\pi }$, is what has previously been reported to cause redSR’s addiction-like characteristics, such as sustained positive RPEs upon reaching goal-states under a resistant policy [23]. These characteristics would not be observed if redSR would use delta-rule learning of immediate punctate rewards [21,23]. However, it is also what causes redSR to succeed in goal-state revaluation under certain parameter settings (Fig A in S1 Text). Specifically, in goal-state revaluation, the RPE $\delta_{TD}$ caused by the new reward updates all elements i of $w_{red}$ which correspond to old goal-states that have previously been reached from the new goal-state (old goal-states in column i are > 0 in $M_{sa,i}^{\pi }$, see Equation (5)). While $w_{red}$ does not contain an element representing the new goal-state directly, the elements i of $w_{red}$ corresponding to old goal-states are thus inflated and affect value estimation according to Equation (4). This does not apply when the newly introduced reward does not lie on a path to a previous goal-state, as is the case in policy revaluation. An alternative version of goal-state revaluation where the new goal is introduced after a previous one would avoid redSR’s unexpected success in goal-state revaluation. Here, we decided against this alternative version to rule out that failure to adapt to goal-state revaluation might simply be due to scale dependency, i.e., due to individuals representing the environment only up until previous goal-states.

Moreover, as there is no definitive answer to the question which dimensions are relevant in dimension reduction [26] or, more specifically, which state-action-pairs are perceived as goal-state-action-pairs in our task, we implemented two alternative versions of redSR which considered additional goal-state-action-pairs beyond the two state-action-pairs leading to reward during learning: 1) the final state-action-pair in the sequence (s10a1; ‘3 goals’ in Fig C in S1 Text) and 2) s10a1 plus the third state-action-pair s7a1, s8a1, or s9a1 on the same stage as the two state-action-pairs containing reward during learning (‘4 goals’ in Fig C in S1 Text). The final state-action-pair in the sequence is temporally close to an event boundary. Event boundaries might be associated with increased attention [70] which, in turn, has been suggested to guide dimension-reduction in RL [26]. Moreover, activity in hippocampus has been shown to be sensitive to event boundaries (even though hippocampus activity peaks after, not before a boundary) [71]. States on the reward-containing stage might be more salient as participants might have meta-learned that reward will likely be delivered after the fourth action in a trial across conditions [72,73]. However, as we do not explicitly test which states are perceived as salient (see Discussion), the results only allow conclusions about whether a certain selection of goal-states is a more or less likely explanation of revaluation performance in humans.

#### Standard MB algorithm.

The MB learner [74] represents the environment by a one-step state-action-state transition matrix T, where each element $T_{sa,s^{\prime }}\;$equals the probability of reaching state s' (in columns s') in the immediate next timestep t when performing action a in state s (in row sa):


$$\begin{array}{c} T_{sa,s^{\prime }}=\;P\left({s_{t+\text{1}}=s}^{\prime }|s_{t}=s,\;a_{t}=a\right) \end{array}$$ (7)

To derive a maximum likelihood estimate of T, we construct a count matrix $T^{count}$ which contains the cumulative number of observations per transition and is updated after each transition sa→s' according to the following rule:


$$\begin{array}{c} T_{sa,s^{\prime }}^{count}\leftarrow T_{sa,s^{\prime }}^{count}+\text{1} \end{array}$$ (8)

To derive T, elements $T_{sa,s^{\prime }}^{count}$ are then normalized to ensure that each row $T_{sa,:}$ sums to 1:


$$\begin{array}{c} T_{sa,s^{\prime }}=\;\frac{T_{sa,s^{\prime }}^{count}}{\sum_{s^{\prime \prime }}T_{sa,s^{\prime \prime }}^{count}}, \end{array}$$ (9)

where s'' denotes all states in the environment. The probability $T_{sa,s^{\prime }}\;$thus equals the number of times that transition $sa\;s^{\prime }$ was observed, divided by the number of times any transition sa s'' was observed [74].

To derive Q-value estimates for a state-action-pair sa, the MB learner iterates the Bellman equation, which combines T with knowledge about the environment’s reward structure R, until convergence:


$$\begin{array}{c} Q_{sa}^{\pi }=\;R_{sa}+\gamma \sum_{s^{\prime }}T_{sa,s^{\prime }}\sum_{a^{\prime }}\pi (a^{\prime }|s^{\prime })Q_{s^{\prime }a^{\prime }}^{\pi } \end{array}$$ (10)

One-step rewards $R_{sa}$ are themselves learned from experience using a delta rule on the immediate reward $r_{sa}$ received following action a from states:


$$\begin{array}{c} R_{sa}\leftarrow R_{sa}+\alpha_{R}\delta_{R}, \end{array}$$ (11)

where $\alpha_{R}$ is a learning rate parameter and $\delta_{R}$ is defined as:


$$\begin{array}{c} \delta_{R}=r_{sa}-R_{sa} \end{array}$$ (12)

$T^{count}$and T were initialized as 13 × 10 matrices of all zeros, respectively, and R and Q as all-zero vectors. In our implementation, the agent decided for an action a using Equation (1) (in simulations) or observed an action a (in model fitting) upon entering a state s and then updated the reward vector R using Equation (11) based on the reward just observed, before re-estimating all Q-values using Equation (10). After transitioning to s', T was updated using Equations (8) and (9).

#### MF algorithm.

The MF learner [74] has no representation of the environment. Instead, it caches Q-values directly and updates them after each transition sa→s'a' using TD learning:


$$\begin{array}{c} Q_{sa}\leftarrow Q_{sa}+\alpha_{TD}\delta_{TD}, \end{array}$$ (13)

where $\delta_{TD}$ is defined as in Equation (6). We initialized Q as an all-zero vector.

#### Alternative MB algorithm with $\alpha_{R}$ and$\alpha_{T}$.

The MB algorithm with a learning rate $\alpha_{R}$ for the reward structure and a learning rate $\alpha_{T}$ for the transition structure [75, 76] operates like the MB learner, with the exception that T is learned via state prediction error $\delta_{T}$-based updates. Specifically, following a transition sa→s', the row of T corresponding to sa is updated as follows:


$$\begin{array}{c} T_{sa,:}\leftarrow T_{sa,:}+\alpha_{T}\delta_{T,} \end{array}$$ (14)

where $\alpha_{T}$ is a learning rate parameter and $\delta_{T}$ is defined as:


$$\begin{array}{c} \delta_{T}={\text{I}}_{s^{\prime }}-T_{sa,:} \end{array}$$ (15)

${\text{I}}_{s^{\prime }}$is a vector of zeros except for a 1 at the index corresponding to the state s' which the agent transitioned to.

We initialized T as a 13 × 10 matrix with a small non-zero prior for all possible transitions, i.e., all elements except the ones in the last row corresponding to transitions from S10A1 had a value of 1/ *n* states = 0.1. Elements in the last row of T were initialized by zero. R and Q were initialized as all-zero vectors. Upon entering a state s, the agent decided for an action a using Equation (1) (in simulations) or observed an action a (in model fitting) and, after observing the corresponding reward, updated the reward vector R using Equation (11) before updating Q-values using Equation (10). After transitioning to s', T was updated using Equation (14).

#### Random-policy SR algorithm.

The random-policy SR algorithm learned $M^{random}$ during the initial forced-choice trials. While this can happen in several ways (see Discussion), here, we implemented the standard MB learner described above to learn T using Equations (8) and (9) and R using Equation (11). After trial 4, i.e., before entering the first free-choice learning trial, the state-action-state transition matrix T is first transformed to a state-action-state-action transition matrix under a random policy$T^{random}$:


$$\begin{array}{c} T_{sa,s^{\prime }a^{\prime }}=\;T_{sa,s^{\prime }}/\sum s^{\prime }a^{\prime \prime }, \end{array}$$ (16)

with $\sum s^{\prime }a^{\prime \prime }$ being the number of available actions in state s'.

Each element $T_{sa,s^{\prime }a^{\prime }}\;$equals the probability of performing action a' in state s' (in columns $s\text{'}a^{\prime }$) in the immediate next timestep t when performing action a in state s (in row sa):


$$\begin{array}{c} T_{sa,s^{\prime }a^{\prime }}=\;P\left({s_{t+\text{1}}=s}^{\prime },\;{a_{t+\text{1}}=a}^{\prime }|s_{t}=s,\;a_{t}=a\right) \end{array}$$ (17)

$T^{random}$is then transformed to $M^{random}$ using matrix inversion [21]:


$$\begin{array}{c} M^{random}=\;{\left(I-{\gamma T}^{random}\right)}^{-\text{1}} \end{array}$$ (18)

The weight vector w used by the SR learner is set to equal R learned by the MB algorithm.

Starting with the first free-choice learning trial, the random-policy SR algorithm then uses Equation (4) to update value estimates. However, $M^{random}$ and w are rigid (‘rigid M & w’), i.e., not updated, during the learning phase. Only during re-learning trials, the random-policy SR algorithm operates exactly like the SR algorithm described above, using Equation (3) to update $M^{random}$ and Equation (5) to update w.

As $M^{random}$ is the defining feature of random-policy SR, we implemented an alternative version of the random-policy SR algorithm where w is updated throughout free-choice learning phase trials (‘rigid M’).

#### Reduced random-policy SR algorithm.

The reduced random-policy SR algorithm operated like the alternative random-policy SR algorithm where w (Equation (5)), but not $M^{random}$, is updated throughout free-choice learning phase trials (‘rigid M’), with the exception that, before entering the re-learning phase, $M^{random}$ was reduced to $M_{red}^{random}$ containing columns only for goal-state-action-pairs, and w was reduced to $w_{red}$ containing elements only for goal-state-action-pairs. During re-learning, $M_{red}^{random}$ remains rigid, whereas $w_{red}$ is continually updated using Equation (5).

Like for redSR, we simulated three versions of reduced random-policy SR with different numbers of goal-state-action-pairs (‘2 goals’, ‘3 goals’, and ‘4 goals’).

#### Hybrid MF models.

The hybrid MF-(reduced) random-policy SR models and the hybrid MF-MB model with $\alpha_{R}\;\&\;\alpha_{T}$ updated MF as well as non-MF value estimates in parallel according to agent-specific update rules. To derive hybrid choice probabilities, MF and non-MF Q-values were first transformed to model-specific choice probabilities using Equation (1) before then being weighted as follows:


$$\begin{array}{c} {\pi \left(a|s\right)}_{hybrid}={\omega *\pi \left(a|s\right)}_{non-MF}+(\text{1}-\omega )*{\pi \left(a|s\right)}_{MF} \end{array}$$ (19)

Higher values in ω thus indicate a stronger influence of non-MF (i.e., of [reduced] random-policy SR or MB) value estimates on choice behavior, whereas lower ω-values indicate stronger MF contributions, similar to previous formalizations of hybrid MB-MF models [12].

#### Overview of free parameters per model.

Except for the MB learner with $\alpha_{R}\;\&\;\alpha_{T}$, all non-hybrid models had two free parameters: a learning rate α and a time discount factor γ. For SR, redSR, random-policy SR, and reduced random-policy SR algorithms, this means that $\alpha_{TD}$ and $\alpha_{SR}$ were assumed to be equal. The MB learner with $\alpha_{R}\;\&\;\alpha_{T}$ ($\alpha_{R}$, $\alpha_{T}$, γ) as well as the hybrid MF-(reduced) random-policy SR models (α, γ, ω) had three free parameters, respectively, whereas the hybrid MF-MB model with $\alpha_{R}\;\&\;\alpha_{T}$ had four free parameters ($\alpha_{R}$, $\alpha_{T}$, γ, ω).

### Simulation procedure

We simulated task behavior of all non-hybrid models based on a range of fixed parameter values. Specifically, we performed simulations at all possible combinations of parameter values [0.1, 0.3, 0.5, 0.7, 0.9] for α and γ, respectively. Only the MB model with $\alpha_{R}\;\&\;\alpha_{T}$ was simulated at $\alpha_{R}$=0.9 and $\alpha_{T}$=[0.1, 0.3, 0.5]. At each parameter setting, we conducted 1000 simulation runs per model and condition. In each condition, agents performed four forced-choice learning trials in random order, 20 free-choice learning trials, and nine re-learning trials. Parameters were assumed to stay constant across task phases. After the learning and re-learning phase, respectively, we conducted one deterministic test trial (no softmax) to check whether agents had acquired a higher value for the optimal first-state- and second-stage-choice. Models were considered to have acquired an optimal path preference if they were successful in the learning test trial and if the optimal path additionally had been chosen in at least three out of the last five learning trials (same criterion as applied to human data). Only successful learning runs were considered to determine revaluation performance. In revaluation conditions, agents were considered to have successfully changed their path preference if they had acquired a higher value for the now optimal first state and second-stage choice in the deterministic re-learning test trial. For example, if an agent had learned successfully in 500 runs in a certain condition, a successful change in path preference after re-learning in 250 of these runs would indicate a revaluation performance of 50%. In the control condition, models were considered to have made an erroneous change in path preference if any but the still optimal path after re-learning was chosen in the deterministic test trial.

### Model fitting procedure

We estimated individual parameters per participant for all RL models using maximum likelihood estimation. Specifically, the negative log likelihood of observed state-1-choices and second-stage-choices on the free-choice learning trials and test trials was jointly minimized across all task conditions per participant using the limited-memory Broyden–Fletcher–Goldfarb–Shanno optimization algorithm with bounds (L-BFGS-B) [77]. Choices on forced-choice learning trials and re-learning trials were used to perform model-specific value updates, but not to maximize choice log likelihood. On test trials, value estimates were not updated. All free parameters were constrained to a range between 0.001 and 1 and initialized at 0.5.

#### Model comparison.

To compare fit between models, we calculated Akaike’s Information Criterion (AIC) and the Bayesian Information Criterion (BIC) per participant and model:


$$\begin{array}{c} AIC=\text{2}*k-\text{2}*LL \end{array}$$ (20)

$$\begin{array}{c} BIC=k*\text{ln}(n)-\text{2}*\text{LL}, \end{array}$$ (21)

where k is the number of free parameters for a given model and n is the number of state-1-choices and second-stage-choices considered to calculate the log likelihood (LL) per participant (n=250 [2 choices * 25 trials * 5 conditions]). Individual AICs and BICs were then averaged across participants.

#### Parametric bootstrapping simulations.

To assess the goodness-of-fit per model, we simulated choice data for *N* = 520 participants based on their individually estimated parameters and visually compared simulated to observed choices. For each participant and condition, we performed a single simulation run including four forced-choice learning trials in random order, 20 free-choice learning trials, nine re-learning trials, and five probabilistic test trials using the softmax choice rule (Equation (1)). After the learning phase, we conducted one deterministic test trial (no softmax) to check whether agents had acquired a higher value for the optimal first-state- and second-stage-choice. Simulated participants were considered to have acquired an optimal path preference after learning in a given condition if they were successful in the learning test trial and if the optimal path additionally had been chosen in at least three out of the last five learning trials (same criterion as applied to human data). Only simulated participants who had acquired an optimal path preference by the end of the learning phase of all task conditions were considered to evaluate simulated revaluation performance. In revaluation conditions, a successful change in path preference on a simulated probabilistic test trial was defined as choice of the now optimal first-state- and second-stage-action. In the control condition, an erroneous path preference change was defined as choosing any but the still optimal path after re-learning on a probabilistic test trial.

#### Parameter recoverability.

To assess parameter recoverability per model, the single simulated full-task choice dataset per participant based on their individually estimated parameters was fitted using the same model that had produced the dataset. We then calculated Spearman’s rank correlation between parameters estimated based on observed choices and recovered parameter values across participants.

#### Testing for parameter differences.

For hybrid (reduced) random-policy SR models, we explored the effects of sum-contrast-coded group (-0.5 = low-risk, 0.5 = high-risk), context (-0.5 = non-alcohol, 0.5 = alcohol), and their interaction on estimates of ω and α using linear regression in *R* version 4.4.1 [78]. P-values were Bonferroni-corrected for three comparisons as we tested parameter estimate differences based on three different RL models.

### Mixed-effects regression analyses of revaluation performance

Mixed-effects regression models were implemented using the *lme4* package [79] in *R* version 4.4.1 [78]. We included the maximum random effect structure supported by Likelihood Ratio Tests. Significance tests and confidence intervals for fixed effects were based on Wald degrees of freedom for logistic mixed-effects models, and on Satterthwaite’s degrees of freedom for linear-mixed-effects models. We performed *post hoc* tests for significant interactions using the *emmeans* package [80] and report *p*-values Bonferroni-corrected for the number of comparisons within a sub-dataset (e.g., within each task context). Analyses were based on complete trials only, i.e., trials were excluded if a participant exceeded a state’s response time limit.

#### Hypothesis tests.

To test the preregistered hypotheses, we used logistic mixed-effects regression models predicting revaluation performance on a given test trial, i.e., the probability of choosing a different path from the one which had been optimal during the learning phase. For the four revaluation conditions, this variable was coded as 1 if the path chosen on a test trial was optimal according to what had been experienced during re-learning, and as 0 otherwise. Conversely, for the control condition, this variable was coded as 1 if any but the optimal path according to what had been experienced during learning and re-learning was chosen erroneously, and as 0 if the optimal path was chosen.

To test hypotheses 1 and 2 in low-risk drinkers performing the task in the non-alcohol context, we included a fixed effect for dummy-coded condition with transition revaluation (hypothesis 1) or policy revaluation (hypothesis 2) as reference category, respectively. The model included a by-subject random intercept and random slope for condition to control for individual differences in revaluation performance in the reference condition as well as in contrasts between conditions of interest and the reference condition. We used the following *lme4* code:

change_path ~ condition + (1 + condition | participantID)

To test hypotheses 3, 4, and 6 in the full sample, we added fixed effects for group and context as well as all possible two-way and three-way interactions and kept the same random effect structure as above. Condition was dummy-coded with transition revaluation (hypothesis 3 and 4) and reward revaluation (hypothesis 6) as reference category, respectively. Group (-0.5 = low-risk, 0.5 = high-risk) and context (-0.5 = non-alcohol, 0.5 = alcohol) were sum-contrast-coded in all analyses. We used the following *lme4* code:

change_path ~ group*context*condition + (1 + condition | participantID)

To test hypothesis 5 in low-risk and high-risk drinkers performing the task in an alcohol context, revaluation performance on a given test trial was predicted from fixed effects for dummy-coded condition with reward revaluation as a reference category, group, and their interaction. We again used the same random-effects structure as above. The *lme4* code used thus was as follows:

change_path ~ group*condition + (1 + condition | participantID)

In low-risk drinkers performing the non-alcohol task version and in the full sample, we repeated models with the control condition as a reference category to exclude random behavior or forgetting as reasons for revaluation success and with reward or policy revaluation as reference categories, respectively, to support our argument.

Individual slopes for the different contrasts of the condition predictor were derived by adding the participant’s random slope extracted using *lme4*’s *ranef()* function to individually applicable fixed effects. Correlations amongst slopes or with other measures were quantified via Spearman’s rank correlation coefficient.

#### Exploratory analyses of response times.

In low-risk drinkers performing the task in a non-alcohol context, we explored the effect of dummy-coded task condition on response times in test phase trials (states 1–3) using linear mixed-effects regression. The *lme4* code was as follows:

response_time ~ condition + (1 + condition | participantID)

In the full sample, we additionally assessed effects of sum-contrast-coded group and context as well as of all possible interactions using the following *lme4* code:

response_time ~ group*context*condition + (1 + condition | participantID)

#### Exploratory analyses of rating phase data.

We explored performance in the rating phase using linear mixed-effects regression. To derive the rating score difference per participant as a dependent variable, we subtracted the rating of the state-1-action which was optimal during learning from the rating of the state-1-action which was suboptimal during learning. Positive rating score differences thus indicate a preference for the state-1-action which was suboptimal during learning and optimal (in revaluation conditions) or still suboptimal (in the control condition) according to what had been experienced during re-learning.

Analogously to hypotheses 1 and 2, we explored condition effects in low-risk drinkers performing the non-alcohol task version using the following *lme4* code:

diff_rating_score ~ condition + (1 | participantID)

Analogously to hypotheses 3, 4, and 6, we explored group, context, and condition effects in the full sample using *lme4* code as follows:

diff_rating_score ~ group*context*condition + (1 | participantID)

Analogously to hypothesis 5, we explored group and condition effects in the alcohol context using the following *lme4* code:

diff_rating_score ~ group*condition + (1 | participantID)

Models did not include a by-subject random slope for condition, as each participant had only one rating score difference value. Predictors were contrast-coded as described for confirmatory logistic mixed-effects regression models.

## Supporting information

S1 Text Supporting Information for “The successor representation in high-risk drinking and alcohol-related contexts” including all supplementary Tables and Figures. (DOCX)
