# Supplementary material for: The successor representation in high-risk drinking and alcohol-related contexts
Source: PLoS Comput Biol. 2026 Oct 21;22(10):e1014873. doi: 10.1371/journal.pcbi.1014873 (PMC13716319; doi:10.1371/journal.pcbi.1014873)
Supplement: Supplementary file 1 [file pcbi.1014873.s001.docx]

Supporting Information for

**The successor representation in high-risk drinking and alcohol-related contexts**

Milena P. M. Musial^1,2,3,4,*^, Sam Hall-McMaster^5^, Kanji Shimomura^6,7,8^, Ayaka Kato^9,10,11^, Erik L. Bode^1,2,3^, Clarissa C. Grundmann^12^, Claudia Ebrahimi^1^, Kenji Morita^6,13^, Samuel J. Gershman^5^, Tanja Endrass^12^, & Florian Schlagenhauf^1,3,4^

^1^Charité – Universitätsmedizin Berlin, corporate member of Freie Universität Berlin and Humboldt-Universität zu Berlin, Department of Psychiatry and Neurosciences | CCM, NeuroCure Clinical Research Center, Berlin, Germany

^2^Humboldt-Universität zu Berlin, Faculty of Life Sciences, Department of Psychology, Berlin, Germany

^3^Charité – Universitätsmedizin Berlin, corporate member of Freie Universität Berlin and Humboldt-Universität zu Berlin, Einstein Center for Neurosciences Berlin, Berlin, Germany

^4^Charité – Universitätsmedizin Berlin, corporate member of Freie Universität Berlin and Humboldt-Universität zu Berlin, Bernstein Center for Computational Neuroscience, Berlin, Germany

^5^Department of Psychology and Center for Brain Science, Harvard University, Cambridge, Massachusetts, United States of America

^6^Graduate School of Education, The University of Tokyo, Tokyo, Japan

^7^Japan Society for the Promotion of Science, Tokyo, Japan

^8^Department of Information Medicine, National Institute of Neuroscience, National Center of Neurology and Psychiatry, Tokyo, Japan

^9^Department of Psychiatry, Icahn School of Medicine at Mount Sinai, New York, New York, United States of America

^10^Department of Psychiatry and Behavioral Sciences, Graduate School of Medical and Dental Sciences, Institute of Science Tokyo, Tokyo, Japan

^11^Well-Vitality Visionary Initiative, Institute of Science Tokyo, Tokyo, Japan

^12^Institute of Clinical Psychology and Psychotherapy, Technische Universität Dresden, Dresden, Germany

^13^International Research Center for Neurointelligence (WPI-IRCN), The University of Tokyo, Tokyo, Japan

*milena.musial@charite.de

**Figure A. Sequences from an exemplary trial in the multi-stage decision-making task. A** Upon entering a two-choice state (states 1, 2, 3), available options were highlighted in yellow. In forced-choice trials with only one available action, the unavailable one was marked in grey. As soon as an action was selected, it was highlighted in pink and the unavailable action’s highlight disappeared before the next state was shown. **B** Single-choice states (states 4-10) followed a similar structure, with the exception that only one action was highlighted in yellow and the reward contained in some states was shown immediately after action selection on top of the action highlighted in pink. All state images were shown at a screen position corresponding to their location in the decision-tree as shown in Figure 1 (‘middle’ for states 1, 5, 8, and 10; ‘slightly left’ for state 2; ‘slightly right’ for state 3; ‘outer left’ for states 4 and 7; ‘outer right’ for states 6 and 9). Position did not change in the transition revaluation condition. While stimuli shown here are from the alcohol context task version, trials followed the same structure in the non-alcohol context. Stimulus material, also the stimuli depicted in panels **A** and **B** of this figure, was generated using *DALL-E 3* by *OpenAI.* For full stimulus material, see publicly available code ([github.com/agschlagenhauf/SR_in_AUD_behav.git](https://github.com/agschlagenhauf/SR_in_AUD_behav.git)).

**Figure B. Simulated learning and revaluation performance of *a priori* defined learning strategies across a range of parameter settings**. We ran 1000 simulation runs per model and condition. Learning performance was defined as the proportion of simulation runs with an optimal path preference by the end of learning. Agents were considered to have an optimal path preference by the end of learning if they had acquired a higher value for the optimal than the suboptimal state-1- and second-stage-choice, respectively, and if they had chosen the optimal path in at least three out of the last five learning trials. Runs which fulfilled these criteria were taken as the base population to evaluate revaluation performance, i.e. the proportion of runs in which an agent changed its path preference on a test trial after re-learning compared to the optimal path during the learning phase (see Methods). The test trial was deterministic, favoring the state-1- and second-stage-action with the highest value, respectively. For model details and simulation procedure, see Methods. Bars represent means, error bars represent standard errors of the mean. Note **(1)** the low proportion of simulation runs with optimal path preference by the end of the learning phase for SR and redSR agents, particularly in transition and policy revaluation conditions, which stands in contrast to the low percentage of human participants with suboptimal path preference per condition. This model behavior can be explained as follows: in transition and policy revaluation conditions, the path segment S5A1 🡪 S8A1 🡪 S10A1 is traversed twice during the initial forced-choice learning trials. As SR and redSR agents use the temporal difference (TD)(0) update rule, the prediction of S8A1 is backpropagated exactly one additional step compared to those of S7A1 and S9A1, i.e., either to S2A2 or S3A1 depending on the random trial order. Consequently, SR and redSR agents incorrectly assign a higher value to either S2A2 or S3A1 compared to S3A2, respectively, by the end of forced-choice learning trials, meaning that one of these paths will likely be chosen over the optimal path in subsequent free-choice learning trials. In reward, goal-state, and control conditions, the path segment S5A1 🡪 S8A1 🡪 S10A1 is also visited twice during forced-choice learning trials, but S8A1 is not associated with a reward, negating any effect of repeated traversals on value estimates. Instead, the prediction of S7A1 containing the second-largest reward is backpropagated one step by the end of forced-choice learning trials, same as the prediction of S9A1 containing the largest reward. While SR and redSR agents still sometimes explore the path containing the second-largest reward in initial free-choice learning trials, leading to further backpropagation of the S7A1 prediction and thus to its predecessor state-action pairs acquiring higher values than those of S9A1, this behavior is less likely than in transition and policy revaluation conditions. Together, this explains why the proportion of simulation runs with optimal path preference by the end of the learning phase is low for SR and redSR agents in reward, goal-state, and control conditions, but even lower in transition and policy revaluation conditions. Also note **(2)** the SR and redSR agents’ low revaluation performance in reward and goal-state revaluation under weak time discounting (high 𝛾-values). This is because value differences between paths are generally more accentuated under weak time discounting, leading to more exploitative behavior during learning. In simulation runs which are considered as base population to evaluate revaluation performance, the agents have thus almost always chosen the optimal path during learning. Consequently, the predictive representations (rows of $M^{\pi}$) of S1A1 and S2A1 will contain elements near zero in the columns corresponding to S4A1 and S7A1 where the new rewards are introduced. The new rewards thus have no or only a minimal effect on value estimates. Lastly, note **(3)** that the SR learner has some success in policy revaluation when learning rate is high and strong time discounting is applied (low $\gamma$-values). This can be explained by more explorative behavior during the learning phase at low $\gamma$. As policy revaluation performance is, however, not on the same level as reward and goal-state revaluation performance and occurs in conjunction with a low proportion of simulation runs with optimal path preference by the end of the learning phase, we do not consider SR a plausible explanation of the revaluation performance pattern observed in humans, where, per task condition, only between 3.76% and 5.72% of participants were excluded due to a suboptimal path preference by the end of the learning phase (Methods). MB, model-based; MF, model-free; redSR, rigid goal-based reduced SR with two goal-state-action-pairs; SR, successor representation; $\alpha$, learning rate used to update the reward vector $R$ (MB), the weight vector $w$ (SR, redSR), and value estimates $Q$ (MF), as well as the successor matrix $M$ (SR, redSR).

**Figure C. Simulated learning and revaluation performance of a rigid goal-based reduced successor representation (redSR) strategy with differing numbers of goal-state-action-pairs across a range of parameter settings**. We ran 1000 simulation runs per model and condition. Learning performance was defined as the proportion of simulation runs with an optimal path preference by the end of learning. Agents were considered to have an optimal path preference by the end of learning if they had acquired a higher value for the optimal than the suboptimal state-1- and second-stage-choice, respectively, and if they had chosen the optimal path in at least three out of the last five learning trials. Runs which fulfilled these criteria were taken as the base population to evaluate revaluation performance, i.e. the proportion of runs in which an agent changed its path preference on a test trial after re-learning compared to the optimal path during the learning phase (see Methods). The test trial was deterministic, favoring the state-1- and second-stage-action with the highest value, respectively. Goal-state-action-pairs were either only the pairs that led to a reward during learning (2 goals), additionally the action from final state 10 (3 goals), or additionally the third state-action-pair on the same stage as the reward-associated state-action-pairs during learning (4 goals). For model details and simulation procedure, see Methods. Bars represent means, error bars represent standard errors of the mean. For an explanation of **(1)** the low proportion of simulation runs with optimal path preference by the end of the learning phase of all agents, particularly in transition and policy revaluation conditions, standing in contrast to what we observed in humans, as well as for an explanation of **(2)** the low reward and goal-state revaluation performance under weak time discounting (high $\gamma$-values), see legend of Figure B. Moreover, note that **(3)** the redSR learner with 4 goal-state-action-pairs has some success in policy revaluation when strong time discounting is applied (low $\gamma$-values). This is because learning phase behavior is generally more explorative under strong time discounting. However, as policy revaluation performance is not on the same level as reward and goal-state revaluation performance or, when it is, it occurs in conjunction with a low proportion of simulation runs with optimal path preference by the end of the learning phase, we do not consider redSR with 4 goal-state-action-pairs a plausible explanation of the revaluation performance pattern observed in humans. This is because, per task condition, only between 3.76% and 5.72% of participants were excluded due to a suboptimal path preference by the end of the learning phase (Methods). The redSR agents with 2 or 3 goal-state-action-pairs show no success in policy revaluation as neither the state-action-pair newly associated with a reward nor any successor state-action-pair is considered a goal.

**Table A.** Revaluation performance per group, context, and condition of the multi-stage decision-making task.

| **Group** | **Context** | **Condition** | ***M*** | ***SE*** | **95% CI lower limit** | **95% CI upper limit** |
| --- | --- | --- | --- | --- | --- | --- |
| Low-risk drinkers | Non-alcohol | Reward revaluation | 0.51 | 0.02 | 0.47 | 0.55 |
| Low-risk drinkers | Non-alcohol | Goal-state revaluation | 0.45 | 0.02 | 0.42 | 0.49 |
| Low-risk drinkers | Non-alcohol | Transition revaluation | 0.10 | 0.01 | 0.08 | 0.13 |
| Low-risk drinkers | Non-alcohol | Policy revaluation | 0.45 | 0.02 | 0.41 | 0.49 |
| Low-risk drinkers | Non-alcohol | Control | 0.12 | 0.01 | 0.09 | 0.14 |
| Low-risk drinkers | Alcohol | Reward revaluation | 0.39 | 0.02 | 0.35 | 0.43 |
| Low-risk drinkers | Alcohol | Goal-state revaluation | 0.32 | 0.02 | 0.28 | 0.35 |
| Low-risk drinkers | Alcohol | Transition revaluation | 0.18 | 0.01 | 0.15 | 0.20 |
| Low-risk drinkers | Alcohol | Policy revaluation | 0.35 | 0.02 | 0.32 | 0.39 |
| Low-risk drinkers | Alcohol | Control | 0.15 | 0.01 | 0.12 | 0.18 |
| Harmful drinkers | Non-alcohol | Reward revaluation | 0.39 | 0.02 | 0.35 | 0.43 |
| Harmful drinkers | Non-alcohol | Goal-state revaluation | 0.46 | 0.02 | 0.42 | 0.49 |
| Harmful drinkers | Non-alcohol | Transition revaluation | 0.07 | 0.01 | 0.05 | 0.09 |
| Harmful drinkers | Non-alcohol | Policy revaluation | 0.41 | 0.02 | 0.37 | 0.45 |
| Harmful drinkers | Non-alcohol | Control | 0.17 | 0.01 | 0.14 | 0.20 |
| Harmful drinkers | Alcohol | Reward revaluation | 0.28 | 0.02 | 0.25 | 0.32 |
| Harmful drinkers | Alcohol | Goal-state revaluation | 0.29 | 0.02 | 0.25 | 0.32 |
| Harmful drinkers | Alcohol | Transition revaluation | 0.13 | 0.01 | 0.11 | 0.16 |
| Harmful drinkers | Alcohol | Policy revaluation | 0.22 | 0.02 | 0.19 | 0.25 |
| Harmful drinkers | Alcohol | Control | 0.15 | 0.01 | 0.12 | 0.18 |

*Notes*. Revaluation performance is quantified as the probability of changing path preference compared to the optimal path during learning on a given test trial (see Methods). CI, confidence interval.

**Table B.** Logistic mixed-effects regression predicting revaluation performance from task condition (transition revaluation as reference category) in *n*=130 low-risk drinkers performing the task in a non-alcohol context.

| **Predictor** | **Odds ratio** | **95% CI** | ***z-value*** | ***p*** |
| --- | --- | --- | --- | --- |
| Intercept | 0.00 | 0.00-0.01 | -6.31 | <.001 |
| Reward > transition revaluation | 1191.93 | 128.79 – 11030.87 | 6.24 | <.001 |
| Goal-state > transition revaluation | 713.49 | 76.07 – 6691.88 | 5.75 | <.001 |
| Policy > transition revaluation | 765.00 | 87.51 – 6687.45 | 6.00 | <.001 |
| Control condition > transition revaluation | 0.77 | 0.05 – 12.76 | -0.18 | .855 |

*Notes*. Revaluation performance is quantified as the probability of changing path preference compared to the optimal path during learning on a given test trial (see Methods). Task condition was dummy-coded. The model included a by-subject random intercept and random slope for task condition. CI, confidence interval; z-value, Wald test z-value.

**Table C.** Logistic mixed-effects regression predicting revaluation performance from task condition (reward revaluation as reference category) in *n*=130 low-risk drinkers performing the task in a non-alcohol context.

| **Predictor** | **Odds ratio** | **95% CI** | ***z-value*** | ***p*** |
| --- | --- | --- | --- | --- |
| Intercept | 1.23 | 0.63 – 2.41 | 0.62 | 0.536 |
| Goal-state > reward revaluation | 0.60 | 0.26 – 1.40 | -1.19 | 0.236 |
| Transition > reward revaluation | 0.00 | 0.00 – 0.01 | -6.20 | **<.001** |
| Policy > reward revaluation | 0.64 | 0.33 – 1.25 | -1.31 | 0.190 |
| Control condition > reward revaluation | 0.00 | 0.00 – 0.00 | -7.15 | **<.001** |

*Notes*. Revaluation performance is quantified as the probability of changing path preference compared to the optimal path during learning on a given test trial (see Methods). Task condition was dummy-coded. The model included a by-subject random intercept and random slope for task condition. CI, confidence interval; z-value, Wald test z-value.

**Table D.** Logistic mixed-effects regression predicting revaluation performance from task condition (control condition as reference category) in *n*=130 low-risk drinkers performing the task in a non-alcohol context.

| **Predictor** | **Odds ratio** | **95% CI** | ***z-value*** | ***p*** |
| --- | --- | --- | --- | --- |
| Intercept | 0.00 | 0.00-0.01 | -7.46 | **<.001** |
| Reward revaluation > control condition | 1548.27 | 209.03 – 11467.96 | 7.19 | **<.001** |
| Goal-state revaluation > control condition | 926.79 | 125.37– 6851.14 | 6.69 | **<.001** |
| Transition revaluation > control condition | 1.30 | 0.08 – 21.60 | 0.18 | 0.855 |
| Policy revaluation > control condition | 993.70 | 140.77 – 7014.59 | 6.92 | **<.001** |

*Notes*. Revaluation performance is quantified as the probability of changing path preference compared to the optimal path during learning on a given test trial (see Methods). Task condition was dummy-coded. The model included a by-subject random intercept and random slope for task condition. CI, confidence interval; z-value, Wald test z-value.

**Table E.** Logistic mixed-effects regression predicting revaluation performance from task condition (policy revaluation as reference category) in *n*=130 low-risk drinkers performing the task in a non-alcohol context.

| **Predictor** | **Odds ratio** | **95% CI** | ***z-value*** | ***p*** |
| --- | --- | --- | --- | --- |
| Intercept | 0.79 | 0.47 – 1.33 | -0.88 | 0.378 |
| Reward > policy revaluation | 1.56 | 0.80 – 3.02 | 1.31 | 0.190 |
| Goal-state > policy revaluation | 0.93 | 0.49 – 1.78 | -0.21 | 0.833 |
| Transition > policy revaluation | 0.00 | 0.00 – 0.01 | -5.96 | **<.001** |
| Control condition > policy revaluation | 0.00 | 0.00 – 0.01 | -6.89 | **<.001** |

*Notes*. Revaluation performance is quantified as the probability of changing path preference compared to the optimal path during learning on a given test trial (see Methods). Task condition was dummy-coded. The model included a by-subject random intercept and random slope for task condition. CI, confidence interval; z-value, Wald test z-value.

**Figure D. Simulated learning and revaluation performance of a model-based (MB) strategy with a higher learning rate** $\boldsymbol{\alpha}$ **for the reward structure** $\boldsymbol{R}$ **compared to the transition structure** $\boldsymbol{T}$**across a range of parameter settings**. We ran 1000 simulation runs per condition. Learning performance was defined as the proportion of simulation runs with an optimal path preference by the end of learning. The agent was considered to have an optimal path preference by the end of learning if it had acquired a higher value for the optimal than the suboptimal state-1- and second-stage-choice, respectively, and if it had chosen the optimal path in at least three out of the last five learning trials. Runs which fulfilled these criteria were taken as the base population to evaluate revaluation performance, i.e. the proportion of runs in which the agent changed its path preference on a test trial after re-learning compared to the optimal path during the learning phase (see Methods). The test trial was deterministic, favoring the state-1- and second-stage-action with the highest value, respectively. The learning rate $\alpha_{R}$ for the reward structure was set to 0.9, whereas the learning rate $\alpha_{T}$ for the transition structure was varied at lower values. For model details and simulation procedure, see Methods. Bars represent means, error bars represent standard errors of the mean.

**Figure E. Proportion of free-choice learning trials where the optimal path was chosen, per trial, group, and context (*n*=130 participants per subplot).** Learning trial number starts at 5 as the first four trials were forced-choice. Lines represent means, shaded areas represent standard errors of the mean.

**Figure F. Simulated learning and revaluation performance of random-policy successor representation (SR) strategies across a range of parameter settings**. We ran 1000 simulation runs per model and condition. Learning performance was defined as the proportion of simulation runs with an optimal path preference by the end of learning. Agents were considered to have an optimal path preference by the end of learning if they had acquired a higher value for the optimal than the suboptimal state-1- and second-stage-choice, respectively, and if they had chosen the optimal path in at least three out of the last five learning trials. Runs which fulfilled these criteria were taken as the base population to evaluate revaluation performance, i.e. the proportion of runs in which an agent changed its path preference on a test trial after re-learning compared to the optimal path during the learning phase (see Methods). The test trial was deterministic, favoring the state-1- and second-stage-action with the highest value, respectively. All models learned the transition matrix $T$ and the reward vector $R$ in a model-based way during the initial four forced-choice learning trials and then converted $T$ to a random-policy successor matrix $M$, and $R$ to a weight vector $w$. The variant with rigid $M$ updated only $w$ throughout the free-choice learning trials, whereas the variant with rigid $M$ and $w$ updated neither $M$ nor $w$ during free-choice learning trials. Both algorithms updated $M$ and $w$ during re-learning trials. For model details and simulation procedure, see Methods. Bars represent means, error bars represent standard errors of the mean. $\alpha$, learning rate used to update $R$, $w$, and $M$.

**Figure G. Simulated learning and revaluation performance of rigid goal-based reduced random-policy successor representation (SR) strategies across a range of parameter settings**. We ran 1000 simulation runs per model and condition. Learning performance was defined as the proportion of simulation runs with an optimal path preference by the end of learning. Agents were considered to have an optimal path preference by the end of learning if they had acquired a higher value for the optimal than the suboptimal state-1- and second-stage-choice, respectively, and if they had chosen the optimal path in at least three out of the last five learning trials. Runs which fulfilled these criteria were taken as the base population to evaluate revaluation performance, i.e. the proportion of runs in which an agent changed its path preference on a test trial after re-learning compared to the optimal path during the learning phase. The test trial was deterministic, favoring the state-1- and second-stage-action with the highest value, respectively. All models learned the transition matrix $T$ and the reward vector $R$ in a model-based way during the initial four forced-choice trials, then converted $T$ into a random-policy successor matrix $M$ and $R$ to a weight vector $w$, and updated $w$ throughout the free-choice learning trials whereas $M$ stayed rigid. Before re-learning, $M$ was transformed to a goal-based reduced version. Goal-state-action-pairs were either only the pairs that led to a reward during learning (2 goals), additionally the action from final state 10 (3 goals), or additionally the third state-action-pair on the same stage as the reward-associated state-action-pairs during learning (4 goals). For model details and simulation procedure, see Methods. Bars represent means, error bars represent standard errors of the mean. Note that the reduced random-policy SR agents with 2 or 3 goal-state-action-pairs cannot adapt to policy revaluation as neither the state-action-pair newly associated with a reward nor any successor state-action-pair is considered a goal. $\alpha$, learning rate used to update $R$, $w$, and $M$.

**Figure H**. **Model comparison results.** Comparison was based on **A** Akaike’s Information Criterion (AIC) and **B** Bayesian Information Criterion (BIC)**.** For reduced (random-policy) SR algorithms, goal-state-action-pairs were either only the pairs that led to a reward during learning (2 goals), additionally the action from final state 10 (3 goals), or additionally the third state-action-pair on the same stage as the reward-associated state-action-pairs during learning (4 goals). For model details and fitting procedure, see Methods. Bars represent means, error bars represent standard errors of the mean. MB, model-based; MF, model-free; $M$, successor matrix; $R$, reward vector; SR, successor representation; $T$, transition matrix; $w$, weight vector; $\alpha$, learning rate.

**Table F.** Parameter recoverability for alternative reinforcement learning models.

| **Model** | **Low-risk drinkers performing task in non-alcohol context (*n*=130)** | | | **Full sample (*N*=520)** | | |
| --- | --- | --- | --- | --- | --- | --- |
|  | $\boldsymbol{\alpha}$ | $\boldsymbol{\gamma}$ | $\boldsymbol{\omega}$ | $\boldsymbol{\alpha}$ | $\boldsymbol{\gamma}$ | $\boldsymbol{\omega}$ |
| Hybrid MF-reduced random-policy SR (4 goals) | .80 | .92 | .90 | .77 | .91 | .87 |
| Hybrid MF-random-policy SR (rigid $M$ & $w$) | .77 | .93 | .96 | .67 | .94 | .96 |
| Hybrid MF-random-policy SR (rigid $M$) | .77 | .91 | .77 | .72 | .92 | .84 |
| Hybrid MF-MB with $\alpha\left( R \right)$ & $\alpha(T)$ | $\alpha_{R}:$.57, $\alpha_{T}:$ .64 | .89 | .92 | $\alpha_{R}:$.57, $\alpha_{T}:$ .66 | .90 | .94 |
| Reduced random-policy SR (4 goals) | .87 | .82 | - | .85 | .84 | - |
| Random-policy SR (rigid $M$ & $w$) | .50 | .97 | - | .46 | .92 | - |
| Random-policy SR (rigid $M$) | .81 | .81 | - | .81 | .80 | - |
| MB with $\alpha\left( R \right)$ & $\alpha(T)$ | $\alpha_{R}:$.90, $\alpha_{T}:$ .85 | .89 | - | $\alpha_{R}:$.92, $\alpha_{T}:$ .78 | .88 | - |
| Reduced random-policy SR (3 goals) | .92 | .80 | - | .85 | .78 | - |
| Reduced random-policy SR (2 goals) | .92 | .80 | - | .94 | .87 | - |
| RedSR (4 goals) | .81 | .91 | - | .80 | .91 | - |
| RedSR (3 goals) | .79 | .90 | - | .84 | .91 | - |
| RedSR (2 goals) | .86 | .87 | - | .84 | .88 | - |
| SR | .83 | .83 | - | .86 | .87 | - |
| MB | .52 | .71 | - | .58 | .74 | - |
| MF | .70 | .88 | - | .66 | .88 | - |

*Notes*. Values are Spearman’s rank correlation coefficients. For reduced (random-policy) SR algorithms, goal-state-action-pairs were either only the pairs that led to a reward during learning (2 goals), additionally the action from final state 10 (3 goals), or additionally the third state-action-pair on the same stage as the reward-associated state-action-pairs during learning (4 goals). For model details and parameter recovery procedure, see Methods. MB, model-based; MF, model-free; $M$, successor matrix; $R$, reward vector; SR, successor representation; $T$, transition matrix; $w$, weight vector; $\alpha$, learning rate; $\gamma$, time discount factor; $\omega$, weighting parameter (higher values indicate more non-MF contributions).

**Figure I.** **Parametric bootstrapping simulations** **of revaluation performance of alternative RL models based on individually fitted parameter estimates for low-risk drinkers performing the task in a non-alcohol context**. Simulated revaluation performance (bars and black error bars) is overlaid by observed revaluation performance in *n*=130 humans (red dots and error bars). Simulations are based on individually estimated parameters (one simulation run per participant). A simulated participant was considered to have an optimal path preference by the end of learning in a given condition if they had acquired a higher value for the optimal than the suboptimal state-1- and second-stage-choice, respectively, and if they had chosen the optimal path in at least three out of the last five learning trials. Only simulated participants who fulfilled these criteria in all conditions were taken as the base population to evaluate revaluation performance, i.e. the proportion of test trials in which an agent changed its path preference after re-learning compared to the optimal path during the learning phase (see Methods). We simulated five probabilistic test trials per participant and condition. For the reduced random-policy SR algorithm, goal-state-action-pairs were the pairs that led to a reward during learning plus the third state-action-pair on the same stage as the reward-associated state-action-pairs during learning plus the action from final state 10 (4 goals). Note that (hybrid MF-) (reduced) random-policy SR models were descriptively better at reproducing the contrast between reward, goal-state, and policy revaluation compared to transition revaluation, respectively, in combination with the not significantly higher transition revaluation compared to control performance which we observed in humans than the (hybrid MF-) MB learner with $\alpha_{R}$ and $\alpha_{T}$. For model details and parametric bootstrapping simulation procedure, see Methods. Bars and red dots represent means, error bars represent standard errors of the mean. MF, model-free; $M$, successor matrix; $R$, reward vector; SR, successor representation; $T$, transition matrix; $w$, weight vector; $\alpha$, learning rate used to update the reward vector $R$, the weight vector $w$, value estimates $Q$, as well as the successor matrix $M$.

**Figure J. Test phase response times across all states by group and context (*n*=130 participants per subplot)**. Boxplots show the median, 25^th^ and 75^th^ percentiles, and the smallest and largest value up to maximum 150% of the inter-quartile range. Violin plots depict the data distribution.

**Table G.** Test phase response times in ms per group, context, and task condition.

| **Group** | **Context** | **Condition** | ***M*** | ***SE*** | **95% CI lower limit** | **95% CI upper limit** |
| --- | --- | --- | --- | --- | --- | --- |
| Low-risk drinkers | Non-alcohol | Reward revaluation | 576.07 | 18.01 | 540.74 | 611.39 |
| Low-risk drinkers | Non-alcohol | Goal-state revaluation | 569.28 | 19.24 | 531.53 | 607.03 |
| Low-risk drinkers | Non-alcohol | Transition revaluation | 528.89 | 19.68 | 490.29 | 567.48 |
| Low-risk drinkers | Non-alcohol | Policy revaluation | 596.35 | 19.68 | 557.75 | 634.95 |
| Low-risk drinkers | Non-alcohol | Control | 439.91 | 13.33 | 413.75 | 466.07 |
| Low-risk drinkers | Alcohol | Reward revaluation | 602.62 | 16.18 | 570.88 | 634.36 |
| Low-risk drinkers | Alcohol | Goal-state revaluation | 658.06 | 18.90 | 620.98 | 695.15 |
| Low-risk drinkers | Alcohol | Transition revaluation | 588.83 | 17.72 | 554.07 | 623.59 |
| Low-risk drinkers | Alcohol | Policy revaluation | 617.41 | 18.43 | 581.25 | 653.57 |
| Low-risk drinkers | Alcohol | Control | 551.55 | 17.35 | 517.52 | 585.58 |
| Harmful drinkers | Non-alcohol | Reward revaluation | 602.17 | 18.92 | 565.06 | 639.29 |
| Harmful drinkers | Non-alcohol | Goal-state revaluation | 582.35 | 18.80 | 545.47 | 619.23 |
| Harmful drinkers | Non-alcohol | Transition revaluation | 508.39 | 14.85 | 479.25 | 537.54 |
| Harmful drinkers | Non-alcohol | Policy revaluation | 570.02 | 16.97 | 536.73 | 603.31 |
| Harmful drinkers | Non-alcohol | Control | 474.65 | 13.71 | 447.76 | 501.54 |
| Harmful drinkers | Alcohol | Reward revaluation | 615.53 | 18.13 | 579.96 | 651.10 |
| Harmful drinkers | Alcohol | Goal-state revaluation | 641.94 | 18.65 | 605.36 | 678.52 |
| Harmful drinkers | Alcohol | Transition revaluation | 581.77 | 18.91 | 544.67 | 618.86 |
| Harmful drinkers | Alcohol | Policy revaluation | 679.91 | 21.34 | 638.05 | 721.77 |
| Harmful drinkers | Alcohol | Control | 586.31 | 19.14 | 548.76 | 623.86 |

*Notes*. CI, confidence interval.

**Table H.** Linear mixed-effects regression predicting test phase response times from task condition (transition revaluation as reference category) in *n*=130 low-risk drinkers performing the task in a non-alcohol context.

|  | **Standard estimation** | | | | | **Robust estimation** | |
| --- | --- | --- | --- | --- | --- | --- | --- |
| **Predictors** | $\boldsymbol{\beta}$ | **95% CI** | **t-value** | ***p*** | ***df*** | $\boldsymbol{\beta}$ | **95% CI** |
| Intercept | 528.89 | 466.81 – 590.96 | 16.85 | **<.001** | 130.18 | 389.06 | **401.54 – 430.18** |
| Reward > transition revaluation | 47.18 | -23.52 – 117.89 | 1.32 | .189 | 130.00 | 42.71 | **29.34 – 70.16** |
| Goal-state > transition revaluation | 40.39 | -32.15 – 112.94 | 1.10 | .273 | 130.01 | 33.50 | -2.79 – 39.13 |
| Policy > transition revaluation | 67.47 | -2.53 – 137.46 | 1.91 | .059 | 130.01 | 47.95 | **26.18 – 66.64** |
| Control condition > transition revaluation | -88.97 | -147.55 – -30.40 | -3.00 | **.003** | 161.88 | -43.14 | **-69.86 – -29.36** |

*Notes*. Task condition was dummy-coded. The model included a by-subject random intercept and random slope for task condition. We used Satterthwaite’s degrees of freedom. As relevant violations of model assumptions were detected visually, we re-estimated the model in a robust fashion using the *robustlmm* package [1]. This model confirmed all significant effects and revealed additional significant effects based on confidence intervals (CIs) estimated using the *confintROB* package [2].

**Table I.** Logistic mixed-effects regression predicting revaluation performance from task condition (transition revaluation as reference category), group (high-risk, low-risk drinkers) and context (alcohol, non-alcohol context) in n=520 participants.

| **Predictor** | **Odds ratio** | **95% CI** | ***z-value*** | ***p*** |
| --- | --- | --- | --- | --- |
| Intercept | 0.02 | 0.01 – 0.03 | -11.74 | **<.001** |
| Group | 0.61 | 0.31 – 1.20 | -1.43 | 0.152 |
| Context | 2.67 | 1.36 – 5.25 | 2.86 | **0.004** |
| Reward > transition revaluation | 24.99 | 11.76 – 53.09 | 8.37 | **<.001** |
| Goal-state > transition revaluation | 23.96 | 11.24 – 51.07 | 8.22 | **<.001** |
| Policy > transition revaluation | 19.64 | 9.37 – 41.18 | 7.88 | **<.001** |
| Control condition > transition revaluation | 0.06 | 0.02 – 0.21 | -4.52 | **<.001** |
| Group * Context | 0.76 | 0.20 – 2.89 | -0.40 | 0.686 |
| Group * [Reward > transition revaluation] | 0.50 | 0.21 – 1.19 | -1.56 | 0.118 |
| Group * [Goal-state > transition revaluation] | 1.43 | 0.59 – 3.46 | 0.80 | 0.426 |
| Group * [Policy > transition revaluation] | 0.46 | 0.28 – 1.45 | -1.08 | 0.282 |
| Group * [Control condition > transition revaluation] | 2.39 | 0.74 – 7.72 | 1.46 | 0.145 |
| Context * [Reward > transition revaluation] | 0.12 | 0.05 – 0.28 | -4.82 | **<.001** |
| Context * [Goal-state > transition revaluation] | 0.09 | 0.04 – 0.21 | -5.38 | **<.001** |
| Context * [Policy > transition revaluation] | 0.08 | 0.04 – 0.19 | -5.86 | **<.001** |
| Context * [Control condition > transition revaluation] | 0.40 | 0.12 – 1.29 | -1.54 | 0.124 |
| Group * Context * [Reward > transition revaluation] | 1.18 | 0.21 – 6.60 | 0.19 | 0.848 |
| Group * Context * [Goal-state > transition revaluation] | 0.85 | 0.15 – 4.92 | -0.18 | 0.0857 |
| Group * Context * [Policy > transition revaluation] | 0.54 | 0.11 – 2.74 | -0.74 | 0.459 |
| Group * Context * [Control condition > transition revaluation] | 0.67 | 0.07 – 6.89 | -0.33 | 0.738 |

*Notes*. Revaluation performance is quantified as the probability of changing path preference compared to the optimal path during learning on a given test trial (see Methods). Task condition was dummy-coded, group and context were sum-contrast-coded. The model included a by-subject random intercept and random slope for task condition. Bonferroni-corrected post-hoc tests are reported in the main text. CI, confidence interval; z-value, Wald test z-value.

**Table J.** Logistic mixed-effects regression predicting revaluation performance from task condition (control condition as reference category), group (high-risk, low-risk drinkers) and context (alcohol, non-alcohol context) in *n*=520 participants.

| **Predictor** | **Odds ratio** | **95% CI** | ***z-value*** | ***p*** |
| --- | --- | --- | --- | --- |
| Intercept | 0.00 | 0.00 – 0.00 | -14.00 | **<.001** |
| Group | 1.46 | 0.57 – 3.76 | 0.79 | 0.430 |
| Context | 1.07 | 0.42 – 2.73 | 0.13 | 0.894 |
| Reward revaluation > control condition | 408.29 | 143.59 – 1161.01 | 11.28 | **<.001** |
| Goal-state revaluation > control condition | 391.40 | 139.45 – 1098.54 | 11.34 | **<.001** |
| Transition revaluation > control condition | 16.34 | 4.89 – 54.63 | 4.54 | **<.001** |
| Policy revaluation > control condition | 320.90 | 113.73 – 905.42 | 10.90 | **<.001** |
| Group * Context | 0.51 | 0.08 – 3.33 | -0.70 | 0.481 |
| Group * [Reward revaluation > control condition] | 0.21 | 0.06 – 0.69 | -2.56 | **0.011** |
| Group * [Goal-state revaluation > control condition] | 0.60 | 0.19 – 1.89 | -0.88 | 0.381 |
| Group * [Transition revaluation > control condition] | 0.42 | 0.13 – 1.35 | -1.46 | 0.145 |
| Group * [Policy revaluation > control condition] | 0.27 | 0.08 – 0.86 | -2.22 | **0.027** |
| Context * [Reward revaluation > control condition] | 0.29 | 0.09 – 0.97 | -2.00 | **0.045** |
| Context * [Goal-state revaluation > control condition] | 0.22 | 0.07 – 0.68 | -2.61 | **0.009** |
| Context * [Transition revaluation > control condition] | 2.51 | 0.78 – 8.08 | 1.54 | 0.123 |
| Context * [Policy revaluation > control condition] | 0.21 | 0.07 – 0.69 | -2.59 | **0.010** |
| Group * Context * [Reward revaluation > control condition] | 1.76 | 0.16 – 18.94 | 0.47 | 0.641 |
| Group * Context * [Goal-state revaluation > control condition] | 1.27 | 0.13 – 12.44 | 0.20 | 0.840 |
| Group * Context * [Transition revaluation > control condition] | 1.49 | 0.14 – 15.28 | 0.33 | 0.738 |
| Group * Context * [Policy revaluation > control condition] | 0.81 | 0.08 – 8.20 | -0.18 | 0.857 |

*Notes*. Revaluation performance is quantified as the probability of changing path preference compared to the optimal path during learning on a given test trial (see Methods). Task condition was dummy-coded, group and context were sum-contrast-coded. The model included a by-subject random intercept and random slope for task condition. We performed Bonferroni-corrected post-hoc tests using estimated marginal means for significant interactions, revealing that the contrasts ‘reward revaluation>control condition’ (high-risk drinkers: *OR*=186.53, *z*=8.42, *p*<.001; low-risk drinkers: *OR*=893.70, *z*=11.17, *p*<.001) and ‘policy revaluation>control condition’ (high-risk drinkers: *OR*=165.85, *z*=8.34, *p*<.001; low-risk drinkers: *OR*=620.92, *z*=10.69, *p*<.001) were smaller in high-risk drinkers but significant in both groups, and that the contrasts ‘reward revaluation>control condition (alcohol context: *OR*=221.23, *z*=8.76, *p*<.001; non-alcohol context: *OR*=753.54, *z*=10.81, *p*<.001), ‘goal-state revaluation>control condition’ (alcohol context: *OR*=181.59, *z*=8.60, *p*<.001; non-alcohol context: *OR*=843.61, *z*=11.20, *p*<.001), and ‘policy revaluation>control condition’ (alcohol context: *OR*=147.96, *z*=8.19, *p*<.001; non-alcohol context: *OR*=695.97, *z*=10.81, *p*<.001) were smaller in the alcohol context but significant in both contexts. CI, confidence interval; z-value, Wald test z-value.

**Table K.** Logistic mixed-effects regression predicting the probability of choosing the optimal path per trial in the last 5 learning trials from task condition (control condition as reference category), group (high-risk, low-risk drinkers) and context (alcohol, non-alcohol context) in *n*=520 participants.

| **Predictor** | **Odds ratio** | **95% CI** | ***z-value*** | ***p*** |
| --- | --- | --- | --- | --- |
| Intercept | 3744.11 | 996.48 – 14067.86 | 12.18 | **<.001** |
| Group | 1.08 | 0.31 – 3.76 | 0.11 | 0.909 |
| Context | 1.35 | 0.39 – 4.74 | 0.47 | 0.635 |
| Reward revaluation > control condition | 1.17 | 0.17 – 8.02 | 0.16 | 0.873 |
| Goal-state revaluation > control condition | 0.40 | 0.06 – 2.74 | -0.94 | 0.350 |
| Transition revaluation > control condition | 1.31 | 0.19 – 8.78 | 0.28 | 0.783 |
| Policy revaluation > control condition | 2.71 | 0.36 – 20.43 | 0.97 | 0.332 |
| Group * Context | 1.29 | 0.11 – 14.97 | 0.21 | 0.837 |
| Group * [Reward revaluation > control condition] | 1.25 | 0.21 – 7.51 | 0.25 | 0.804 |
| Group * [Goal-state revaluation > control condition] | 0.80 | 0.16 – 4.12 | -0.27 | 0.791 |
| Group * [Transition revaluation > control condition] | 0.83 | 0.13 – 5.31 | -0.19 | 0.847 |
| Group * [Policy revaluation > control condition] | 0.78 | 0.10 – 5.93 | -0.23 | 0.814 |
| Context * [Reward revaluation > control condition] | 1.18 | 0.20 – 7.06 | 0.18 | 0.859 |
| Context * [Goal-state revaluation > control condition] | 1.24 | 0.24 – 6.38 | 0.26 | 0.798 |
| Context * [Transition revaluation > control condition] | 0.78 | 0.12 – 5.00 | -0.26 | 0.796 |
| Context * [Policy revaluation > control condition] | 0.91 | 0.12 – 6.87 | -0.09 | 0.924 |
| Group * Context * [Reward revaluation > control condition] | 0.80 | 0.02 – 27.30 | -0.13 | 0.899 |
| Group * Context * [Goal-state revaluation > control condition] | 0.68 | 0.03 – 16.78 | -0.24 | 0.811 |
| Group * Context * [Transition revaluation > control condition] | 1.40 | 0.04 – 53.80 | 0.18 | 0.855 |
| Group * Context * [Policy revaluation > control condition] | 0.74 | 0.01 – 39.53 | -0.15 | 0.881 |

*Notes*. Task condition was dummy-coded, group and context were sum-contrast-coded. The model included a by-subject random intercept and random slope for task condition. CI, confidence interval; z-value, Wald test z-value.

**Table L.** Linear mixed-effects regression predicting test phase response times in ms from task condition (reward revaluation as reference category), group (high-risk, low-risk drinkers), and context (alcohol, non-alcohol context) in *n*=520 participants.

|  | **Standard estimation** | | | | | **Robust estimation** | |
| --- | --- | --- | --- | --- | --- | --- | --- |
| **Predictors** | $\boldsymbol{\beta}$ | **95% CI** | ***t*-value** | ***p*** | ***df*** | $\boldsymbol{\beta}$ | **95% CI** |
| Intercept | 599.10 | 572.12 – 626.08 | 43.62 | **<.001** | 520.03 | 467.20 | **469.90 – 487.14** |
| Group | 19.51 | -34.45 – 73.47 | 0.71 | 0.478 | 520.00 | 5.89 | -8.58 – 25.11 |
| Context | 19.95 | -34.01 – 73.91 | 0.73 | 0.468 | 520.00 | 37.79 | **23.91 – 59.14** |
| Goal-state > reward revaluation | 13.81 | -22.92 – 50.54 | 0.74 | 0.460 | 520.00 | 0.98 | -11.00 – 13.07 |
| Transition > reward revaluation | -47.13 | -78.15 – -16.11 | -2.99 | **0.003** | 520.00 | -29.40 | **-41.65 – -17.07** |
| Policy > reward revaluation | 16.82 | -18.57 – 52.22 | 0.93 | 0.351 | 519.96 | 4.49 | -7.57 – 16.98 |
| Control condition > reward revaluation | -85.99 | -114.96 – -57.03 | -5.83 | **<.001** | 520.00 | -52.05 | **-63.80 – -39.92** |
| Group * Context | -13.20 | -121.12 – 94.72 | -0.24 | 0.810 | 520.00 | -17.35 | -41.62 – 27.43 |
| Group * [Goal-state > reward revaluation] | -21.03 | -94.49 – 52.42 | -0.56 | 0.574 | 520.00 | 2.47 | -21.26 – 26.61 |
| Group * [Transition > reward revaluation] | -33.28 | -95.32 – 28.75 | -1.05 | 0.292 | 520.00 | 1.48 | -22.78 – 24.74 |
| Group * [Policy > reward revaluation] | -1.42 | -72.21 – 69.37 | -0.04 | 0.969 | 519.96 | 3.14 | -21.87 – 27.57 |
| Group * [Control condition > reward revaluation] | 15.24 | -42.69 – 73.18 | 0.52 | 0.605 | 520.00 | 16.31 | -7.73 – 40.08 |
| Context * [Goal-state > reward revaluation] | 54.23 | -19.22 – 127.69 | 1.45 | 0.148 | 520.00 | 17.42 | -7.35 – 41.73 |
| Context * [Transition > reward revaluation] | 46.70 | -15.33 – 108.74 | 1.48 | 0.140 | 520.00 | 16.81 | -7.06 – 41.15 |
| Context * [Policy > reward revaluation] | 45.52 | -25.27 – 116.31 | 1.26 | 0.207 | 519.96 | 14.05 | -9.86 – 37.72 |
| Context * [Control condition > reward revaluation] | 91.69 | 33.76 – 149.63 | 3.11 | **0.002** | 520.00 | 22.15 | -2.69 – 46.40 |
| Group * Context * [Goal-state > reward revaluation] | -16.00 | -162.91 – 130.91 | -0.21 | 0.831 | 520.00 | -18.52 | -67.67 – 29.37 |
| Group * Context * [Transition > reward revaluation] | 26.62 | -97.44 – 150.69 | 0.42 | 0.673 | 520.00 | -5.08 | -54.27 – 43.35 |
| Group * Context * [Policy > reward revaluation] | 102.03 | -39.56 – 243.61 | 1.42 | 0.157 | 519.96 | 40.01 | -8.94 – 88.71 |
| Group * Context * [Control condition > reward revaluation] | 13.22 | -102.65 – 129.08 | 0.22 | 0.823 | 520.00 | -18.72 | -67.20 – 30.13 |

*Notes*. Task condition was dummy-coded, group and context were sum-contrast-coded. The model included a by-subject random intercept and random slope for task condition. We performed post-hoc tests using estimated marginal means for significant interactions, revealing that response times in the control condition were shorter than those in the reward revaluation condition only in the non-alcohol ($\beta$=-131.84, *z*=-6.32, *p*<.001) but not in the alcohol context ($\beta$=-40.15, *z*=-1.93, *p*=.054). We used Satterthwaite’s degrees of freedom. As relevant violations of model assumptions were detected visually, we re-estimated the model in a robust fashion without random slope for condition due to convergence issues using the *robustlmm* package [1]. This model confirmed all significant effects besides the interaction between the contrast ‘control condition>reward revaluation’ and context based on confidence intervals (CIs) estimated using the *confintROB* package [2]. It also revealed an additional significant main effect of context.

**Table M.** Logistic mixed-effects regression predicting revaluation performance from task condition (reward revaluation as reference category) and group (high-risk, low-risk drinkers) in *n*=260 participants performing the task in an alcohol context.

| **Predictor** | **Odds ratio** | **95% CI** | ***z-value*** | ***p*** |
| --- | --- | --- | --- | --- |
| Intercept | 0.20 | 0.12 – 0.35 | -5.83 | **<.001** |
| Goal-state > reward revaluation | 0.94 | 0.54-1.63 | -0.21 | 0.834 |
| Transition > reward revaluation | 0.16 | 0.07 – 0.36 | -4.42 | **<.001** |
| Policy > reward revaluation | 0.67 | 0.36 – 1.25 | -1.26 | 0.209 |
| Control condition > reward revaluation | 0.00 | 0.00 – 0.01 | -7.89 | **<.001** |
| Group | 0.28 | 0.10 – 0.76 | -2.49 | **0.013** |
| [Goal-state > reward revaluation] * Group | 2.58 | 0.97 – 6.86 | 1.90 | 0.057 |
| [Transition > reward revaluation] * Group | 1.98 | 0.63 – 6-28 | 1.16 | 0.245 |
| [Policy > reward revaluation] * Group | 0.88 | 0.29 – 2.63 | -0.23 | 0.816 |
| [Control condition > reward revaluation] * Group | 3.69 | 0.63 – 21.61 | 1.45 | 0.147 |

*Notes*. Revaluation performance is quantified as the probability of changing path preference compared to the optimal path during learning on a given test trial (see Methods). Task condition was dummy-coded, group was sum-contrast-coded. The model included a by-subject random intercept and random slope for task condition. CI, confidence interval; z-value, Wald test z-value.

**Table N.** Logistic mixed-effects regression predicting revaluation performance from task condition (reward revaluation as reference category), group (high-risk, low-risk drinkers) and context (alcohol, non-alcohol context) in *n*=520 participants.

| **Predictor** | **Odds ratio** | **95% CI** | ***z-value*** | ***p*** |
| --- | --- | --- | --- | --- |
| Intercept | 0.38 | 0.27 – 0.53 | -5.61 | **<.001** |
| Group | 0.31 | 0.16 – 0.60 | -3.48 | **<.001** |
| Context | 0.31 | 0.16 – 0.61 | -3.41 | **0.001** |
| Goal-state > reward revaluation | 0.96 | 0.66 – 1.39 | -0.22 | 0.822 |
| Transition > reward revaluation | 0.04 | 0.02-0.09 | -8.34 | **<.001** |
| Policy > reward revaluation | 0.79 | 0.54 – 1.14 | -1.28 | 0.202 |
| Control condition > reward revaluation | 0.00 | 0.00 – 0.01 | -11.22 | **<.001** |
| Group * Context | 0.90 | 0.24 – 3.32 | -0.16 | 0.871 |
| Group * [Goal-state > reward revaluation] | 2.87 | 1.40 – 5.88 | 2.87 | **0.004** |
| Group * [Transition > reward revaluation] | 2.00 | 0.84 – 4.79 | 1.56 | 0.118 |
| Group * [Policy > reward revaluation] | 1.28 | 0.62 – 2.63 | 0.67 | 0.503 |
| Group * [Control condition > reward revaluation] | 4.79 | 1.44 – 15.92 | 2.56 | **0.011** |
| Context * [Goal-state > reward revaluation] | 0.73 | 0.35 – 1.52 | -0.83 | 0.406 |
| Context * [Transition > reward revaluation] | 8.54 | 3.57 – 20.43 | 4.82 | **<.001** |
| Context * [Policy > reward revaluation] | 0.72 | 0.35 – 1.50 | -0.87 | 0.386 |
| Context * [Control condition > reward revaluation] | 3.41 | 1.03 – 11.29 | 2.00 | **0.045** |
| Group * Context * [Goal-state > reward revaluation] | 0.72 | 0.18 – 2.95 | -0.46 | 0.647 |
| Group * Context * [Transition > reward revaluation] | 0.85 | 0.15 – 4.73 | -0.19 | 0.848 |
| Group * Context * [Policy > reward revaluation] | 0.46 | 0.11 – 1.87 | -1.09 | 0.277 |
| Group * Context * [Control condition > reward revaluation] | 0.57 | 0.05 – 6.14 | -0.47 | 0.641 |

*Notes*. Revaluation performance is quantified as the probability of changing path preference compared to the optimal path during learning on a given test trial (see Methods). Task condition was dummy-coded, group and context were sum-contrast-coded. The model included a by-subject random intercept and random slope for task condition. We performed Bonferroni-corrected post-hoc tests besides the ones reported in the main text using estimated marginal means for significant interactions, revealing that the contrast ‘goal-state>reward revaluation’ was less negative in high-risk drinkers (*OR*=1.62, *z*=1.80, *p*=.145) than in low-risk drinkers (*OR*=0.57, *z*=-2.23, *p*=.052) and that the same was true for the contrast ‘control condition>reward revaluation’ (high-risk drinkers: *OR*=0.01, *z*=-8.40, *p*<.001, low-risk drinkers: *OR*=0.001, *z*=-11.11, *p*<.001). Moreover, the contrast ‘transition revaluation>reward revaluation’ was smaller in the alcohol context (*OR*=0.12, *z*=-4.93, *p*<.001) compared to the non-alcohol context (*OR*=0.01, *z*=-9.4, *p*<.001) and the same was true for the contrast ‘control condition>reward revaluation’ (alcohol context: *OR*=0.005, *z*=-8.74, *p*<.001, non-alcohol context: *OR*=0.001, *z*=-10.77, *p*<.001). CI, confidence interval; z-value, Wald test z-value.

**Figure K. Parametric bootstrapping simulations of learning and revaluation performance of all *a priori* defined and alternative RL models based on individually fitted parameter estimates for the full sample.** Simulated learning and revaluation performance (bars and black error bars) as well as observed revaluation performance in *N*=520 humans (red dots and error bars) are depicted. Simulations are based on individually estimated parameters (one simulation run per participant). Learning performance was defined as the proportion of simulated participants with an optimal path preference by the end of learning. A simulated participant was considered to have an optimal path preference by the end of learning in a given condition if they had acquired a higher value for the optimal than the suboptimal state-1- and second-stage-choice, respectively, and if they had chosen the optimal path in at least three out of the last five learning trials. Only simulated participants who fulfilled these criteria in all conditions were taken as the base population to evaluate revaluation performance, i.e. the proportion of test trials in which an agent changed its path preference after re-learning compared to the optimal path during the learning phase (see Methods). We simulated five probabilistic test trials per participant and condition. For reduced (random-policy) SR algorithms, goal-state-action-pairs were either only the pairs that led to a reward during learning (2 goals), additionally the action from final state 10 (3 goals), or additionally the third state-action-pair on the same stage as the reward-associated state-action-pairs during learning (4 goals). For model details and parametric bootstrapping simulation procedure, see Methods. Bars and red dots represent means, error bars represent standard errors of the mean. MB, model-based; MF, model-free; $M$, successor matrix; $R$, reward vector; SR, successor representation; $T$, transition matrix; $w$, weight vector; $\alpha$, learning rate.

**Table O.** Linear mixed-effects regression predicting test phase response times in ms from task condition (transition revaluation as reference category), group (high-risk, low-risk drinkers), and context (alcohol, non-alcohol context) in *n*=520 participants.

|  | **Standard estimation** | | | | | **Robust estimation** | |
| --- | --- | --- | --- | --- | --- | --- | --- |
| **Predictors** | $\boldsymbol{\beta}$ | **95% CI** | **t-value** | ***p*** | ***df*** | $\boldsymbol{\beta}$ | **95% CI** |
| Intercept | 551.97 | 524.82 – 579.11 | 39.95 | **<.001** | 520.03 | 437.80 | **440.46 – 457.53** |
| Group | -13.78 | -68.07 – 40.51 | -0.50 | 0.618 | 520.03 | 7.37 | -7.69 – 26.99 |
| Context | 66.66 | 12.37 – 120.95 | 2.41 | **0.016** | 520.03 | 54.60 | **42.00 – 75.57** |
| Reward > transition revaluation | 47.13 | 16.11 – 78.15 | 2.99 | **0.003** | 520.00 | 29.40 | **17.67 – 41.78** |
| Goal-state > transition revaluation | 60.94 | 26.37 – 95.51 | 3.46 | **0.001** | 520.00 | 30.37 | **18.66 – 42.67** |
| Policy > transition revaluation | 63.95 | 32.11 – 95.79 | 3.95 | **<.001** | 520.64 | 33.89 | **21.90 – 46.39** |
| Control condition > transition revaluation | -38.86 | -65.76 – -11.97 | -2.84 | **0.005** | 520.64 | -22.65 | **-34.44 – -10.40** |
| Group * Context | 13.43 | -95.15 – 122.01 | 0.24 | 0.808 | 520.03 | -22.43 | -46.07 – 21.46 |
| Group * [Reward > transition revaluation] | 33.28 | -28.75 – 95.32 | 1.05 | 0.292 | 520.00 | -1.48 | -25.63 – 22.42 |
| Group * [Goal-state > transition revaluation] | 12.25 | -56.88 – 81.39 | 0.35 | 0.728 | 520.00 | 1.00 | -23.20 – 25.67 |
| Group * [Policy > transition revaluation] | 31.86 | -31.82 – 95.54 | 0.98 | 0.326 | 520.64 | 1.67 | -22.22 – 26.34 |
| Group * [Control condition > transition revaluation] | 48.53 | -5.26 – 102.32 | 1.77 | 0.077 | 520.64 | 14.83 | -9.04 – 38.95 |
| Context * [Reward > transition revaluation] | -46.70 | -108.74 – 15.33 | -1.48 | 0.140 | 520.00 | -16.81 | -41.32 – 7.07 |
| Context * [Goal-state > transition revaluation] | 7.53 | -61.61 – 76.66 | 0.21 | 0.831 | 520.00 | 0.61 | -23.35 – 24.76 |
| Context * [Policy > transition revaluation] | -1.18 | -64.87 – 62.50 | -0.04 | 0.971 | 520.64 | -2.76 | -26.68 – 21.01 |
| Context * [Control condition > transition revaluation] | 44.99 | -8.80 – 98.78 | 1.64 | 0.101 | 520.64 | 5.34 | -18.43 – 29.24 |
| Group * Context * [Reward > transition revaluation] | -26.62 | -150.69 – 97.44 | -0.42 | 0.673 | 520.00 | 5.08 | -42.86 – 53.10 |
| Group * Context * [Goal-state > transition revaluation] | -42.62 | -180.89 – 95.65 | -0.61 | 0.545 | 520.00 | -13.44 | -62.91 – 35.23 |
| Group * Context * [Policy > transition revaluation] | 75.40 | -51.96 – 202.77 | 1.16 | 0.245 | 520.64 | 45.10 | -2.24 – 94.04 |
| Group * Context * [Control condition > transition revaluation] | -13.41 | -120.98 – 94.17 | -0.24 | 0.807 | 520.64 | -13.63 | -62.40 – 33.21 |

*Notes*. Task condition was dummy-coded, group and context were sum-contrast-coded. The model included a by-subject random intercept and random slope for task condition. We used Satterthwaite’s degrees of freedom. As relevant violations of model assumptions were detected visually, we re-estimated the model in a robust fashion without random slope for condition due to convergence issues using the *robustlmm* package [1]. This model confirmed all significant effects based on confidence intervals (CIs) estimated using the *confintROB* package [2].

**Table P.** Parameter estimates per hybrid (reduced) random-policy SR model, group, and context.

| **Model** | **Group** | **Context** | $\boldsymbol{\alpha}$ |  | $\boldsymbol{\gamma}$ |  | $\boldsymbol{\omega}$ |  |
| --- | --- | --- | --- | --- | --- | --- | --- | --- |
|  |  |  | ***M*** | ***SE*** | ***M*** | ***SE*** | ***M*** | ***SE*** |
| Hybrid MF-reduced random-policy SR (4 goals) | Low-risk drinkers | Non-alcohol context | 0.73 | 0.02 | 0.56 | 0.01 | 0.71 | 0.02 |
|  | Low-risk drinkers | Alcohol context | 0.75 | 0.02 | 0.56 | 0.01 | 0.66 | 0.02 |
|  | High-risk drinkers | Non-alcohol context | 0.74 | 0.02 | 0.56 | 0.01 | 0.67 | 0.02 |
|  | High-risk drinkers | Alcohol context | 0.76 | 0.02 | 0.57 | 0.01 | 0.60 | 0.02 |
| Hybrid MF- random-policy SR (rigid M & w) | Low-risk drinkers | Non-alcohol context | 0.86 | 0.02 | 0.62 | 0.02 | 0.54 | 0.02 |
|  | Low-risk drinkers | Alcohol context | 0.90 | 0.01 | 0.59 | 0.02 | 0.43 | 0.02 |
|  | High-risk drinkers | Non-alcohol context | 0.86 | 0.02 | 0.61 | 0.02 | 0.49 | 0.03 |
|  | High-risk drinkers | Alcohol context | 0.87 | 0.02 | 0.62 | 0.02 | 0.39 | 0.02 |
| Hybrid MF- random-policy SR (rigid M) | Low-risk drinkers | Non-alcohol context | 0.76 | 0.02 | 0.52 | 0.01 | 0.76 | 0.02 |
|  | Low-risk drinkers | Alcohol context | 0.73 | 0.02 | 0.52 | 0.01 | 0.71 | 0.02 |
|  | High-risk drinkers | Non-alcohol context | 0.77 | 0.02 | 0.52 | 0.01 | 0.72 | 0.02 |
|  | High-risk drinkers | Alcohol context | 0.73 | 0.02 | 0.56 | 0.01 | 0.69 | 0.02 |

*Notes*. For the hybrid MF-reduced random-policy SR algorithm, goal-state-action-pairs were those pairs that led to a reward during learning plus the action from final state 10 plus the third state-action-pair on the same stage as the reward-associated state-action-pairs during learning (4 goals). MF, model-free; $M$, successor matrix; SR, successor representation; $w$, weight vector; $\alpha$, learning rate; $\gamma$, time discount factor; $\omega$, hybrid parameter weighting the influence of SR-based versus MF value estimates on choice behavior.

**Table Q.** Linear regression predicting $\omega$ from group (high-risk, low-risk drinkers) and context (alcohol, non-alcohol context) in *n*=520 participants.

|  | **Hybrid MF-reduced random-policy SR (4 goals)** | | | | **Hybrid MF-random-policy SR**  **(rigid** $\boldsymbol{M}$ **&** $\boldsymbol{w}$**)** | | | | **Hybrid MF-random-policy SR**  **(rigid** $\boldsymbol{M}$**)** | | | |  |
| --- | --- | --- | --- | --- | --- | --- | --- | --- | --- | --- | --- | --- | --- |
| **Predictors** | $\boldsymbol{\beta}$ | **95% CI** | **t-value** | ***p*** | $\boldsymbol{\beta}$ | **95% CI** | **t-value** | ***p*** | $\boldsymbol{\beta}$ | **95% CI** | **t-value** | ***p*** | ***df*** |
| Intercept | 0.66 | 0.64 – 0.68 | 62.09 | **<.001** | 0.47 | 0.44 – 0.49 | 38.20 | **<.001** | 0.72 | 0.70 – 0.74 | 75.06 | **<.001** | 516 |
| Group | -0.05 | -0.09 – -0.01 | -2.31 | .064 | -0.05 | -0.09 – 0.00 | -1.90 | .174 | -0.03 | -0.06 – -0.01 | -1.36 | .523 | 516 |
| Context | -0.06 | -0.10 – -0.02 | -2.86 | **.013** | -0.10 | -0.15 – -0.06 | -4.25 | **<.001** | -0.04 | -0.07 – 0.00 | -1.88 | .181 | 516 |
| Group * context | -0.02 | -0.10 – 0.07 | -0.38 | >1.00 | 0.01 | -0.09 – 0.11 | 0.19 | >1.00 | 0.03 | -0.05 – 0.10 | 0.76 | >1.00 | 516 |

*Notes*. Group and context were sum-contrast-coded. *P*-values were Bonferroni-corrected for three comparisons (as we considered $\omega$ according to three different models). For the hybrid MF-reduced random-policy SR algorithm, goal-state-action-pairs were those pairs that led to a reward during learning plus the action from final state 10 plus the third state-action-pair on the same stage as the reward-associated state-action-pairs during learning. For model details, see Methods. Slight assumption violations were detected visually but considered tolerable. MF, model-free; $M$, successor matrix; SR, successor representation; $w$, weight vector; $\omega$, hybrid parameter weighting the influence of SR-based versus MF value estimates on choice behavior. CI, confidence interval.

**Table R.** Linear regression predicting $\alpha$ from group (high-risk, low-risk drinkers) and context (alcohol, non-alcohol context) in *n*=520 participants.

|  | **Hybrid MF-reduced random-policy SR (4 goals)** | | | | **Hybrid MF-random-policy SR**  **(rigid** $\boldsymbol{M}$ **&** $\boldsymbol{w}$**)** | | | | **Hybrid MF-random-policy SR**  **(rigid** $\boldsymbol{M}$**)** | | | |  |
| --- | --- | --- | --- | --- | --- | --- | --- | --- | --- | --- | --- | --- | --- |
| **Predictors** | $\boldsymbol{\beta}$ | **95% CI** | **t-value** | ***p*** | $\boldsymbol{\beta}$ | **95% CI** | **t-value** | ***p*** | $\boldsymbol{\beta}$ | **95% CI** | **t-value** | ***p*** | ***df*** |
| Intercept | 0.75 | 0.73 – 0.76 | 74.38 | **<.001** | 0.87 | 0.86 – 0.89 | 104.42 | **<.001** | 0.75 | 0.73 – 0.77 | 79.21 | **<.001** | 516 |
| Group | 0.01 | -0.03 – 0.05 | 0.42 | >1.00 | -0.01 | -0.05 – 0.02 | -0.78 | >1.00 | 0.00 | -0.04 – 0.04 | 0.07 | >1.00 | 516 |
| Context | 0.02 | -0.02 – 0.06 | 1.12 | .796 | 0.02 | -0.01 – 0.05 | 1.29 | .591 | -0.03 | -0.07 – 0.01 | -1.59 | .338 | 516 |
| Group * context | 0.00 | -0.08 – 0.08 | 0.09 | >1.00 | -0.02 | -0.09 – 0.05 | -0.61 | >1.00 | -0.01 | -0.08 – 0.06 | -0.25 | >1.00 | 516 |

*Notes*. Group and context were sum-contrast-coded. *P*-values were Bonferroni-corrected for three comparisons (as we considered $\alpha$ according to three different models). For the hybrid MF-reduced random-policy SR algorithm, goal-state-action-pairs were those pairs that led to a reward during learning plus the action from final state 10 plus the third state-action-pair on the same stage as the reward-associated state-action-pairs during learning. Fo model details, see Methods. Slight assumption violations were detected visually but considered tolerable. MF, model-free; $M$, successor matrix; SR, successor representation; $w$, weight vector; $\omega$, hybrid parameter weighting the influence of SR-based versus MF value estimates on choice behavior. CI, confidence interval.

**Figure L. Relation between individual (reduced) random-policy SR use (or MB use with a higher learning rate for the reward compared to the transition structure) and questionnaire measures**. We found no significant associations between use of these strategies and any psychometric measure in any task version when applying Bonferroni correction. Individual use of (reduced) random-policy SR or MB reinforcement learning with separate learning rates for rewards and transitions was calculated as the mean over a participant’s individual slopes for the contrasts ‘reward > transition revaluation’, ‘goal-state > transition revaluation’, and ‘policy > transition’ revaluation. This average slope was then correlated with the Alcohol Use Disorder Identification Test (AUDIT) [3,4] score, the number of self-reported Alcohol Use Disorder (AUD) criteria according to the Diagnostic and Statistical Manual of Mental Disorders (DSM)-5 [5–7], the number of binge drinking days, of drinking days, and of drinks per drinking day in the past 3 months, respectively, according to a Quantity Frequency Questionnaire [8,9], subscales of the Craving Automated Scale for Alcohol (CAS-A) [10], subscales of the Urgency-Premeditation-Perseverance-Sensation Seeking-Positive Urgency (UPPS-P) scale [11,12] score, and the Obsessive Compulsive Inventory-Revised (OCI-R) [13,14] score. For *n* per plot, see Table 1. We removed implausible values from the variables “drinking days past 3 months” and “drinks per drinking day in past 3 months” (> 7 drinking days / week; > 91.245 drinking days / 3 months; > 0 drinking days in past 3 months & 0 drinks per drinking day; drinks per weekday and weekend day > drinks per drinking day in past 3 months; > 50 drinks per drinking day in past 3 months) and from “binge days in past 3 months” (> 7 binge days / week; > 91.245 binge days / 3 months). Shaded areas around regression lines depict 95% confidence intervals.

**Figure M. Rating phase results per condition, group, and context (*n*=130 participants per subplot).** The difference between expected reward per state-1-action is positive if participants expected more reward to follow the state-1-action which was suboptimal during learning (this would be correct in all revaluation conditions and incorrect in the control condition), and negative if they expected more reward to follow the state-1-action which was optimal during learning (this would be incorrect in all revaluation conditions and correct in the control condition; see Methods). Bars represent means, error bars represent standard errors of the mean.

**Table S.** Rating score difference per group, context, and task condition.

| **Group** | **Context** | **Condition** | ***M*** | ***SE*** | **95% CI lower limit** | **95% CI upper limit** |
| --- | --- | --- | --- | --- | --- | --- |
| Low-risk drinkers | Monetary version | Reward revaluation | 3.51 | 2.49 | -3.47 | 10.49 |
| Low-risk drinkers | Monetary version | Goal-state revaluation | -0.15 | 2.58 | -7.35 | 7.06 |
| Low-risk drinkers | Monetary version | Transition revaluation | -37.53 | 2.06 | -43.30 | -31.77 |
| Low-risk drinkers | Monetary version | Policy revaluation | -4.00 | 2.30 | -10.44 | 2.44 |
| Low-risk drinkers | Monetary version | Control | -47.85 | 1.60 | -52.34 | -43.37 |
| Low-risk drinkers | Alcohol version | Reward revaluation | -16.59 | 2.71 | -24.18 | -9.00 |
| Low-risk drinkers | Alcohol version | Goal-state revaluation | -18.04 | 2.74 | -25.69 | -10.38 |
| Low-risk drinkers | Alcohol version | Transition revaluation | -33.51 | 2.54 | -40.60 | -26.41 |
| Low-risk drinkers | Alcohol version | Policy revaluation | -17.27 | 2.74 | -24.94 | -9.60 |
| Low-risk drinkers | Alcohol version | Control | -53.16 | 1.88 | -58.42 | -47.90 |
| Harmful drinkers | Monetary version | Reward revaluation | -5.22 | 2.58 | -12.45 | 2.01 |
| Harmful drinkers | Monetary version | Goal-state revaluation | 3.54 | 2.79 | -4.26 | 11.34 |
| Harmful drinkers | Monetary version | Transition revaluation | -42.83 | 1.92 | -48.20 | -37.46 |
| Harmful drinkers | Monetary version | Policy revaluation | -7.96 | 2.60 | -15.25 | -0.67 |
| Harmful drinkers | Monetary version | Control | -42.82 | 1.96 | -48.31 | -37.32 |
| Harmful drinkers | Alcohol version | Reward revaluation | -24.60 | 2.97 | -32.90 | -16.30 |
| Harmful drinkers | Alcohol version | Goal-state revaluation | -19.34 | 2.67 | -26.81 | -11.87 |
| Harmful drinkers | Alcohol version | Transition revaluation | -34.55 | 2.62 | -41.88 | -27.23 |
| Harmful drinkers | Alcohol version | Policy revaluation | -29.08 | 2.64 | -36.47 | -21.69 |
| Harmful drinkers | Alcohol version | Control | -52.93 | 1.88 | -58.20 | -47.67 |

*Notes.* Rating score difference means the difference between expected reward per state-1-action. The difference is positive if participants expected more reward to follow the state-1-action which was suboptimal during learning (this would be correct in all revaluation conditions and incorrect in the control condition), and negative if they expected more reward to follow the state-1-action which was optimal during learning (this would be incorrect in all revaluation conditions and correct in the control condition; see Methods). CI, confidence interval.

**Table T.** Linear mixed-effects regression predicting rating score difference from task condition (transition revaluation as reference category) in *n*=130 low-risk drinkers performing the task in a non-alcohol context.

| **Predictor** | **Estimates** | **95% CI** | ***t-value*** | ***p*** | ***df*** |
| --- | --- | --- | --- | --- | --- |
| Intercept | -37.53 | -43.71 – -31.35 | -11.92 | **<.001** | 553.39 |
| Reward > transition revaluation | 41.04 | 33.26 – 48.82 | 10.36 | **<.001** | 520.00 |
| Goal-state > transition revaluation | 37.38 | 29.61 – 45.16 | 9.44 | **<.001** | 520.00 |
| Policy > transition revaluation | 33.53 | 25.75 – 41.31 | 8.47 | **<.001** | 520.00 |
| Control condition > transition revaluation | -10.32 | -18.10 – -2.54 | -2.61 | **0.009** | 520.00 |

*Notes.* Rating score difference means the difference between expected reward per state-1-action. The difference is positive if participants expected more reward to follow the state-1-action which was suboptimal during learning (this would be correct in all revaluation conditions and incorrect in the control condition), and negative if they expected more reward to follow the state-1-action which was optimal during learning (this would be incorrect in all revaluation conditions and correct in the control condition; see Methods). We used Satterthwaite’s degrees of freedom. CI, confidence interval.

**Table U.** Linear mixed-effects regression predicting rating score difference from task condition (policy revaluation as reference category) in *n*=130 low-risk drinkers performing the task in a non-alcohol context.

| **Predictor** | **Estimates** | **95% CI** | ***t-value*** | ***p*** | ***df*** |
| --- | --- | --- | --- | --- | --- |
| Intercept | -4.00 | -10.18 – 2.18 | -1.27 | 0.204 | 553.39 |
| Reward > policy revaluation | 7.51 | -0.27 – 15.29 | 1.90 | 0.059 | 520.00 |
| Goal-state > policy revaluation | 3.85 | -3.93 – 11.63 | 0.97 | 0.331 | 520.00 |
| Transition > policy revaluation | -33.53 | -41.31 – -25.75 | -8.47 | **<.001** | 520.00 |
| Control condition > policy revaluation | -43.85 | -51.63 – -36.07 | -11.07 | **<.001** | 520.00 |

*Notes.* Rating score difference means the difference between expected reward per state-1-action. The difference is positive if participants expected more reward to follow the state-1-action which was suboptimal during learning (this would be correct in all revaluation conditions and incorrect in the control condition), and negative if they expected more reward to follow the state-1-action which was optimal during learning (this would be incorrect in all revaluation conditions and correct in the control condition; see Methods). We used Satterthwaite’s degrees of freedom. CI, confidence interval.

**Table V.** Linear mixed-effects regression predicting rating score difference from task condition (reward revaluation as reference category) in *n*=130 low-risk drinkers performing the task in a non-alcohol context.

| **Predictor** | **Estimates** | **95% CI** | ***t-value*** | ***p*** | ***df*** |
| --- | --- | --- | --- | --- | --- |
| Intercept | 3.51 | -2.68 -9.69 | 1.11 | 0.266 | 553.39 |
| Goal-state > reward revaluation | -3.65 | -11.43 – 4.13 | -0.92 | 0.357 | 520.00 |
| Transition > reward revaluation | -41.04 | -48.82 – -33.26 | -10.36 | **<.001** | 520.00 |
| Policy > reward revaluation | -7.51 | -15.29 – 0.27 | -1.90 | 0.059 | 520.00 |
| Control condition > reward revaluation | -51.36 | -59.14 – -43.58 | -12.97 | **<.001** | 520.00 |

*Notes.* Rating score difference means the difference between expected reward per state-1-action. The difference is positive if participants expected more reward to follow the state-1-action which was suboptimal during learning (this would be correct in all revaluation conditions and incorrect in the control condition), and negative if they expected more reward to follow the state-1-action which was optimal during learning (this would be incorrect in all revaluation conditions and correct in the control condition; see Methods). We used Satterthwaite’s degrees of freedom. CI, confidence interval.

**Table W.** Linear mixed-effects regression predicting rating score difference from task condition (control condition as reference category) in *n*=130 low-risk drinkers performing the task in a non-alcohol context.

| **Predictor** | **Estimates** | **95% CI** | ***t-value*** | ***p*** | ***df*** |
| --- | --- | --- | --- | --- | --- |
| Intercept | -47.85 | -54.04 – -41.67 | -15.20 | **<.001** | 553.39 |
| Reward revaluation > control condition | 51.36 | 43.58 – 59.14 | 12.97 | **<.001** | 520.00 |
| Goal-state revaluation > control condition | 47.71 | 39.93 – 55.49 | 12.05 | **<.001** | 520.00 |
| Transition revaluation > control condition | 10.32 | 2.54 – 18.10 | 2.61 | **0.009** | 520.00 |
| Policy revaluation > control condition | 43.85 | 36.07 – 51.63 | 11.07 | **<.001** | 520.00 |

*Notes.* Rating score difference means the difference between expected reward per state-1-action. The difference is positive if participants expected more reward to follow the state-1-action which was suboptimal during learning (this would be correct in all revaluation conditions and incorrect in the control condition), and negative if they expected more reward to follow the state-1-action which was optimal during learning (this would be incorrect in all revaluation conditions and correct in the control condition; see Methods). We used Satterthwaite’s degrees of freedom. CI, confidence interval.

**Table X.** Linear mixed-effects regression predicting rating score difference from task condition (transition revaluation as reference category), group (high-risk, low-risk drinkers) and context (alcohol, non-alcohol context) in *n*=520 participants.

| **Predictor** | **Estimates** | **95% CI** | ***t-value*** | ***p*** | ***df*** |
| --- | --- | --- | --- | --- | --- |
| Intercept | -37.11 | -40.48 – -33.73 | -21.56 | **<.001** | 2054.78 |
| Group | -3.17 | -9,92 – 3.58 | -0.92 | 0.357 | 2054.78 |
| Context | 6.15 | -0.60 – 12.90 | 1.79 | 0.074 | 2054.78 |
| Reward > transition revaluation | 26.38 | 22.27 – 30.49 | 12.58 | **<.001** | 2080.00 |
| Goal-state > transition revaluation | 28.61 | 24.50 – 32.72 | 13.64 | **<.001** | 2080.00 |
| Policy > transition revaluation | 22.53 | 18.41 – 26.64 | 10.74 | **<.001** | 2080.00 |
| Control condition > transition revaluation | -12.08 | -16.20 – -7.97 | -5.76 | **<.001** | 2080.00 |
| Group * Context | 4.25 | -9.24 – 17.75 | 0.62 | 0.537 | 2054.78 |
| Group * [Reward > transition revaluation] | -5.19 | -13.42 – 3.03 | -1.24 | 0.216 | 2080.00 |
| Group * [Goal-state > transition revaluation] | 4.37 | -3.86 – 12.59 | 1.04 | 0.298 | 2080.00 |
| Group * [Policy > transition revaluation] | -4.72 | -12.94 – 3.51 | -1.12 | 0.261 | 2080.00 |
| Group * [Control condition > transition revaluation] | 5.81 | -2.42 – 14.03 | 1.38 | 0.166 | 2080.00 |
| Context * [Reward > transition revaluation] | -25.89 | -24.12 – -17.67 | -6.17 | **<.001** | 2080.00 |
| Context * [Goal-state > transition revaluation] | -26.53 | -34.76 – -18.31 | -6.33 | **<.001** | 2080.00 |
| Context * [Policy > transition revaluation] | -23.35 | -31.57 – -15.12 | -5.57 | **<.001** | 2080.00 |
| Context * [Control condition > transition revaluation] | -13.86 | -22.09 – -5.64 | -3.31 | **0.001** | 2080.00 |
| Group * Context * [Reward > transition revaluation] | -3.54 | -19.99 – -12.91 | -0.42 | 0.673 | 2080.00 |
| Group * Context * [Goal-state > transition revaluation] | -9.27 | -25.69 – 7.21 | -1.10 | 0.271 | 2080.00 |
| Group * Context * [Policy > transition revaluation] | -12.11 | -28.56 – 4.34 | -1.44 | 0.149 | 2080.00 |
| Group * Context * [Control condition > transition revaluation] | -9.06 | -25.51 – 7.39 | -1.08 | 0.280 | 2080.00 |

*Notes.* Rating score difference means the difference between expected reward per state-1-action. The difference is positive if participants expected more reward to follow the state-1-action which was suboptimal during learning (this would be correct in all revaluation conditions and incorrect in the control condition), and negative if they expected more reward to follow the state-1-action which was optimal during learning (this would be incorrect in all revaluation conditions and correct in the control condition; see Methods). We used Satterthwaite’s degrees of freedom. Bonferroni-corrected post-hoc tests for the interaction between condition-contrasts and context showed that contrasts were significant in both the alcohol (‘reward>transition revaluation’: $\beta$=13.43, *t*(2096)=4.51, *p*<.001; ‘goal-state>transition revaluation’: $\beta$=15.34, *t*(2096)=5.15, *p*<.001; ‘policy>transition revaluation’: $\beta$=10.85, *t*(2096)=3.64, *p*=.001) and non-alcohol context (‘reward>transition revaluation’: $\beta$=39.33, *t*(2096)=13.21, *p*<.001; ‘goal-state>transition revaluation’: $\beta$=41.88, *t*(2096)=14.07, *p*<.001; ‘policy>transition revaluation’: $\beta$=34.20, *t*(2096)=11.49, *p*<.001), except the contrast ‘control condition>transition revaluation which was only significant in the alcohol ($\beta$=-19.02, *t*(2096)=-6.39, *p*<.001) but not the non-alcohol version ($\beta$=-5.15, *t*(2096)=-1.73, *p*=.334). Slight assumption violations were detected visually but considered tolerable. CI, confidence interval.

**Table Y.** Linear mixed-effects regression predicting rating score difference from task condition (control condition as reference category), group (high-risk, low-risk drinkers) and context (alcohol, non-alcohol context) in *n*=520 participants.

| **Predictor** | **Estimates** | **95% CI** | ***t-value*** | ***p*** | ***df*** |
| --- | --- | --- | --- | --- | --- |
| Intercept | -49.19 | -52.57 – -45.82 | -28.59 | **<.001** | 2054.78 |
| Group | 2.63 | -4.11 – 9.38 | 0.77 | 0.444 | 2054.78 |
| Context | -7.71 | -14.46 – -0.96 | -2.24 | **0.025** | 2054.78 |
| Reward revaluation > control condition | 38.47 | 34.35 – 42.58 | 18.34 | **<.001** | 2080.00 |
| Goal-state revaluation > control condition | 40.69 | 36.58 – 44.81 | 19.41 | **<.001** | 2080.00 |
| Transition revaluation > control condition | 12.08 | 7.97 – 16.20 | 5.76 | **<.001** | 2080.00 |
| Policy revaluation > control condition | 34.61 | 30.50 – 38.72 | 16.51 | **<.001** | 2080.00 |
| Group * Context | -4.81 | -18.31 – 8.69 | -0.70 | 0.485 | 2054.78 |
| Group * [Reward revaluation > control condition] | -11.00 | -19.22 – -2.78 | -2.62 | **0.009** | 2080.00 |
| Group * [Goal-state revaluation > control condition] | -1.44 | -9.67 – 6.78 | -0.34 | 0.731 | 2080.00 |
| Group * [Transition revaluation > control condition] | -5.81 | -14.03 – 2.42 | -1.38 | 0.166 | 2080.00 |
| Group * [Policy revaluation > control condition] | -10.52 | -18.75 – -2.30 | -2.51 | **0.012** | 2080.00 |
| Context * [Reward revaluation > control condition] | -12.03 | -20.26 – -2.81 | -2.87 | **0.004** | 2080.00 |
| Context * [Goal-state revaluation > control condition] | -12.67 | -20.90 – -4.45 | -3.02 | **0.003** | 2080.00 |
| Context * [Transition revaluation > control condition] | 13.86 | 5.64 – 22.09 | 3.31 | **0.001** | 2080.00 |
| Context * [Policy revaluation > control condition] | -9.48 | -17.71 – -1.26 | -2.26 | **0.024** | 2080.00 |
| Group * Context * [Reward revaluation > control condition] | 5.52 | -10.93 – 21.97 | 0.66 | 0.510 | 2080.00 |
| Group * Context * [Goal-state revaluation > control condition] | -0.18 | -16.63 – 16.27 | -0.02 | 0.983 | 2080.00 |
| Group * Context * [Transition revaluation > control condition] | 9.06 | -7.39 – 25.51 | 1.08 | 0.280 | 2080.00 |
| Group * Context * [Policy revaluation > control condition] | -3.05 | -19.50 – 13.40 | -0.36 | 0.717 | 2080.00 |

*Notes.* Rating score difference means the difference between expected reward per state-1-action. The difference is positive if participants expected more reward to follow the state-1-action which was suboptimal during learning (this would be correct in all revaluation conditions and incorrect in the control condition), and negative if they expected more reward to follow the state-1-action which was optimal during learning (this would be incorrect in all revaluation conditions and correct in the control condition; see Methods). We used Satterthwaite’s degrees of freedom. Bonferroni-corrected post-hoc tests for significant interactions showed that the contrasts ‘reward revaluation>control condition’ (high-risk drinkers: $\beta$=33.00, *t*(2096)=11.07, *p*<.001; low-risk drinkers: $\beta$=44.00, *t*(2096)=14.78, *p*<.001) and ‘policy revaluation>control condition’ (high-risk drinkers: $\beta$=29.04, *t*(2096)=9.86, *p*<.001; low-risk drinkers: $\beta$=39.90, *t*(2096)=14.77, *p*<.001) were significant in both groups. Moreover, the contrasts ‘reward revaluation>control condition’ (alcohol context: $\beta$=32.45, *t*(2096)=10.90, *p*<.001; non-alcohol context: $\beta$=44.48, *t*(2096)=14.94, *p*<.001), ‘goal-state revaluation>control condition’ (alcohol context: $\beta$=34.36, *t*(2096)=11.54, *p*<.001; non-alcohol context: $\beta$=47.03, *t*(2096)=15.80, *p*<.001), and ‘policy revaluation>control condition’ (alcohol context: $\beta$=29.87, *t*(2096)=10.03, *p*<.001; non-alcohol context: $\beta$=39.35, *t*(2096)=13.22, *p*<.001) were significant in both contexts, but the contrast ‘transition revaluation>control condition’ (alcohol context: $\beta$=19.02, *t*(2096)=6.39, *p*<.001; non-alcohol context: $\beta$=5.15, *t*(2096)=1.73, *p*=.084) was only significant in the alcohol context. Slight assumption violations were detected visually but considered tolerable. CI, confidence interval.

**Table Z.** Linear mixed-effects regression predicting rating score difference from task condition (reward revaluation as reference category) and group (high-risk, low-risk drinkers) in *n*=260 participants performing the task in an alcohol context.

| **Predictor** | **Estimates** | **95% CI** | ***t-value*** | ***p*** | ***df*** |
| --- | --- | --- | --- | --- | --- |
| Intercept | -20.60 | -25.60 – -15.59 | -8.07 | **<.001** | 1009.13 |
| Goal-state > reward revaluation | 1.91 | -4.15 – 7.96 | 0.62 | 0.537 | 1040.00 |
| Transition > reward revaluation | -13.43 | -19.49 – -7.38 | -4.35 | **<.001** | 1040.00 |
| Policy > reward revaluation | -2.58 | -8.64 – 3.48 | -0.84 | 0.403 | 1040.00 |
| Control condition > reward revaluation | -32.45 | -38.51 – -26.39 | -10.51 | **<.001** | 1040.00 |
| Group | -8.01 | -18.02 – 2.01 | -1.57 | 0.117 | 1009.13 |
| [Goal-state > reward revaluation] * Group | 6.71 | -5.41 – 18.82 | 1.09 | 0.277 | 1040.00 |
| [Transition > reward revaluation] * Group | 6.96 | -5.15 – 19.08 | 1.13 | 0.260 | 1040.00 |
| [Policy > reward revaluation] * Group | -3.81 | -15.92 – 8.31 | -0.62 | 0.538 | 1040.00 |
| [Control condition > reward revaluation] * Group | 8.24 | -3.88 – 20.35 | 1.33 | 0.182 | 1040.00 |

*Notes.* Rating score difference means the difference between expected reward per state-1-action. The difference is positive if participants expected more reward to follow the state-1-action which was suboptimal during learning (this would be correct in all revaluation conditions and incorrect in the control condition), and negative if they expected more reward to follow the state-1-action which was optimal during learning (this would be incorrect in all revaluation conditions and correct in the control condition; see Methods). We used Satterthwaite’s degrees of freedom. Slight assumption violations were detected visually but considered tolerable. CI, confidence interval.

**Table AA.** Linear mixed-effects regression predicting rating score difference from task condition (reward revaluation as reference category), group (high-risk, low-risk drinkers) and context (alcohol, non-alcohol context) in *n*=520 participants.

| **Predictor** | **Estimates** | **95% CI** | **t-value** | ***p*** | ***df*** |
| --- | --- | --- | --- | --- | --- |
| Intercept | -10.72 | -14.10 – -7.35 | -6.23 | **<.001** | 2054.78 |
| Group | -8.37 | -15.11 – -1.62 | -2.43 | **0.015** | 2054.78 |
| Context | -19.74 | -26.49 – -12.99 | -5.74 | **<.001** | 2054.78 |
| Goal-state > reward revaluation | 2.23 | -1.88 – 6.34 | 1.06 | 0.288 | 2080.00 |
| Transition > reward revaluation | -26.38 | -30.49 – -22-27 | -12.58 | **<.001** | 2080.00 |
| Policy > reward revaluation | -3.85 | -7.97 – 0.26 | -1.84 | 0.066 | 2080.00 |
| Control condition > reward revaluation | -38.47 | -42.58 – -34.35 | -18.34 | **<.001** | 2080.00 |
| Group * Context | 0.72 | -12.78 – 14.21 | 0.10 | 0.917 | 2054.78 |
| Group * [Goal-state > reward revaluation] | 9.56 | 1.33 – 17.78 | 2.28 | **0.023** | 2080.00 |
| Group * [Transition > reward revaluation] | 5.19 | -3.03 – 13.42 | 1.24 | 0.216 | 2080.00 |
| Group * [Policy > reward revaluation] | 0.48 | -7.75 – 8.70 | 0.11 | 0.909 | 2080.00 |
| Group * [Control condition > reward revaluation] | 11.00 | 2.78 – 19.22 | 2.62 | **0.009** | 2080.00 |
| Context * [Goal-state > reward revaluation] | -0.64 | -8.87 – 7.58 | -0.15 | 0.878 | 2080.00 |
| Context * [Transition > reward revaluation] | 25.89 | 17.67 – 34.12 | 6.17 | **<.001** | 2080.00 |
| Context * [Policy > reward revaluation] | 2.55 | -5.68 – 10.77 | 0.61 | 0.544 | 2080.00 |
| Context * [Control condition > reward revaluation] | 12.03 | 3.81 – 20.26 | 2.87 | **0.004** | 2080.00 |
| Group * Context * [Goal-state > reward revaluation] | -5.70 | -22.15 – 10.75 | -0.68 | 0.497 | 2080.00 |
| Group * Context * [Transition > reward revaluation] | 2.54 | -12.91 – 19.99 | 0.42 | 0.673 | 2080.00 |
| Group * Context * [Policy > reward revaluation] | -8.57 | -25.02 – 7.88 | -1.02 | 0.307 | 2080.00 |
| Group * Context * [Control condition > reward revaluation] | -5.52 | -21.97 – 10.93 | -0.66 | 0.510 | 2080.00 |

*Notes.* Rating score difference means the difference between expected reward per state-1-action. The difference is positive if participants expected more reward to follow the state-1-action which was suboptimal during learning (this would be correct in all revaluation conditions and incorrect in the control condition), and negative if they expected more reward to follow the state-1-action which was optimal during learning (this would be incorrect in all revaluation conditions and correct in the control condition; see Methods). We used Satterthwaite’s degrees of freedom. Bonferroni-corrected post-hoc tests for significant interactions revealed that the contrast ‘goal-state>reward revaluation’ was significant in high-risk ($\beta$=7.01, *t*(2096)=2.35, *p*=.037) but not in low-risk drinkers ($\beta$=-2.55, *t*(2096)=-0.86, *p*=.784), whereas the contrast ‘control condition>reward revaluation’ was significant in both high-risk ($\beta$=-32.97, *t*(2096)=-11.07, *p*<.001) and low-risk drinkers ($\beta$=-43.97, *t*(2096)=-14.77, *p*<.001). Moreover, the contrasts ‘transition>reward revaluation’ (alcohol context: $\beta$=-13.40, *t*(2096)=-4.51, *p*<.001; non-alcohol context: $\beta$=39.30, *t*(2096)=-13.21, *p*<.001) and ‘control condition>reward revaluation’ (alcohol context: $\beta$=32.50, *t*(2096)=-10.90, *p*<.001; non-alcohol context: $\beta$=-44.50, *t*(2096)=-14.94, *p*<.001) were significant in both contexts. Slight assumption violations were detected visually but considered tolerable. CI, confidence interval.

# Exploring alternative explanations and confounding factors

## Differences in learning, motivation, or random behavior between conditions and contexts

A potential explanation for revaluation performance differences is comparatively impaired learning in transition revaluation and the alcohol context. This is unlikely as we excluded data from participants who failed to select the optimal path in less than three out of the last five learning trials. Logistic mixed-effects regression verified that the probability of selecting the optimal path in the last five learning trials did not differ between the control condition and any other condition, nor between contexts or groups (Table K; *lme4* code: optimal_path ~ group*version*condition + (1 + condition | participantID). Another possibility is that participants behaved more randomly in the alcohol compared to non-alcohol context after changes were introduced in the environment, e.g. due to lower attentional engagement, lower motivation, higher cognitive load, higher inattentiveness, or more pronounced forgetting, thus leading to lower revaluation performance. While response times in reward revaluation test trials were higher in the alcohol than the non-alcohol context according to a robust linear mixed-effects regression (*OR*=37.79, robust 95%CI: [23.91–59.14]; different from standard linear mixed-effects regression results in Table L), this was not reflected in random behavior as the probability of random path preference changes in the control condition was not higher in alcohol compared to non-alcohol contexts (*OR*=1.07, *z*=0.13, *p*=.894; Table J). Lower attentiveness or motivation, higher cognitive load, and more pronounced forgetting or random behavior are thus unlikely alternative explanations for the observed context effects on condition differences in revaluation performance.

## Group-level vs. individual strategy signatures

The signatures of (reduced) random-policy SR use observed on the group level are not necessarily driven by participants who exhibit these signatures. Specifically, some individuals might be successful only in reward revaluation, others only in policy revaluation, and while none of them show signatures of (reduced) random-policy SR use, together, they would create the observed pattern. While we did see substantial variation in revaluation performance per condition (Figure N), individual slopes for the contrasts ‘policy > transition revaluation’ and ‘reward > transition revaluation’ ($\rho$=.71, p<.001) or ‘goal-state > transition revaluation’ ($\rho$=.78, p<.001) were highly correlated, respectively, indicating that the group-level pattern was driven by individuals who themselves showed signatures of (reduced) random-policy SR.

## Strategy changes between revaluation conditions

The differences in revaluation performance may reflect shifts in strategy use over the course of the task, potentially mirrored by response times (Figure J, Table G). Linear mixed-effects regression (*lme4* code: response_time ~ group*context*condition + (1 + condition | participantID)) indeed revealed that, averaged over groups and contexts, response times in test trial states (states 1-3) in transition revaluation (*M*$\pm$*SE*=551.97$\pm$8.95ms) were lower compared to reward (*M*$\pm$*SE*=599.10$\pm$8.92ms; $\beta$=47.13, *t*(520.00)=2.99, *p*=.003), goal-state (*M*$\pm$*SE*=612.91$\pm$9.46ms; $\beta$=60.94, *t*(520.00)=3.46, *p*=.001) and policy revaluation (*M*$\pm$*SE*=615.92$\pm$9.60ms; $\beta$=63.95, *t*(520.64)=3.95, *p*<.001), but higher than in the control condition (*M*$\pm$*SE*=513.11$\pm$8.07ms; $\beta$=-38.86, *t*(520.03)=-2.84, *p*=.005; Table O). Response times in reward revaluation did not differ from those in goal-state ($\beta$=13.81, *t*(520.00)=0.74, *p*=.460) or policy revaluation ($\beta$=16.82, *t*(519.96)=0.93, *p*=.350; Table L).

This stands in contrast to Momennejad and colleagues [15], who reported higher response times in transition compared to reward revaluation in a passive revaluation task. In their experiment, however, performance in transition revaluation was much higher than in our data, in line with a general speed-accuracy trade-off. The authors suggested that transition revaluation favored a computationally costly MB strategy [15] which implies a change in strategy contributions between conditions. Our results instead suggest that revaluation comes at a cost per se, which is why response times in all revaluation conditions were higher than in the control condition. If a (reduced) random-policy SR strategy is used, computations in transition revaluation might be faster as neither the reward vector nor the rows of $M^{random}$ corresponding to states 1-3 changed here, leading to larger differences between value estimates and less ambiguity [16]. On the other hand, faster response times in transition revaluation would be in line with a suggestion that higher state prediction errors favor MF use [15,17,18]. However, this account also predicts more MB contributions, and thus better performance, in goal-state and policy revaluation due to higher reward prediction errors compared to reward revaluation, which we did not observe in our data [15,18]. In conclusion, while we cannot fully rule out strategy changes between task conditions, we also find no strong evidence for them.

## Strategy changes within revaluation conditions

Participants performed five test trials per condition, which allowed us to test whether strategy signatures changed over the course of the test phase (Figure O). We repeated the logistic mixed-effect regression in *N*=520 participants with trial number and its interactions with condition, group, and context as additional fixed effects (*lme4* code: change_path ~ group*version*condition*trial + (1 + condition | participantID)). Test trial number was a continuous predictor ranging from 0-4, such that the intercept and predictors not including an interaction with test trial number could be meaningfully interpreted as referring to the first trial.

Across groups and conditions, the contrasts between transition revaluation and reward (*OR*=0.80, *z*=-3.06, *p*=.002), goal-state (*OR*=0.76, *z*=-3.73, *p*<.001), and policy revaluation performance (*OR*=0.65, *z*=-5.75, *p*<.001), respectively, decreased over the course of test trials, whereas transition revaluation performance did not change (*OR*=1.12, *z*=1.94, *p*=.053; Table AB). We found no significant interactions of these effects with group, context, or both (Table AB). While this might suggest less contributions of (reduced) random-policy SR or MB with separate learning rates for the reward and transition structure and larger MF contributions in later test trials, it might also indicate increased exploration. This interpretation is supported by an increase in the proportion of erroneous path preference changes in the control condition over the course of the test phase (*OR*=1.33, *z*=4.38, *p*<.001), which, as indicated by an interaction with group, was present at least in low-risk ($\beta$=0.50, *z*=5.05, *p*<.001), but not in high-risk drinkers ($\beta$=0.08, *z*=0.90, *p*=.368; Table AC). As no reward feedback was given during test trials, decreases in contrasts might also be due to unlearning what had been learned during re-learning. This might particularly affect revaluation conditions, as value differences between optimal and suboptimal paths established during only nine re-learning trials are likely smaller than in the control condition, where value differences have been consistently established over 33 trials. The fact that we find signatures of (reduced) random-policy SR use (or MB with separate learning rates for the reward and transition structure) even when considering all test trials and in rating phase data speaks for the robustness of our findings.

**Figure N. Interindividual variation in revaluation performance per condition, group, and context (*n*=130 participants per subplot).** Number of participants with a certain observed proportion of test trials in which they changed their path preference, compared to the optimal path during learning (see Methods).

**Figure O. Revaluation performance per test trial, group, and context (*n*=130 participants per subplot).** Observed proportion of trials per test trial number, condition, group, and context in which participants changed their path preference, compared to the optimal path during learning (see Methods). Bars represent means, error bars represent standard errors of the mean.

**Table AB.** Logistic mixed-effects regression predicting revaluation performance from task condition (transition revaluation as reference category), group (high-risk, low-risk drinkers), context (alcohol, non-alcohol context), and trial number in *n*=520 participants.

| **Predictor** | **Odds ratio** | **95% CI** | ***z-value*** | ***p*** |
| --- | --- | --- | --- | --- |
| Intercept | 0.01 | 0.01 – 0.02 | -11.11 | **<.001** |
| Group | 0.65 | 0.28 – 1.53 | -0.98 | 0.328 |
| Context | 3.67 | 1.56 – 8.64 | 2.97 | **0.003** |
| Reward > transition revaluation | 41.97 | 17.81 – 98.92 | 8.54 | **<.001** |
| Goal-state > transition revaluation | 43.86 | 18.56 – 103.66 | 8.62 | **<.001** |
| Policy > transition revaluation | 47.83 | 20.51 – 111.56 | 8.95 | **<.001** |
| Control condition > transition revaluation | 0.03 | 0.01 – 0.13 | -4.98 | **<.001** |
| Trial | 1.12 | 1.00 – 1.26 | 1.94 | 0.053 |
| Group * Context | 0.35 | 0.06 – 1.95 | -1.20 | 0.232 |
| Group * [Reward > transition revaluation] | 0.39 | 0.13 – 1.15 | -1.70 | 0.089 |
| Group * [Goal-state > transition revaluation] | 1.13 | 0.38 – 3.35 | 0.23 | 0.820 |
| Group * [Policy > transition revaluation] | 0.60 | 0.21 – 1.71 | -0.95 | 0.343 |
| Group * [Control condition > transition revaluation] | 6.14 | 1.44 – 26.14 | 2.46 | **0.104** |
| Context * [Reward > transition revaluation] | 0.07 | 0.03 – 0.22 | -4.72 | **<.001** |
| Context * [Goal-state > transition revaluation] | 0.09 | 0.03 -0.26 | -4.34 | **<.001** |
| Context * [Policy > transition revaluation] | 0.05 | 0.02 – 0.14 | -5.57 | **<.001** |
| Context * [Control condition > transition revaluation] | 0.20 | 0.05 – 0.85 | -2.18 | **0.029** |
| Group * Trial | 0.98 | 0.77 – 1.23 | -0.21 | 0.836 |
| Context * Trial | 0.87 | 0.69 – 1.10 | -1.20 | 0.232 |
| [Reward > transition revaluation] * Trial | 0.80 | 0.69 – 0.92 | -3.06 | **0.002** |
| [Goal-state > transition revaluation] * Trial | 0.76 | 0.65 – 0.88 | -3.73 | **<.001** |
| [Policy > transition revaluation] * Trial | 0.65 | 0.56 – 0.75 | -5.75 | **<.001** |
| [Control condition > transition revaluation] * Trial | 1.19 | 1.00 – 1.41 | 1.93 | 0.054 |
| Group * Context * [Reward > transition revaluation] | 2.48 | 0.29 – 21.22 | 0.83 | 0.406 |
| Group * Context * [Goal-state > transition revaluation] | 2.84 | 0.32 – 25.13 | 0.94 | 0.347 |
| Group * Context * [Policy > transition revaluation] | 1.18 | 0.15 – 0.47 | 0.15 | 0.879 |
| Group * Context * [Control condition > transition revaluation] | 1.15 | 0.06 – 21.11 | 0.10 | 0.924 |
| Group * Context * Trial | 1.42 | 0.89 – 2.27 | 1.48 | 0.140 |
| Group * [Reward > transition revaluation] * Trial | 1.11 | 0.83 – 1.49 | 0.72 | 0.472 |
| Group * [Goal-state > transition revaluation] * Trial | 1.11 | 0.83 – 1.49 | 0.72 | 0.475 |
| Group * [Policy > transition revaluation] * Trial | 0.99 | 0.74 – 1.34 | -0.04 | 0.970 |
| Group * [Control condition > transition revaluation] * Trial | 0.67 | 0.48 – 0.95 | -2.23 | **0.026** |
| Context * [Reward > transition revaluation] * Trial | 1.23 | 0.92 – 1.65 | 1.39 | 0.163 |
| Context * [Goal-state > transition revaluation] * Trial | 0.95 | 0.71 – 1.28 | -0.31 | 0.753 |
| Context * [Policy > transition revaluation] * Trial | 1.24 | 0.92 – 1.66 | 1.41 | 0.158 |
| Context * [Control condition > transition revaluation] * Trial | 1.36 | 0.96 – 1.92 | 1.74 | 0.081 |
| Group * Context * [Reward > transition revaluation] * Trial | 0.71 | 0.40 – 1.28 | -1.13 | 0.257 |
| Group * Context * [Goal-state > transition revaluation] * Trial | 0.56 | 0.31 – 1.00 | -1.95 | 0.052 |
| Group * Context * [Policy > transition revaluation] * Trial | 0.69 | 0.38 – 1.24 | -1.24 | 0.215 |
| Group * Context * [Control condition > transition revaluation] * Trial | 0.81 | 0.40 – 1.16 | -0.60 | 0.546 |

*Notes*. Revaluation performance is quantified as the probability of changing path preference compared to the optimal path during learning on a given test trial (see Methods). Task condition was dummy-coded, group and context were sum-contrast-coded. The model included a by-subject random intercept and random slope for task condition. When focusing only on first test trials, we found all effects observed when considering all test trials: performance was lower in transition revaluation compared to reward, goal-state, and policy revaluation, and significant interactions with context revealed that these effects were smaller in the alcohol context ([reward>transition revaluation]: *OR*=11.45, *z*=4.95, *p*<.001*;* ([goal-state>transition revaluation]: *OR*=13.03, *z*=5.20, *p*<.001*;* ([policy>transition revaluation]: *OR*=10.73, *z*=4.90, *p*<.001) compared to the non-alcohol context ([reward>transition revaluation]: *OR*=153.83, *z*=9.32, *p*<.001; [goal-state>transition revaluation]: *OR*=147.61, *z*=9.15, *p*<.001; [policy>transition revaluation]: *OR*=213.21, *z*=10.08, *p*<.001). Besides that, the contrast ‘control condition>transition revaluation’ was more negative in the alcohol context (*OR*=0.02, *z*=-5.50, *p*<.001) compared to the non-alcohol context (*OR*=0.08, *z*=-3.27, *p*=.004) and in low-risk- (*OR*=0.01, *z*=-5.41, *p*<.001) compared to high-risk drinkers (*OR*=0.08, *z*=-3.28, *p*=.001) in the first test trial, respectively, and decreased over time in low-risk drinkers ($\beta$=0.37, *z*=2.87, *p*=.004), but not in high-risk drinkers ($\beta$=-0.03, *z*=-0.21, *p*=.834). Effects not mentioned here are interpreted in the main text. Bonferroni-corrected post-hoc tests were computed using estimated marginal means for significant interactions. CI, confidence interval; z-value, Wald test z-value.

**Table AC.** Logistic mixed-effects regression predicting revaluation performance from task condition (control condition as reference category), group (high-risk, low-risk drinkers), context (alcohol, non-alcohol context), and trial number in *n*=520 participants.

| **Predictor** | **Odds ratio** | **95% CI** | ***z-value*** | ***p*** |
| --- | --- | --- | --- | --- |
| Intercept | 0.00 | 0.00 – 0.00 | -14.53 | **<.001** |
| Group | 4.02 | 1.26 – 12.78 | 2.36 | **0.018** |
| Context | 0.73 | 0.23 – 2.32 | -0.53 | 0.595 |
| Reward revaluation > control condition | 1229.29 | 394.30 – 3832.56 | 12.26 | **<.001** |
| Goal-state revaluation > control condition | 1282.66 | 417.14 – 3956.37 | 12.47 | **<.001** |
| Transition revaluation > control condition | 29.29 | 7.77 – 110.44 | 4.99 | **<.001** |
| Policy revaluation > Control condition | 1400.97 | 451.58 – 4346.35 | 12.54 | **<.001** |
| Trial | 1.33 | 1.17 – 1.51 | 4.38 | **<.001** |
| Group * Context | 0.41 | 0.04 – 3.97 | -0.78 | 0.438 |
| Group * [Reward revaluation > control condition] | 0.06 | 0.02 – 0.27 | -3.79 | **<.001** |
| Group * [Goal-state revaluation > control condition] | 0.18 | 0.05 – 0.73 | -2.41 | **0.016** |
| Group * [Transition revaluation > control condition] | 0.16 | 0.04 – 0.69 | -2.46 | **0.014** |
| Group * [Policy revaluation > Control condition] | 0.10 | 0.02 – 0.40 | -3.24 | **0.001** |
| Context * [Reward revaluation > control condition] | 0.37 | 0.09 – 1.55 | -1.36 | 0.174 |
| Context * [Goal-state revaluation > control condition] | 0.44 | 0.11 – 1.76 | -1.16 | 0.247 |
| Context * [Transition revaluation > control condition] | 5.02 | 1.18 – 21.36 | 2.18 | **0.029** |
| Context * [Policy revaluation > Control condition] | 0.25 | 0.06 – 1.03 | -1.91 | 0.056 |
| Group * Trial | 0.66 | 0.51 – 0.85 | -3.21 | **0.001** |
| Context * Trial | 1.18 | 0.91 – 1.52 | 1.27 | 0.204 |
| [Reward revaluation > control condition] * Trial | 0.67 | 0.57 – 0.78 | -5.04 | **<.001** |
| [Goal-state revaluation > control condition] * Trial | 0.64 | 0.55 – 0.75 | -5.67 | **<.001** |
| [Transition revaluation > control condition] * Trial | 0.84 | 0.71 – 1.00 | -1.93 | 0.054 |
| [Policy revaluation > Control condition] * Trial | 0.54 | 0.46 – 0.64 | -7.54 | **<.001** |
| Group * Context * [Reward revaluation > control condition] | 2.15 | 0.13 – 35.09 | 0.54 | 0.591 |
| Group * Context * [Goal-state revaluation > control condition] | 2.46 | 0.16 – 37.19 | 0.65 | 0.516 |
| Group * Context * [Transition revaluation > control condition] | 0.86 | 0.05 – 15.10 | -0.10 | 0.920 |
| Group * Context * [Policy revaluation > Control condition] | 1.02 | 0.06 – 16.20 | 0.01 | 0.990 |
| Group * Context * Trial | 1.15 | 0.69 – 1.91 | 0.54 | 0.591 |
| Group * [Reward revaluation > control condition] * Trial | 1.65 | 1.21 – 2.25 | 3.17 | **0.002** |
| Group * [Goal-state revaluation > control condition] * Trial | 1.65 | 1.21 – 2.24 | 3.17 | **0.002** |
| Group * [Transition revaluation > control condition] * Trial | 1.48 | 1.05 – 2.09 | 2.23 | **0.026** |
| Group * [Policy revaluation > Control condition] * Trial | 1.47 | 1.08 – 2.02 | 2.43 | **0.015** |
| Context * [Reward revaluation > control condition] * Trial | 0.90 | 0.66 – 1.23 | -0.64 | 0.525 |
| Context * [Goal-state revaluation > control condition] * Trial | 0.70 | 0.52 – 0.95 | -2.25 | **0.024** |
| Context * [Transition revaluation > control condition] * Trial | 0.74 | 0.52 – 1.04 | -1.75 | 0.081 |
| Context * [Policy revaluation > Control condition] * Trial | 0.91 | 0.67 – 1.24 | -0.60 | 0.551 |
| Group * Context * [Reward revaluation > control condition] * Trial | 0.88 | 0.48 – 1.63 | -0.40 | 0.692 |
| Group * Context * [Goal-state revaluation > control condition] * Trial | 0.69 | 0.38 – 1.28 | -1.17 | 0.244 |
| Group * Context * [Transition revaluation > control condition] * Trial | 1.24 | 0.62 – 2.47 | 0.61 | 0.543 |
| Group * Context * [Policy revaluation > Control condition] * Trial | 0.85 | 0.46 – 1.59 | -0.51 | 0.613 |

*Notes*. Revaluation performance is quantified as the probability of changing path preference compared to the optimal path during learning on a given test trial (see Methods). Task condition was dummy-coded, group and context were sum-contrast-coded. The model included a by-subject random intercept and random slope for task condition. We performed Bonferroni-corrected post-hoc tests besides the ones reported in the main text using estimated marginal means for significant interactions, revealing that, in the first trial, low-risk drinkers exhibited larger contrasts ‘reward revaluation>control condition’ (OR=4858.41, *z*=12.04, *p*<.001), ‘goal-state revaluation>control condition’ (OR=2989.72, *z*=11.50, *p*<.001), ‘transition revaluation>control condition’ (OR=72.58, *z*=5.42, *p*<.001), and ‘policy revaluation>control condition’ (OR=4465.91, *z*=11.98, *p*<.001) compared to high-risk drinkers (‘reward revaluation>control condition’: OR=311.04, *z*=8.66, *p*<.001; ‘goal-state revaluation>control condition’: OR=552.01, *z*=9.73, *p*<.001; ‘transition revaluation>control condition’: OR=11.82, *z*=3.28, *p*=.004; ‘policy revaluation>control condition’: OR=439.49, *z*=9.27, *p*<.001). In the first test trial, the contrast ‘transition revaluation>control condition’ was also larger in the alcohol (OR=65.59, *z*=5.51, *p*<.001) compared to the non-alcohol context (OR=13.08, *z*=3.28, *p*=.001). Moreover, the contrasts ‘reward revaluation>control condition’ ($\beta$=-0.65, *z*=-5.60, *p*<.001), ‘goal-state revaluation>control condition’ ($\beta$=-0.70, *z*=-6.01, *p*<.001), ‘transition revaluation>control condition’ ($\beta$=-0.37, *z*=-2.87, *p*=.017), and ‘policy revaluation>control condition’ ($\beta$=-0.80, *z*=-6.86, *p*<.001) decreased over time in low-risk drinkers, but only the contrast ‘policy revaluation>control condition’ ($\beta$=-0.41, *z*=-3.77, *p*<.001) decreased in high-risk drinkers, whereas the contrasts ‘reward revaluation>control condition’ ($\beta$=-0.15, *z*=-1.39, *p*=.663), ‘goal-state revaluation>control condition’ ($\beta$=-0.20, *z*=-1.87, *p*=.248), and ‘transition revaluation>control condition’ ($\beta$=0.03, *z*=0.21, *p*=1.00) did not. Lastly, the contrast ‘goal-state revaluation>control condition’ decreased more strongly over time in the alcohol context ($\beta$=-0.63, *z*=-5.50, *p*<.001) compared to the non-alcohol context ($\beta$=-0.27, *z*=-2.49, *p*=.013). CI, confidence interval; z-value, Wald test z-value.

# Deviations from preregistration

## Data collection

Due to difficulties in reaching the preregistered sample size on Prolific, we sequentially changed our recruitment criteria. Initially limited to fluent German speakers residing in Germany, the target population was expanded to include fluent German speakers living in other EU countries, and subsequently to fluent English speakers residing in the UK. For the English-speaking sample, we translated multi-stage decision-making task instructions, used GBP instead of Euro bills as rewards in the non-alcohol task context, used standard English versions of questionnaires, and translated screening questions. Both task versions, including all instructions, are available in the manuscript’s GitHub repository. Sample composition is detailed in Table 1.

The preregistered full counterbalancing of condition order and environment order according to a Latin-square design turned out problematic as participants dropped out during the task quiz or were excluded due to preregistered criteria. We thus focused on counterbalancing condition order within each of the four subsamples, guaranteeing that each condition appeared at each index with the same frequency across participants. The combination of condition and environment was tentatively balanced (Figure M), with $\chi^{2}$-tests indicating no significant differences in the number of times each condition was performed in each environment within any of the four subsamples (low-risk drinkers in non-alcohol context: $\chi^{2}$=0.69, p=1; low-risk drinkers in alcohol context: $\chi^{2}$=10.54, p=.837; high-risk drinkers in non-alcohol context: $\chi^{2}$=1.38, p=1; high-risk drinkers alcohol context: $\chi^{2}$=7.23, p=.969). Environment order was not counterbalanced.

After finishing data collection, we discovered minor errors in the AUDIT questionnaire. While we do not suspect these errors to influence our findings, we decided to report them for reasons of transparency. Specifically, in the pre-task AUDIT (German and English version affected) and post-task AUDIT (only German version affected), the fourth answer category to question 2 was described as ‘7 or 8’ instead of ‘7 to 9’, meaning that participants could not indicate they typically drank 9 alcoholic drinks on drinking days. In the pre-task AUDIT administered to English-speaking low-risk drinkers performing the non-alcohol version and to English-speaking high-risk drinkers performing the alcohol version, items 3-8 erroneously contained an additional answer category ‘always’. Responses in this category were recoded as ‘daily or almost daily’ for consistency. Additionally, in the post-task AUDIT administered to English-speaking low-risk drinkers performing the non-alcohol version, item 3 ‘How often do you have six or more drinks on one occasion?‘ was erroneously replaced by a repetition of item 1 ‚How often do you have a drink containing alcohol?‘ with the answer categories for item 3 (plus ‚always‘). As participants likely reach higher scores on item 1 than item 3 and will thus be erroneously categorized as high-risk drinkers according to the post-task AUDIT, this error likely led to exclusion of data from too many participants in this subsample and favored participants with less risky drinking (which is desirable for low-risk drinkers). Lastly, in the post-task AUDIT administered to English-speaking low-risk drinkers performing the alcohol version, the second and third answer category for item 1 were ‘times a month and ‘times a week’ instead of ‘2-4 times a month’ and ‘2-3 times a week’. As participants likely inferred that answers should be ‘a couple times a month/week’, this supposedly led to some unsystematic bias in exclusion which we could not correct for. Importantly, the post-task AUDIT questionnaire served solely to exclude participants whose second response would have placed them in a different group compared to their initial classification based on the pre-task AUDIT.

## Data analysis

To increase comparability with the previous study by Momennejad and colleagues, we reversed the coding of the outcome variable for the control condition only. Specifically, while we preregistered that the primary outcome would be a binary variable “correct path” indicating whether the correct path according to the re-learning phase was chosen, our variable indicated whether a participant chose a different path from the optimal path according to the learning phase. Our outcome is thus similar to the switch in state 1 action reported by Momennejad and colleagues [15]. Importantly, the “correct path” and “changed path” criterion differs for the control condition only and not for any of the revaluation conditions, where a “correct path” after re-learning was always a “changed path” compared to after learning.

We preregistered that group and version would be dummy-coded with low-risk drinkers and the non-alcohol context as reference level. However, during data analysis, it became evident that we could not properly test hypothesis 3 on the effects of group and condition *across* contexts in the full sample, using dummy-contrasts for group and context. This is because the preregistered model included fixed effects for context and its interactions, and effects of group and condition would have had to be interpreted at the reference level, i.e. would concern the non-alcohol context only. As sum contrasts for group and context come with the benefit that condition effects in the full sample can be interpreted at the average of group and context, we used them in all analyses.

Moreover, the preregistered logistic mixed-effects models did not include main effects for context nor two-way interactions of context with group or condition. As our descriptive data revealed clear context effects, we decided to include these effects to avoid misattribution of variance to other specified effects.

To control for individual differences in the contrasts between conditions, we included a by-subject random slope for condition in logistic mixed-effects regression models. This was possible as we had 5 test trials per condition. At the time of preregistration, we favored a random-intercept model to avoid later convergence issues. When analyzing data, however, models with an additional random slope for condition had a significantly better fit than random-intercept models according to Likelihood Ratio Tests in all cases. The more complex random effect structure also allowed us to extract individual slopes used for exploratory analyses.

**Figure P.** **Balancing of condition and environment in the multi-stage decision-making task.**

# References

1.	Koller M. robustlmm: An R package for robust estimation of linear mixed-effects models. Journal of Statistical Software. 2016;1. Available: https://www.jstatsoft.org/v075/i06

2.	Mason F, Cantoni E, Ghisletta P. Linear mixed models and latent growth curve models for group comparison studies contaminated by outliers. Psychological Methods. 2025;30: 155–173. doi:10.1037/met0000643

3.	Saunders JB, Aasland OG, Babor TF, De La Fuente JR, Grant M. Development of the Alcohol Use Disorders Identification Test (AUDIT): WHO Collaborative Project on Early Detection of Persons with Harmful Alcohol Consumption-II. Addiction. 1993;88: 791–804. doi:10.1111/j.1360-0443.1993.tb02093.x

4.	Fragebogeninstrumente. In: alkoholleilinie.de [Internet]. [cited 2 Aug 2025]. Available: https://alkoholleitlinie.de/diagnostik/fragebogen-instrumente/

5.	American Psychiatric Association. Diagnostic and statistical manual of mental disorders. 5th ed. Washington, DC: American Psychiatric Association; 2013.

6.	Alcohol Use Disorder: A Comparison Between DSM–IV and DSM–5. In: National Institute on Alcohol Abuse and Alcoholism (NIAAA) [Internet]. [cited 2 Aug 2025]. Available: https://www.niaaa.nih.gov/publications/brochures-and-fact-sheets/alcohol-use-disorder-comparison-between-dsm

7.	Hildebrandt MK, Dieterich R, Endrass T. Disentangling substance use and related problems: urgency predicts substance-related problems beyond the degree of use. BMC Psychiatry. 2021;21: 242. doi:10.1186/s12888-021-03240-z

8.	Kuitunen-Paul S, Rehm J, Lachenmeier DW, Kadrić F, Kuitunen PT, Wittchen H-U, et al. Assessment of alcoholic standard drinks using the Munich composite international diagnostic interview (M-CIDI): An evaluation and subsequent revision. International Journal of Methods in Psychiatric Research. 2017;26: e1563. doi:10.1002/mpr.1563

9.	Lachner G, Wittchen H-U, Perkonigg A, Holly A, Schuster P, Wunderlich U, et al. Structure, Content and Reliability of the Munich-Composite International Diagnostic Interview (M-CIDI) Substance Use Sections. European Addiction Research. 1998;4: 28–41. doi:10.1159/000018922

10.	Vollstädt-Klein S, Leménager T, Jorde A, Kiefer F, Nakovics H. Development and Validation of the Craving Automated Scale for Alcohol. Alcoholism: Clinical and Experimental Research. 2015;39: 333–342. doi:10.1111/acer.12636

11.	Cyders MA, Smith GT, Spillane NS, Fischer S, Annus AM, Peterson C. Integration of impulsivity and positive mood to predict risky behavior: Development and validation of a measure of positive urgency. Psychological Assessment. 2007;19: 107–118. doi:10.1037/1040-3590.19.1.107

12.	Wüllhorst V, Lützkendorf J, Endrass T. Validation of the German long and short versions of the UPPS-P Impulsive Behavior Scale. Journal of Clinical Psychology. 2024;80: 2099–2116. doi:10.1002/jclp.23724

13.	Foa EB, Huppert JD, Leiberg S, Langner R, Kichic R, Hajcak G, et al. The Obsessive-Compulsive Inventory: Development and validation of a short version. Psychological Assessment. 2002;14: 485–496. doi:10.1037/1040-3590.14.4.485

14.	Gönner S, Leonhart R, Ecker W. Das Zwangsinventar OCI-R - die deutsche Version des Obsessive-Compulsive Inventory-Revised. Psychother Psychosom Med Psychol. 2007;57: 395–404. doi:10.1055/s-2007-970894

15.	Momennejad I, Russek EM, Cheong JH, Botvinick MM, Daw ND, Gershman SJ. The successor representation in human reinforcement learning. Nat Hum Behav. 2017;1: 680–692. doi:10.1038/s41562-017-0180-8

16.	Pedersen ML, Frank MJ, Biele G. The drift diffusion model as the choice rule in reinforcement learning. Psychonomic Bulletin & Review. 2017;24: 1234–1251. doi:10.3758/s13423-016-1199-y

17.	Gläscher J, Daw N, Dayan P, O’Doherty JP. States versus Rewards: Dissociable neural prediction error signals underlying model-based and model-free reinforcement learning. Neuron. 2010;66: 585–595. doi:10.1016/j.neuron.2010.04.016

18.	Lee SW, Shimojo S, O’Doherty JP. Neural computations underlying arbitration between model-based and model-free learning. Neuron. 2014;81: 687–699. doi:10.1016/j.neuron.2013.11.028
